# The cross‐sectional study of hepatic lipase SNPs and plasma lipid levels

**DOI:** 10.1002/fsn3.1403

**Published:** 2020-01-13

**Authors:** Wang Wei, Tian Hu, Huilong Luo, Zhang Ye, Feiteng Lu, Yanqing Wu, Muying Ying

**Affiliations:** ^1^ Department of Molecular Biology and Biochemistry Basic Medical College of Nanchang University Jiangxi China; ^2^ Department of Cardiology The Second Affiliated Hospital of Nanchang University Jiangxi China; ^3^ Department of Emergency and Critical Care Medicine the Second Affiliated Hospital of Nanchang University Jiangxi China

**Keywords:** linkage disequilibrium, LIPC activity, plasma lipid levels, population genetics, SNPs in LIPC promoter

## Abstract

By the combination of meta‐analysis, the data of the 1,000 Genomes Project Phase 3, and the promoter sequence of hepatic lipase (LIPC), we performed the cross‐sectional study to explore the associations of four variants (rs1077835; rs1077834; rs1800588 [C‐514T], and rs2070895 [G‐250A]) in LIPC promoter with plasma lipid levels. Our results indicate that the first and the next three of the four SNPs are, respectively, reported to be associated with the decreased and increased HDL‐c level. Meta‐analysis of 87 studies with 101,988 participants indicates that HDL‐c level in rs1800588 (C‐514T) (pooled mean difference = 0.03, 95%CI (0.03, 0.04), *p* < .001) and rs2070895 (G‐250A) (pooled mean difference = 0.07, 95%CI (0.05, 0.09), *p* < .001) is higher in allele T or A carriers. Similarly, LDL‐c, TC, TG, and BMI levels are generally increased in T or A alleles carriers. We failed to conduct the meta‐analysis of rs1077835 and rs1077834 due to the limited previous reports. Data from the 1,000 Genomes indicate that the allele frequencies of the four SNPs in total or subpopulations are almost equal to each other. The paired value *r* ^2^ and D' of the four SNPs are larger than 0.8, which indicate the linkage disequilibrium of the four variants. The analysis of LIPC promoter indicate that C‐514T and G‐250A are, respectively, located in transcriptional factor binding sites of USF1and Pbx1b, which may partly explain the effect of the two SNPs on the decreased LIPC activity in the alleles carriers and the corresponding increased plasma lipids hydrolyzed by LIPC. These results may help us to better understand the different effects of the four SNPs on the plasma lipid levels among subpopulations and offer clues for future clinical treatment of dyslipidemia‐related diseases.

## Graphical abstract

With 1,000 Genome data, we evaluated the linkage by the combination of meta‐analysis, the data of the 1,000 Genomes Project Phase 3, and the promoter sequence of hepatic lipase (LIPC), we performed the cross‐sectional study to explore the associations of four variants [rs1077835; rs1077834; rs1800588 (C‐514T), and rs2070895 (G‐250A)] in LIPC promoter with plasma lipid levels.

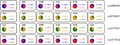

## 1 INTRODUCTION

**Figure 1:**
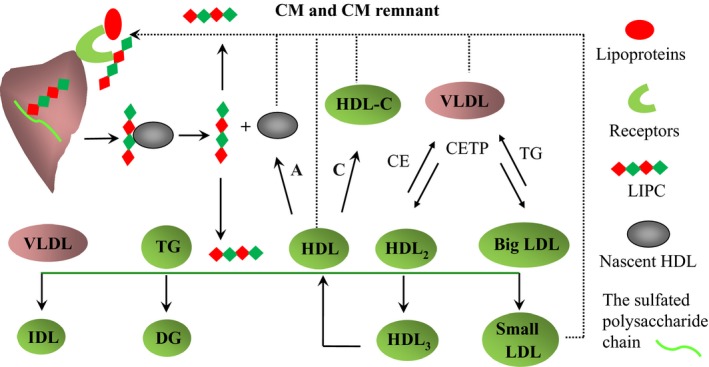
The main process of LIPC functions. LIPC is synthesized in an inactive form through the sulfated polysaccharide chain of a proteoglycan. HDL replaces the polysaccharide chain and carries LIPC into the bloodstream. The dissociation of HDL allows LIPC to hydrolyze VLDL, TG, HDL2, and big LDL into IDL, DG, HDL3, and small LDL, respectively (indicated by solid line). As a ligand, LIPC can be anchored to extracellular to facilitate the removal of lipoprotein remnant and the hepatic uptake of lipoprotein (indicated by dot line) [Receptors: LDL receptor (LDLr), LDLr‐related protein (LRP), or scavenger receptor B1 (SR‐B1); A: Apo A‐I; C: cholesterol; CE: cholesteryl ester; CETP: cholesterol ester transfer protein]

Hepatic lipase (LIPC) is an extracellular liver enzyme that plays important roles in the hydrolysis, transport and intake of plasma lipid and lipoprotein through phospholipase A1, triacylglycerol hydrolase, and ligand‐binding functions. As a lipolytic enzyme, LIPC catalyzes the hydrolysis of triglycerides (TG) and phospholipids (PLs) in several plasma lipoproteins. As a ligand‐binding protein, LIPC facilitates the removal of lipoprotein remnant and the hepatic uptake of lipoprotein by anchoring to extracellular (Diard, Malewiak, Lagrange, & Griglio, 1994; de Faria, Fong, Komaromy, & Cooper, 1996). The main process of LIPC functions is outlined in Figure 1: (a) Nascent LIPC is attached to the vascular surface of hepatocytes and hepatic sinusoid capillaries in an inactive form through the sulfated polysaccharide chain of a proteoglycan; (b) high‐density lipoprotein (HDL) replaces the polysaccharide chain and carries LIPC into the bloodstream; (c) the dissociation of HDL frees LIPC of the lipoprotein on its surface, which results in the activation of LIPC enzyme that hydrolyzes TG and PLs. The main reactions include (a) hydrolyzing TG and converting intermediate‐density lipoprotein (IDL) to low‐density lipoprotein (LDL); (b) hydrolyzing TG and PLs in the larger HDL_2_ particle and converting HDL_2_ to HDL_3_ and then to even smaller HDL particles; (c) hydrolyzing TG and PLs in LDL and HDL after TG‐rich very low‐density lipoprotein (VLDL) exchanging TG for cholesteryl ester (CE) in LDL and HDL; (d) hydrolyzing TG to diacylglycerol (DG) and freeing fatty acids; (4) as a ligand protein, LIPC can facilitate the removal of lipoprotein remnant (chylomicrons, chylomicron remnants, VLDL, LDL, and HDL‐C) and the uptake of lipoproteins into different type cells and directly affects cellular lipid delivery (Santamarina‐Fojo, González‐Navarro, Freeman, Wagner, & Nong, 2004). Cell surface receptors including the LDL receptor, LDLr‐related protein, scavenger receptor B1, and cell surface proteoglycans are implicated to participate on these processes (Komaromy, Azhar, & Cooper, 1996) (Figure 1).

The intricate influence of LIPC in the levels of plasma lipoproteins makes it a debate about whether LIPC acts in a more pro‐ or more antiatherogenic fashion (Goodarzynejad et al., 2017). Among the four variants (rs1077835; rs1077834; rs1800588 [C‐514T], and rs2070895 [G‐250A]) in the proximal sequence of LIPC promoter, SNPs rs1077835 was reported to be related to the decreased HDL‐c level in Mexicans (Weissglas‐Volkov et al., 2013); SNPs rs1077834 was indicated to contribute significantly to plasma HDL‐C levels in weight‐loss‐induced obese individuals (Sarzynski et al., 2011). Compared with the only one publication that reported the association of rs1077835 and rs1077834 with plasmas lipids is, respectively, available, C‐514T and G‐250A were repeatedly reported to be associated with the increased levels of plasma lipids and the decreased LIPC activity (Zambon, Deeb, Hokanson, Brown, & Brunzell, 1998). About 25% of individual variation of LIPC activity is accounted for by the presence of SNPs C‐514T in the promoter of the LIPC gene (Zambon, Deeb, Pauletto, Crepaldi, & Brunzell, 2003). Both C‐514T and G‐250A were allelically associated with insulin resistance and dyslipidemia (Gómez et al., 2005; Pihlajamäki et al., 2000). Based on these observations, a meta‐analysis was carried out to pool eligible results from existing population‐based related studies to further explore the effects of the two SNPs (C‐514T and G‐250A) on LIPC activities and plasma lipid levels, but not rs1077835 and rs1077834 due to the only one related publication, respectively, available that is not enough to calculate (Sarzynski et al., 2011; Weissglas‐Volkov et al., 2013). The analysis of the allele frequency from 1,000 Genomes Project Phase 3 shows that the four SNPs are physically linkage. The molecular effects of C‐514T and G‐250A on the decreased LIPC activity may be partly attributed to the facts that the two SNPs are, respectively, located in two DNA cis‐acting elements: C‐514T in USF1 (upstream stimulatory factor) between −513 and −488, and G‐250A in Pbx1b (Pre‐B‐cell leukemia transcription factor) between −238 and −212. Both transcription factors have been confirmed to affect LIPC transcriptional activities (Rufibach, Duncan, Battle, & Deeb, 2006).

## 2 MATERIALS AND METHODS

### 2.1 Literature search and selection

To understand the developing trends and hot topics of LIPC SNPs, a bibliometric analysis was conducted by CiteSpace R2 (Chen, Hu, Liu, & Tseng, 2012). Keywords searched in TS (terms) or TI (title) in the advances search model of Web of Science included “hepatic lipase,” “LIPC,” “SNPs,” and “single nucleotide polymorphisms.” Publications were limited to English. Based on the results of bibliometric analysis and the Preferred Reporting Items for Systemic Reviews, Meta‐analysis and Meta‐analysis of Observational Studies in Epidemiology recommendations, literature searches were carried out for publications covering the period up to 20 March 2018 at the websites including PubMed, Embase, Cochrane Library, Web of Science, and Ovid. Search strategies included keywords as “LIPC” or “HL,” or “hepatic lipase,” and “polymorphism,” or “variant,” or “SNP.” The publications were limited to human studies without language restrictions. The lists of all identified publications were reviewed and hand‐searched to identify additional studies that may not be captured by the searches. The protocol of this meta‐analysis was registered in PROSPERO (Registered Number: CRD42016046903) that is available from http://www.crd.york.ac.uk/PROSPERO/ display_ record.asp?ID = CRD42016046903.

### 2.2 Inclusion and exclusion criteria

Studies were considered eligible for inclusion based on these criteria: (a) exploring the association between plasma lipids with LIPC SNPs; (b) designed as cohort, case–control, or cross‐sectional studies; and (c) adjusted odds ratio (OR) and 95% confidence intervals (CIs) were reported or could be calculated. In several studies, the adjusted OR was unavailable in the multivariate analysis due to nonsignificant statistical results during univariate analysis. Excluding studies included (a) editorials, letters to the editor, review articles, animal experiments, case reports, and conference abstracts; (b) lipid values were obtained after treatment or management; (c) LIPC combined with other genes; (d) familial combined hyperlipidemia; (e) infrequently reported SNPs; and (f) patients with specific conditions or diseases. Flow diagram for the identification of eligible articles in this meta‐analysis was shown in Figure S1.

### 2.3 Data extraction and outcomes of interest

All the included studies were independently assessed by at least two authors. Disagreements were resolved by the adjudicating senior author (Ying). Articles that could not be categorized in accordance with title and abstract were retrieved for full‐text review. The following variables were extracted from these included studies: title, name of the first author, publication year, sample size, ethnic information, study design, age, gender, adjusted OR (or crude OR) and 95% CI, variables adjusted in the multivariate regression model. The values of HDL‐C, LDL‐C, total cholesterol (TC), TG, LIPC activity, and body mass index (BMI) in different subcategory were calculated and combined to produce the overall index with weights reflecting their shares in the total index (Wan, Wang, Liu, & Tong, 2014). Subgroup analyses were performed by gender and ethnicity. Continuous data in these studies were presented as means and range values. The standard deviation (*SD*) was calculated as that of the previous literature (Wan et al., 2014).

### 2.4 Statistical analysis and quality assessment

Bibliometric analysis was conducted by the relevant function of the Web of Science and CiteSpace Version 5.2.R2 (Chen et al., 2012). Meta‐analysis was performed by Stata Version 12.0 (StataCorp). Weighted mean difference (WMD) summarizes the difference between the two genotypes. All results were reported with 95% confidence interval (CI). The methodological quality of all studies in the meta‐analysis was evaluated by the Newcastle–Ottawa scale (NOS) composed of the following aspects: selection, comparability, and exposure (case–control or cross‐sectional studies) or outcome (cohort studies) (Lo, Mertz, & Loeb, 2014). The maximum score was nine points. Studies with NOS score < 3, 7 > NOS score ≥ 3, and NOS score ≥ 7 were considered to be of poor, median, high quality, respectively.

Statistical heterogeneity among these studies was evaluated by the chi‐square test with significance set at *p* < .10, and heterogeneity was quantified using the *I* ^2^ statistic. The assumption of homogeneity between studies was regarded as invalid if the *p*‐value was <.1 and the random‐effects models were reported after exploring the causes of heterogeneity. Otherwise, the fixed‐effects models were reported. A two‐tailed *p*‐value of <.05 was considered statistically significant. Sensitivity analyses were performed by removing each study in turn to establish the extent to which they contributed to heterogeneity and to the overall result. The potential publication bias was assessed using funnel plot, Begg and Egger tests (Begg & Mazumdar, 1994). A two‐tailed *p*‐value of <.05 was considered statistically significant. The statistical analyses were performed by Stata version 12.0 (StataCorp).

## 3 RESULTS

### 3.1 Bibliometric analysis of references and keywords

**Figure 2:**
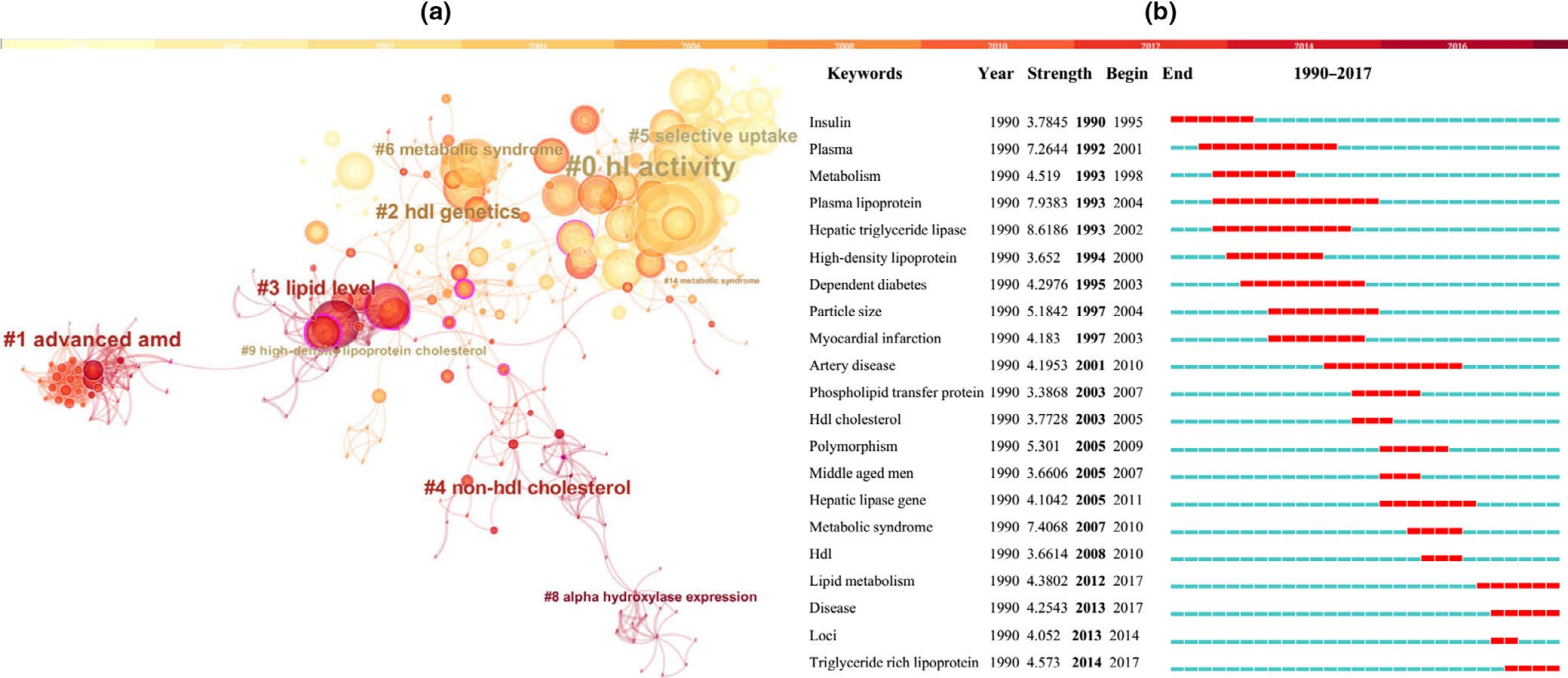
Bibliometric analysis of references and key words. (a) The co‐citation map of references: the network was reasonably divided into loosely coupled 15 clusters. The clusters #6, #7, and #10 were, respectively, labeled as high‐density lipoprotein metabolism, LIPC gene association, and HDL subfractions; (b) Co‐cited clusters. The top 21 keywords with the strongest citation bursts were mainly centered on plasma lipid, polymorphism, HDL‐C, disease, and hepatic triglyceride lipase

A total of 2,482 references were retained for bibliometric analysis of references and keywords. As a significant indicator in bibliometric, the co‐citation map of references suggests the scientific relevance of these references (Figure 2a). With confidence modularity *Q* score and average silhouette score (>0.5), the network was reasonably divided into loosely coupled 15 clusters, and the homogeneity of these clusters was acceptable on average. The clusters #6, #7, and #10 were, respectively, labeled as high‐density lipoprotein metabolism, LIPC gene association, and HDL subfractions, which indicated that the relationships between LIPC and HDL have been investigated in previous publications. About 139 references were retrieved from Web of Science with the searching terms of “LIPC or hepatic lipase” and “SNPs.” Co‐cited clusters view was shown in Figure 2b. The information of these clusters indicated the association between SNPs in LIPC with #6 cardiovascular disease and #7 insulin resistance syndrome. Besides, the molecular effect of SNPs in LIPC has been identified in cluster #2, the majorities of SNPs in LIPC were located in the promoter region of the LIPC gene.

Analyzing keywords with the strongest citation bursts indicated the top 21 keywords with the strongest citation bursts, which were mainly centered on plasma lipid, polymorphism, HDL‐C, disease, and hepatic triglyceride lipase, of which “plasma lipoprotein” had the strongest citation bursts in 1993–2004. While study hot after 2010 mainly include metabolism, disease, loci polymorphism, and HDL (Figure 2b). To better understand the associations of polymorphism, plasma lipid, and LIPC activity, we conducted a systematic review and meta‐analysis of the two SNPs (G‐250A, rs2070895; C‐514T, rs1800588) in LIPC promoter.

### 3.2 Meta‐analysis and subgroup analysis

Eighty‐nine studies with 101,988 participants were included in this meta‐analysis. Seventy‐three studies with 76,798 participants were used to analyze the associations of SNPs C‐514T and plasma lipid levels. Eighteen studies with 25,190 participants were used to analyze the associations of SNPs G‐250A and plasma lipid levels. Two of these studies reported information of both genotypes (Ko, Hsu, Hsu, Ko, & Lee, 2004; Yamada et al., 2007). The general characteristics of these studies were showed in Table S1. Because heterogeneity existed among these studies by chi‐square *Q* tests (*Q* tests for C‐514T: *χ* ^2^ = 371.65, *p* < .001, *I* ^2^ = 78.5%; *Q* tests for G‐250A: *χ* ^2^ = 18.35, *p* = .106, *I* ^2^ = 34.6%), random‐effect model was used to pool overall weighted mean differences (WMDs: kg/m^2^) of the total 95% CI. Subgroup analyses were performed by gender and race. The values of HDL‐C, LDL‐C, TC, TG, LIPC activity, and BMI in different subcategories were calculated and combined to produce the overall index with weights to reflect the shares of their total index among subcategories. Some of these values showed a statistic difference, while others lacked statistic difference between the carriers and the noncarriers among different subgroups. Detailed information of all subgroups analyses was showed in Tables 1 and 2, and the relevant forest plots were shown in Figures [Link], [Link] due to the limited length of this study. These results indicated that the two SNPs of C‐514T and G‐250A were associated with the increased levels of HDL‐c, TG, and TC, and the decreased LIPC activity.

**Table 1:** Results of meta‐analyses for carriers of SNP rs1800588 (C‐514T)

Outcomes	Total		East Asian		White Caucasian		Female		Male	
	WMD	*p*	WMD	*p*	WMD	*p*	WMD	*p*	WMD	*p*
TT + CT versus CC										
HDL‐c	0.05 (0.03,0.07)	**<.001**	0.05 (0.02,0.07)	**<.001**	0.04 (0.01,0.06)	**<.001**	0.07 (0.03,0.1)	**<0.001**	0.05 (0.03,0.07)	**<.001**
LDL‐c	0.07 (0.01,0.14)	**.022**	0.07 (0.00,0.14)	**.046**	0.06 (−0.05,0.16)	.302	0.05 (−0.00,0.09)	0.126	0.06 (−0.02,0.13)	.051
TC	0.12 (0.06,0.18)	**<.001**	0.11 (0.02,0.19)	**.014**	0.09 (−0.02,0.19)	**.021**	0.14 (0.09,0.2)	**<0.001**	0.1 (−0.01,0.21)	.074
TG	0.01 (−0.03,0.04)	.739	0.03 (−0.01,0.08)	.108	0.01 (−0.05,0.07)	.774	0.02 (−0.03,0.07)	0.481	0.01 (−0.04,0.07)	.643
BMI	−0.45 (−0.74,−0.16)	**.002**	NA	NA	NA	NA	−0.45 (−0.83,−0.07)	**0.021**	−0.5 (−1.12,0.13)	.119
HL activity^1^	−57.18 (−88.96,−25.39)	**<.001**	NA	NA	NA	NA	NA	NA	NA	NA
CT versus CC										
HDL‐c	0.03 (0.03,0.04)	**<.001**	0.04 (0.02,0.06)	**<.001**	0.03 (0.02,0.05)	**<.001**	0.03 (0.01,0.04)	**<0.001**	0.04 (0.02,0.05)	**<.001**
LDL‐c	0.02 (−0.00,0.04)	.063	−0.04 (0.09,0.00)	**<.001**	0.05 (0.03,0.07)	.069	0.04 (0.01,0.08)	**0.02**	0.05 (0.02,0.08)	**.003**
TC	0.06 (0.01,0.11)	**.017**	−0.05 (−0.17,0.08)	.45	0.09 (0.03,0.15)	**.003**	0.05 (−0.01,0.1)	0.086	0.06 (−0.04,0.16)	.247
TG	0.03 (0.01,0.05)	**<.001**	0.06 (−0.03,0.14)	.208	0.04 (0.01,0.06)	**.002**	0.04 (0.00,0.08)	**0.041**	0.05 (0.01,0.08)	**.018**
BMI	0.05 (−0.16,0.25)	.649	0.08 (−0.29,0.45)	.67	0.13(−0.1,0.37)	.263	−0.42 (−0.84,0.01)	0.053	0.08 (−0.41,0.56)	.758
HL activity^1^	−44.77 (−70.76,−18.79)	**<.001**	NA	NA	NA	NA	NA	NA	NA	NA
HL activity^2^	−7.84 (−10.17,−5.50)	**.001**	NA	NA	NA	NA	NA	NA	NA	NA
TT versus CT										
HDL‐c	0.06 (0.04,0.08)	**<.001**	0.19 (0.1,0.28)	**<.001**	0.07 (0.04,0.1)	**<.001**	0.06 (0.04,0.09)	**<0.001**	0.07 (0.04,0.11)	**<.001**
LDL‐c	0.01 (−0.05,0.06)	.784	0.02 (−0.03,0.08)	.534	0.06 (−0.13,0.25)	.401	−0.02(−0.11,0.08)	0.729	0.04(−0.09,0.17)	.566
TC	0.11 (0.03,0.18)	**.004**	0.14 (0.02,0.27)	**.028**	0.09 (−0.03,0.21)	.123	0.04 (−0.06,0.14)	0.392	0.17 (0.03,0.31)	**.02**
TG	0.04 (−0.02,0.10)	.169	0.05 (−0.01,0.11)	.087	0.00 (−0.08,0.09)	.911	0.05 (−0.01,0.1)	0.112	−0.03 (−0.16,0.1)	.674
BMI	−0.14 (−0.48,0.19)	.392	−0.05 (−0.71,0.61)	.883	−0.3 (−0.82,0.23)	.271	−0.59 (−1,−0.18)	**0.005**	−0.02 (−0.87,0.83)	.968
HL activity^1^	−94.48 (−139.98,−48.97)	**.017**	NA	NA	NA	NA	NA	NA	NA	NA
HL activity^2^	−6.46 (−11.78,−1.13)	**<.001**	NA	NA	NA	NA	NA	NA	NA	NA
TT versus CC										
HDL‐c	0.1 (0.07,0.12)	**<.001**	0.11 (0.07,0.14)	**<.001**	0.11 (0.06,0.15)	**<.001**	0.09 (0.05,0.12)	**<0.001**	0.12 (0.07,0.17)	**<.001**
LDL‐c	0.01 (−0.06,0.07)	.87	−0.02 (−0.09,0.05)	.997	−0.00 (−0.11,0.11)	.552	−0.06 (−0.21,0.08)	0.378	0.02 (−0.12,0.17)	.766
TC	0.13 (0.03,0.23)	**.018**	0.05 (−0.05,0.14)	.322	0.13 (−0.03,0.29)	.114	0.03 (−0.12,0.17)	0.692	0.19 (−0.02,0.4)	.08
TG	0.08 (0.01,0.15)	**.022**	0.1 (−0.03,0.23)	.136	0.01 (−0.08,0.1)	.815	0.07 (0.00,0.14)	**0.036**	−0.00 (−0.16,0.16)	0.986
BMI	−0.21 (−0.59,0.17)	.286	0.23 (−0.69,1.14)	.629	−0.2 (−0.68,0.28)	.404	−1.17 (−1.64,−0.69)	**<0.001**	−0.05 (−0.78,0.68)	.9
HL activity^1^	−140.37 (−166.01,−114,73)	**<.001**	NA	NA	NA	NA	NA	NA	NA	NA
HL activity^2^	−14.35 (−21.21,−7.50)	**<.001**	NA	NA	NA	NA	NA	NA	NA	NA

Notes

HL activity^1^ was measured by the method provided by Blades et al. (1993). HL activity^2^ was measured by the method provided by Iverius and Brunzell (1985). The values with statistic difference were indicated by bold letters for easy identification.

Abbreviation: NA, not available.

**Table 2:** Results of meta‐analyses for carriers of SNP rs2070895 (G‐250A)

Outcomes	Total		East Asian		White Caucasian	
	WMD	*p*	WMD	*p*	WMD	*p*
AA + GA versus GG						
HDL‐c	0.03 (−0.00,0.17)	**.003**	0.12 (0.03,0.21)	.074	0.03 (−0.01,0.08)	.185
LDL‐c	−0.02 (−0.13,0.09)	.715	−0.08 (−0.19,0.04)	.194	0.11 (−0.09,0.32)	.265
TC	0.03 (−0.08,0.13)	.645	0.09 (−0.06,0.25)	.227	0.16 (−0.21,0.54)	.387
TG	0.06 (−0.05,0.18)	.272	0.08 (−0.07,0.24)	.297	0.09 (−0.01,0.19)	.076
BMI	0.16 (−0.27,0.59)	.466	NA	NA	NA	NA
GA versus GG						
HDL‐c	0.07 (0.05,0.09)	**<.001**	0.06 (0.04,0.08)	**<.001**	0.07 (0.05,0.1)	**<.001**
LDL‐c	0.03 (−0.04,0.11)	.378	0.11 (0.01,0.21)	**.036**	−0.00 (−0.1,0.09)	.932
TC	0.05 (−0.02,0.12)	.159	0.13 (0.00,0.26)	**.043**	0.01 (−0.08,0.09)	.841
TG	−0.06 (−0.17,0.05)	.257	0.00 (−0.1,0.11)	.974	−0.1 (−0.24,0.04)	.153
BMI	0.04 (−0.21,0.3)	.734	NA	NA	NA	NA
AA versus GA						
HDL‐c	0.04 (0.02,0.07)	**<.001**	0.07 (0.05,0.1)	**<.001**	0.01 (−0.02,0.04)	.558
LDL‐c	−0.03 (−0.16,0.09)	.592	−0.06 (−0.2,0.07)	.358	−0.00 (−0.2,0.19)	.973
TC	0.03 (−0.05,0.1)	.473	0.01 (−0.13,0.14)	.904	0.07 (−0.06,0.2)	.319
TG	0.02 (−0.2,0.23)	.873	0.1 (−0.12,0.31)	.371	0.00 (−0.35,0.35)	.984
BMI	0.05 (−0.28,0.38)	.771	NA	NA	NA	NA
AA versus GG						
HDL‐c	0.11 (0.08,0.13)	**<.001**	0.13 (0.08,0.17)	**<.001**	0.09 (0.06,0.12)	**<.001**
LDL‐c	−0.00 (−0.12,0.11)	.961	0.04 (−0.18,0.26)	.719	−0.03 (−0.18,0.11)	.647
TC	0.11 (0.04,0.18)	**<.001**	0.14 (−0.03,0.3)	.105	0.1 (0.02,0.18)	**.012**
TG	0.04 (−0.22,0.3)	.77	0.12 (−0.03,0.27)	.128	0.01 (−0.41,0.44)	.952
BMI	0.02 (−0.3,0.34)	.896	NA	NA	NA	NA

Notes

The values with statistic difference were indicated by bold letters for easy identification.

Abbreviation: NA, not available.

For SNP C‐514T (Table 1), the overall HDL‐c WMD was 0.1 (95% CI 0.07–0.12; *p* < .001), suggesting the allele T carriers had a higher plasma HDL‐c level than noncarriers. Besides, in comparison between TT/CC genotype subjects, other outcomes also demonstrated significant difference (TC: WMD = 0.13, 95% CI 0.03–0.23, *p* = .018; TG: WMD = 0.08, 95% CI 0.01–0.15, *p* = .022; HDL‐c: WMD = 0.04, 95% CI 0.01–0.07, *p* = .018) (Table 1). With the exception of TC in CT versus CC East Asian and TG in male, TC (pooled mean difference = 0.06, 95%CI (0.01, 0.11), *p* < .001) and TG (pooled mean difference = 0.07, 95%CI (0.05, 0.09), *p* < .001) in C‐514T were higher than those of noncarriers. With the exception of White Caucasian (*p* > .05), LDL‐c in C‐514T was higher than that of carriers (pooled mean difference = 0.02, 95%CI (−0.00, 0.04), *p* = .061).

For SNP G‐250A (Table 2), the pooled HDL‐c WMD was 0.11 (95% CI 0.08–0.13; *p* < .001), suggesting the mean plasma HDL‐c level was higher in subjects with AA genotype than subjects with GG genotype. In other genotype comparisons, mean plasma HDL‐c levels were higher in all subjects with homozygous allele A or A carriers (AA/GA: WMD = 0.04, 95% CI 0.02–0.07, *p* = .001; GA/GG: WMD = 0.07, 95% CI 0.05–0.09, *p* < .001; AA + AG/GG: WMD = 0.04, 95% CI 0.00–0.08; *p* = .042) (Table 2). LDL‐c in G‐250A carriers was higher than those of noncarriers in East Asian (LDL‐c, pooled mean difference = 0.11, 95%CI (0.01, 0.21), *p* = .036; TC, pooled mean difference = 0.13, 95%CI (0.00, 0.26), *p* = .043). In addition, subjects with AA genotype had a higher mean plasma TC level than that of subjects with GG genotypes in White Caucasian (AA/GG) (WMD = 0.11, 95% CI 0.04–0.18, *p* = .001). The A carriers had a higher mean plasma TG level than that of the noncarriers (WMD = 0.11, 95% CI 0.02–0.19, *p* = .013) (Table 2).

Hepatic lipase activities were reported in 12 publications. Data from the only two studies of G‐250A could not be pooled because of different detecting assays (Lindi et al., 2008; Zambon et al., 1998). Other 10 studies were evaluated for the difference of LIPC activities between two genotype subjects (Bos et al., 2005; Brinkley et al., 2011; Carr, Ayyobi, Murdoch, Deeb, & Brunzell, 2002; Carr et al., 2001; Dugi et al., 2001; Hodoğlugil, Williamson, & Mahley, 2010; Nie et al., 1998; Vega, Clark, et al., 1998; Vega, Gao, et al., 1998; Zambon, Deeb, Brown, Hokanson, & Brunzell, 2001). The results were shown in two groups because of the difference in assay detecting LIPC activity (Blache, Bouthillier, & Davignon, 1983; Blades, Vega, & Grundy, 1993; Iverius & Brunzell, 1985). LIPC activity in carriers of C‐514T was lower than that of noncarriers (pooled mean difference=−57.18, 95%CI (−88.96, −25.39), *p* < .001). The detailed meta‐analysis results and forest plots were demonstrated in Tables 1 and 2 and Figure S2.

### 3.3 Sensitivity analysis and bias assessment

**Figure 3:**
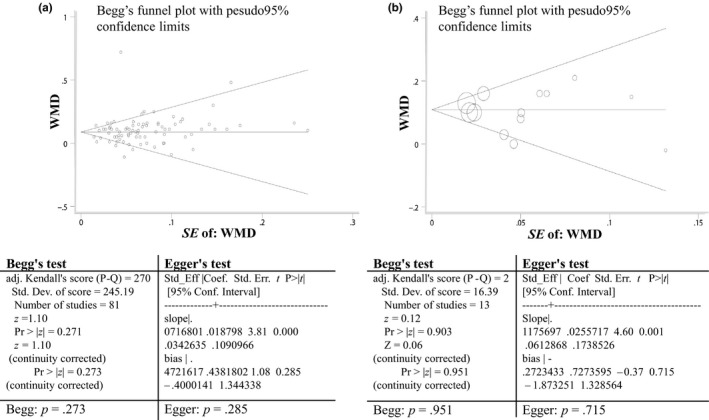
Tests for publication bias. Begg's funnel plot, Begg's test, and Egger's test showed no evidence of publication bias. (a) HDL‐c of TT versus CC (C‐514T SNP rs1800588); (b) HDL‐c of AA versus GG (G‐250A SNP rs2070895)

The results were not changed dramatically after the removal of any data set according to sensitivity analysis by Stata version 12.0 (StataCorp) (Figure S5). No publication bias for the HDL‐c level between AA genotype and GG genotype in SNP G‐250A and between TT genotype and CC genotype in SNP C‐514T was identified by Begg's funnel plot (AA/GG, *p* = .951; TT/CC, *p* = .273) and Egger's regression test (AA/GG, *p* = .715; TT/CC, *p* = .285) (Figure 3a,b).

### 3.4 Population genetics

**Figure 4:**
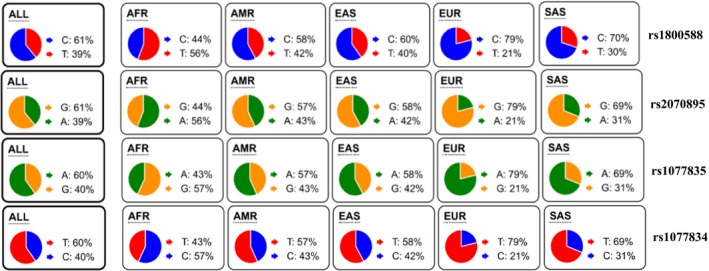
The allele frequencies of the four SNPs in population genetics. The allele frequencies of the four variants between total population or subpopulations are almost equal to each other (All: total population; AFR, African population; AMR, American population; EAS, East Asian population; EUR, European population; SAS, South Asian population)

**Figure 5:**
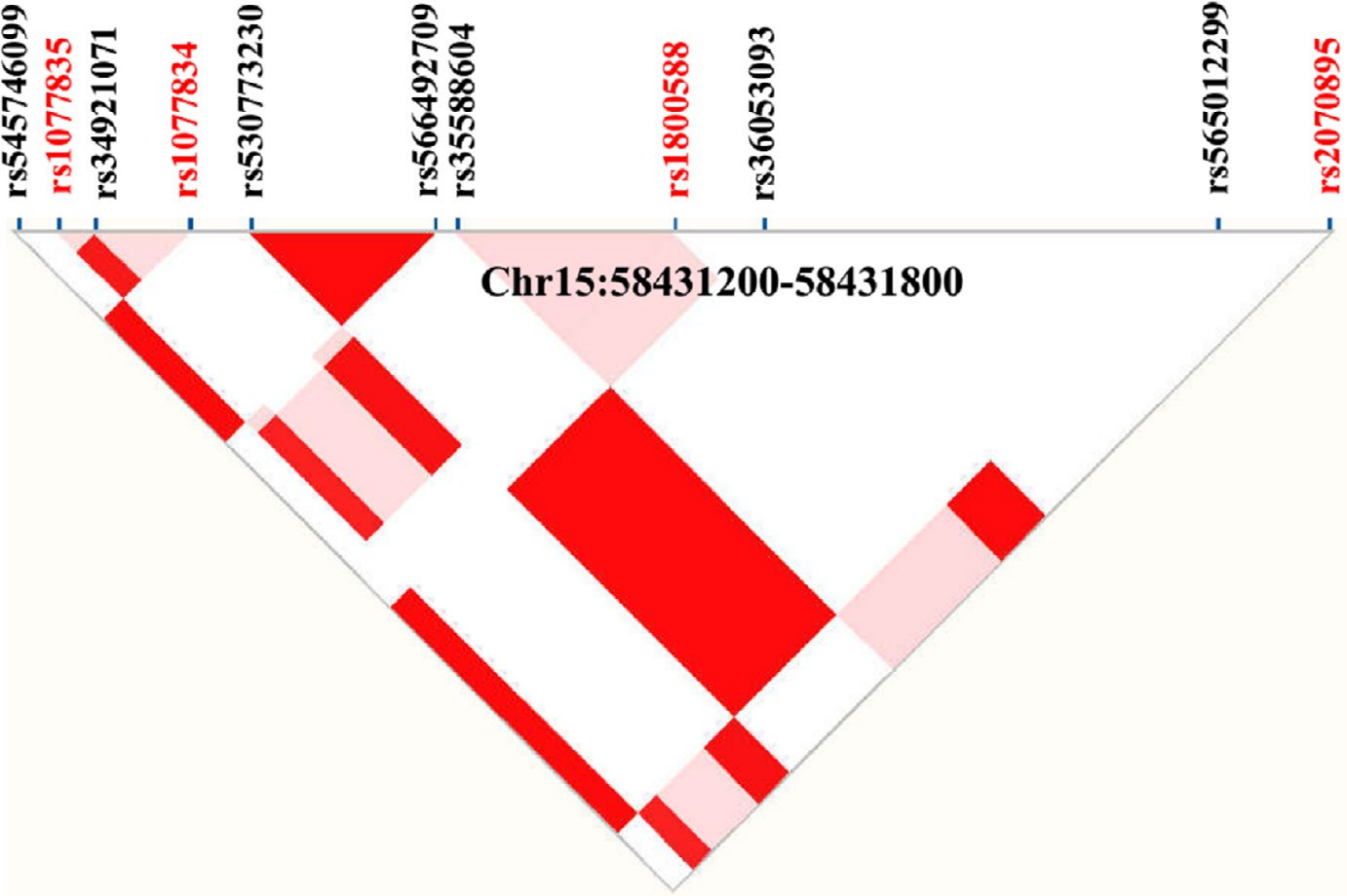
The linkage disequilibrium of the four SNPs. The paired calculation (*r* ^2^ and D') of the four SNPs (indicated by red letters for easy identification) indicated that the values of both *r* ^2^ and D' are larger than 0.8. For variants rs34921071 and rs35588604, the paired estimate of *r* ^2^ is less than 0.8, but D’ >0.8. For other SNPs, the estimated *r* ^2^ and D' are values has a minor allele frequency close or equal to 0 that have been filtered out

Population structure is crucial to study genetic association of subpopulation. The analysis of the 1,000 Genomes Project Phase 3 revealed that the allele frequencies of the four SNPs (rs1077835; rs1077834; rs1800588: C‐514T, and rs2070895: G‐250A) in total population or subpopulations are almost equal to each other, namely distributed in a similar position (Figure 4). The paired calculation (*r* ^2^ and D') of the four SNPs indicated that the estimated values of both *r* ^2^ and D' are larger than 0.8 (Figure 5). Therefore, the four SNPs are physically linked, present obvious chain imbalance (Botma, Verhoeven, & Jansen, 2001).

## 4 DISCUSSION AND CONCLUSIONS

**Figure 6:**
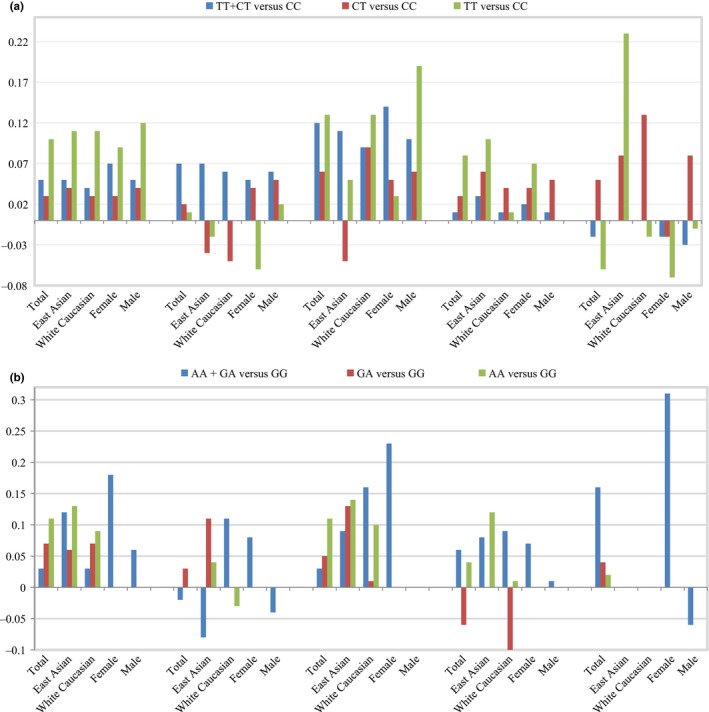
Results of meta‐analyses and subgroup analyses. (a) SNP rs1800588 (C‐514T); (b) SNP rs2070895 (G‐250A). The general trends of LDL‐c, TC, TG, and BMI levels in the two SNPs are increased, although subgroup analyses demonstrate a slight difference

Among the four SNPs, C‐514T and G‐250A are repeatedly reported to be associated with the increased HDL‐c level, which does not appear to actually protect individuals from coronary artery disease (van Acker et al., 2008). While variant rs1077835 (located at −713 upstream of transcript start site) and rs1077834 (located at −660 upstream of transcript start site) were, respectively, reported to be associated with the decreased HDL‐c level in Mexicans (Weissglas‐Volkov et al., 2013), and the weight‐loss‐induced declined HDL‐c in the Swedish obese subjects (Sarzynski et al., 2011). Our results indicate that the heterozygous or homozygous mutation in C‐514T substitution has dosage effect on the increased HDL‐c level, and the T alleles in C‐514T is associated with the increased level of plasma TG and TC (Figure 4), which both update the traditional understanding about SNPs C‐514T effects on plasma lipid levels. LIPC activity was negatively correlated with (LDL) triglyceride in both gout patients and control subjects (Tsutsumi, Yamamoto, Moriwaki, Takahashi, & Hada, 2001). G‐250A is associated with high serum LDL‐c concentration and low LIPC activity (Lindi et al., 2008). In alleles carriers of the two SNPs, LIPC activity is decreased and LDL‐c, TC, TG, and BMI levels are increased correspondingly, although subgroup analysis of the two SNPs demonstrate a slight difference (Figure 6).

**Figure 7:**
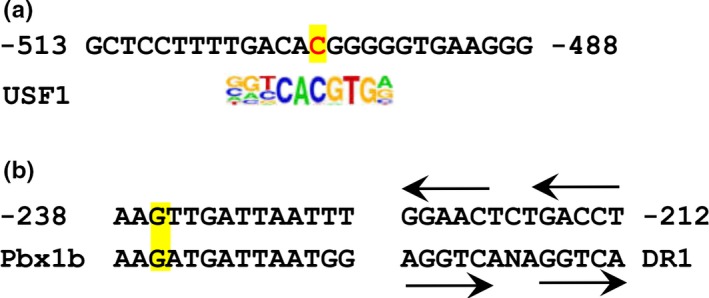
The two SNPs located in transcriptional factor binding sites in LIPC promoter. (a) C‐514T located in USF1; (b) G‐250A located in Pbx1b

To further explore the possible association of the two SNPs with the transcriptional regulation of LIPC expression, we analyzed the proximal sequence of LIPC promoter. And our results show that C‐514T and G‐250A SNPs are, respectively, located in the transcriptional factor binding sites of USF1 (Figure 7a) and Pbx1b in LIPC promoter (Figure 7b). USF1 is one of the positive transcription factor regulating LIPC expression. LIPC activity is reported to be reduced in the C‐514T and G‐250A substitutions of LIPC promoter and increased by the expression of USF1 (Botma et al., 2001). Next to Pbx1b, the inverted direct repeat (DR1) is located in DNase I footprints (Hadzopoulou‐Cladaras & Cardot, 1993), which conform to the consent DNA response element half‐site 59‐RG(G/T)TCA‐39 (Figure 7b). And the transcriptional factor HNF4a can activate LIPC transcriptional expression through the DR1 and DR4 elements in LIPC proximal promoter (Parviz et al., 2003).

The two SNPs of C‐514T and G‐250A are located in the transcriptional factor binding sites of USF1 and Pbx1b in LIPC promoter, which at least partly explain the observations that LIPC activity is decreased in the alleles carriers and the corresponding increased plasma lipids hydrolyzed by LIPC. As a lipolytic enzyme and ligand‐binding protein, the decreased LIPC activity is not sufficient to completely catalyze the hydrolysis of plasma lipid and lipoprotein, and to fully facilitate the removal of lipoprotein remnant and the hepatic uptake of lipoprotein, which should be accompanied by the increased plasma lipid levels in A or T carriers. These results may help us to better understand the different effects of the four SNPs on the plasma lipid levels among subpopulations and offer new thoughts for clinical treatment of dyslipidemia.

## CONFLICT OF INTEREST

The authors declare that they do not have any conflict of interest.

## AUTHORS' CONTRIBUTIONS

M.Y. was responsible for experimental design and manuscript writing. W. W. analyzed these data. H. T. and L. H. prepared Figures 1, 2, 3, 4, 5. Z. Y., L. F., and W. Y. prepared Figures 6, 7 and supplementary materials. All authors read and approved the final manuscript.

## ETHICAL APPROVAL

Ethical Review: This study does not involve any human or animal testing.

Informed Consent: Written informed consent was obtained from all study participants.
