# Supplementary material for: The cross‐sectional study of hepatic lipase SNPs and plasma lipid levels
Source: Food Sci Nutr. 2020 Jan 13;8(2):1162–72. doi: 10.1002/fsn3.1403 (PMC7180388; doi:10.1002/fsn3.1403)
Supplement: Supplementary file 1 [file FSN3-8-1162-s001.docx]

**Figure S1.** Flow diagram for the identification of eligible articles in this meta-analysis.

n=1660 Studies identified from the databases:

PubMed:n=405; Embase:n=261; Ovid:n=432

Web of Science:n=228; Cochrane:n=334

**Identification**

Excluded n=1530

Duplicate studies: 490

Screening title and abstract or

full text with in clusion criteria and no relevant content: 1040

n=130 Full-text studies

assessed for eligibility

Excluded n=28

n=4 Familial combined hyperlipidemia

n=4 Mixed with other genes

n=10 loci other than

rs1800588 or rs2070895

n=1 [Genotyping](https://www.baidu.com/link?url=MhM2xUat4ziTBhSWkK3vMFY5JcsfdfoUpgTwRLuj1TBmJa4fY4xGlenXLjOBVfYyzHUPy3vumI-wFU2anL3JHK_6dbbG7Qmk8W_7tUSCuxm&wd=&eqid=daae262000007df10000000457c5326c) CC+CT vs TT

n=2 SNPs association with lipid size

n=7 other reasons

**Screening**

n=102 Studies relevant to rs1800588 or rs2070895

and lipid level were for data extraction

n=13Studies sample not clear or p (HWE) < 0.5

n=89 Studies (71 for C-514T;16 for G-250A ; 2 for both

C-514T and G-250A) were eligible in the meta analysis

**Eligibility**

**Figure S2A** Forest plots of HDL-c in C-514T

**CT/CC TT/CT**


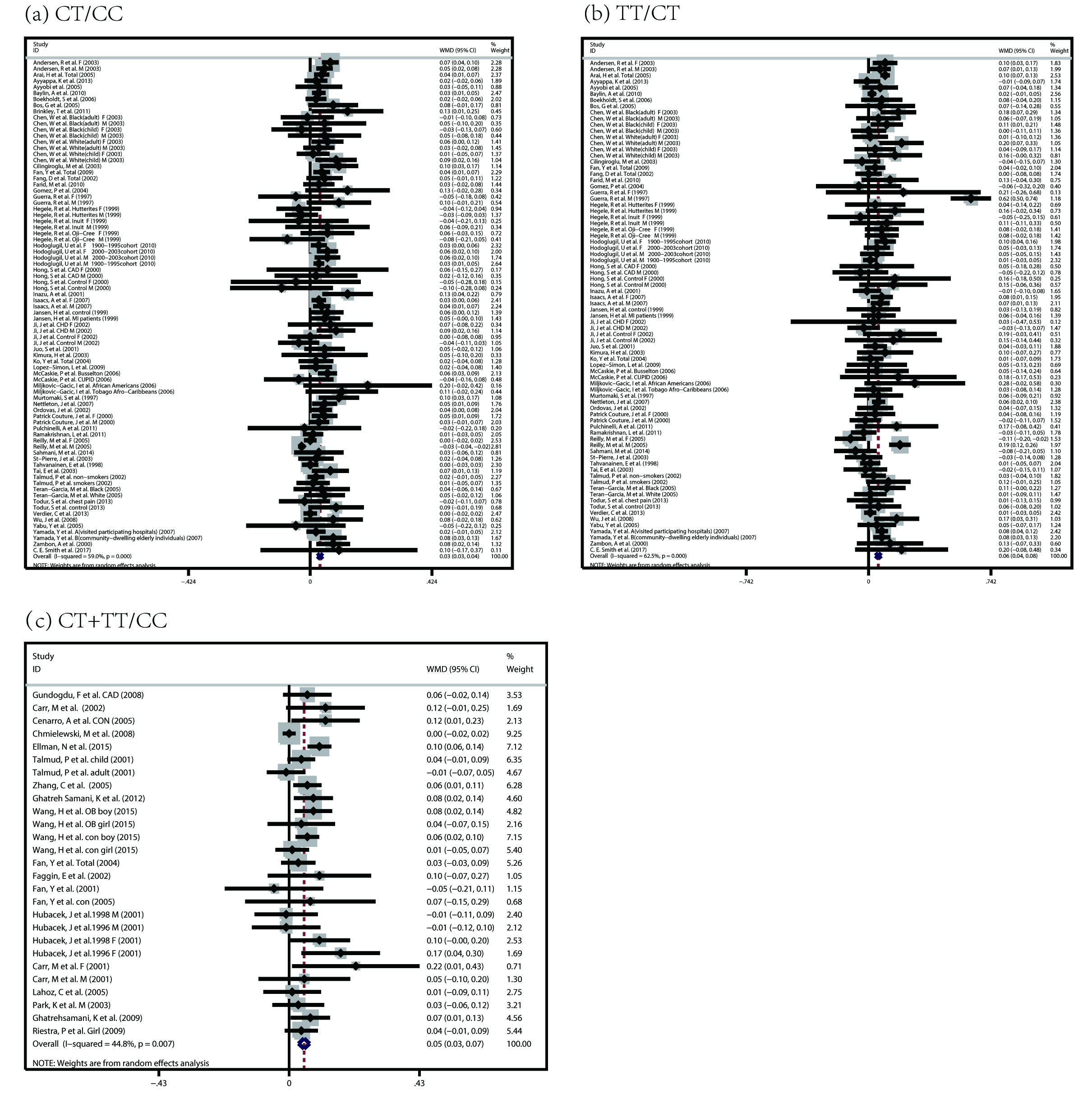

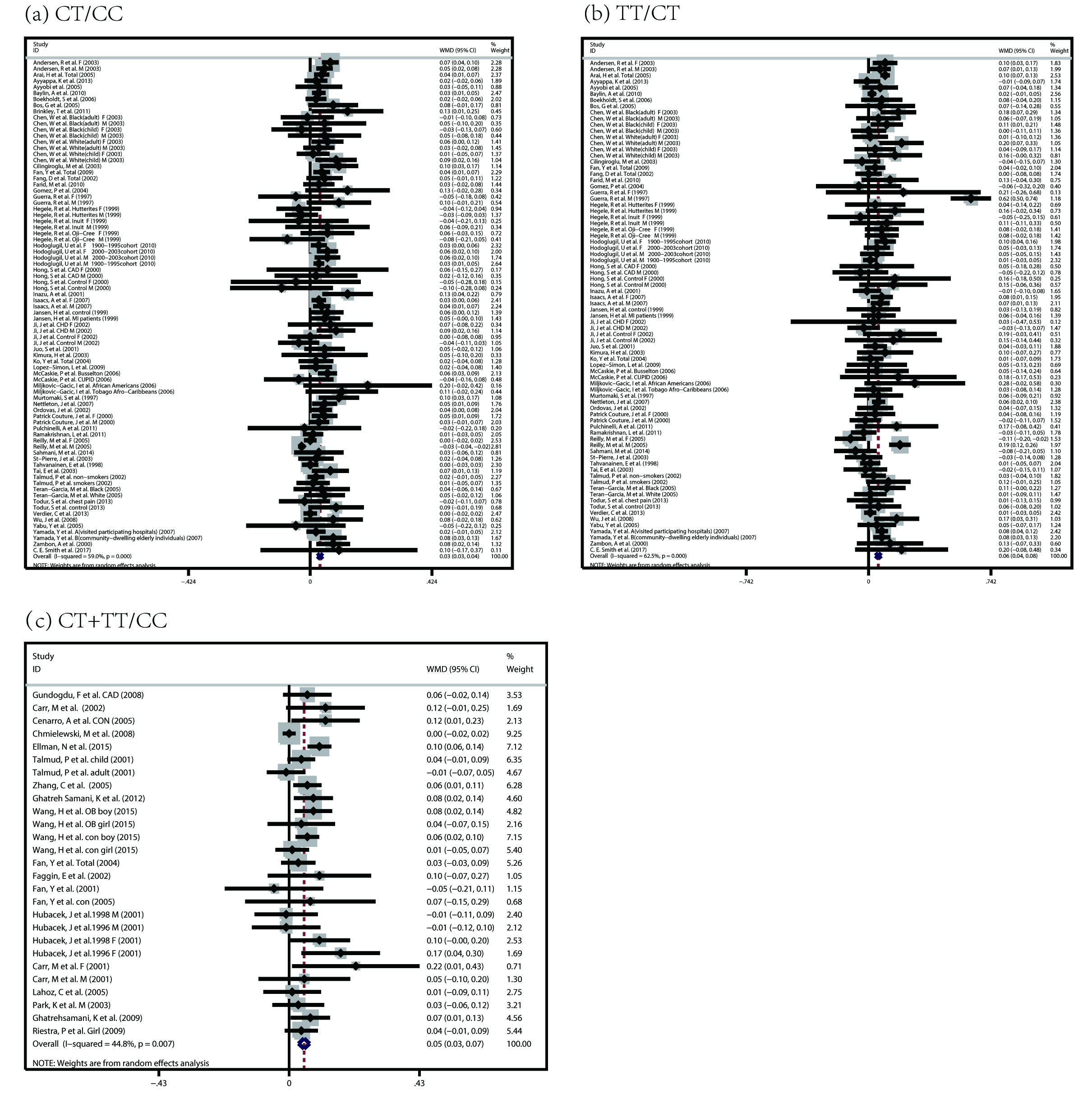


-.424 0 .424 -.742 0 .742

**
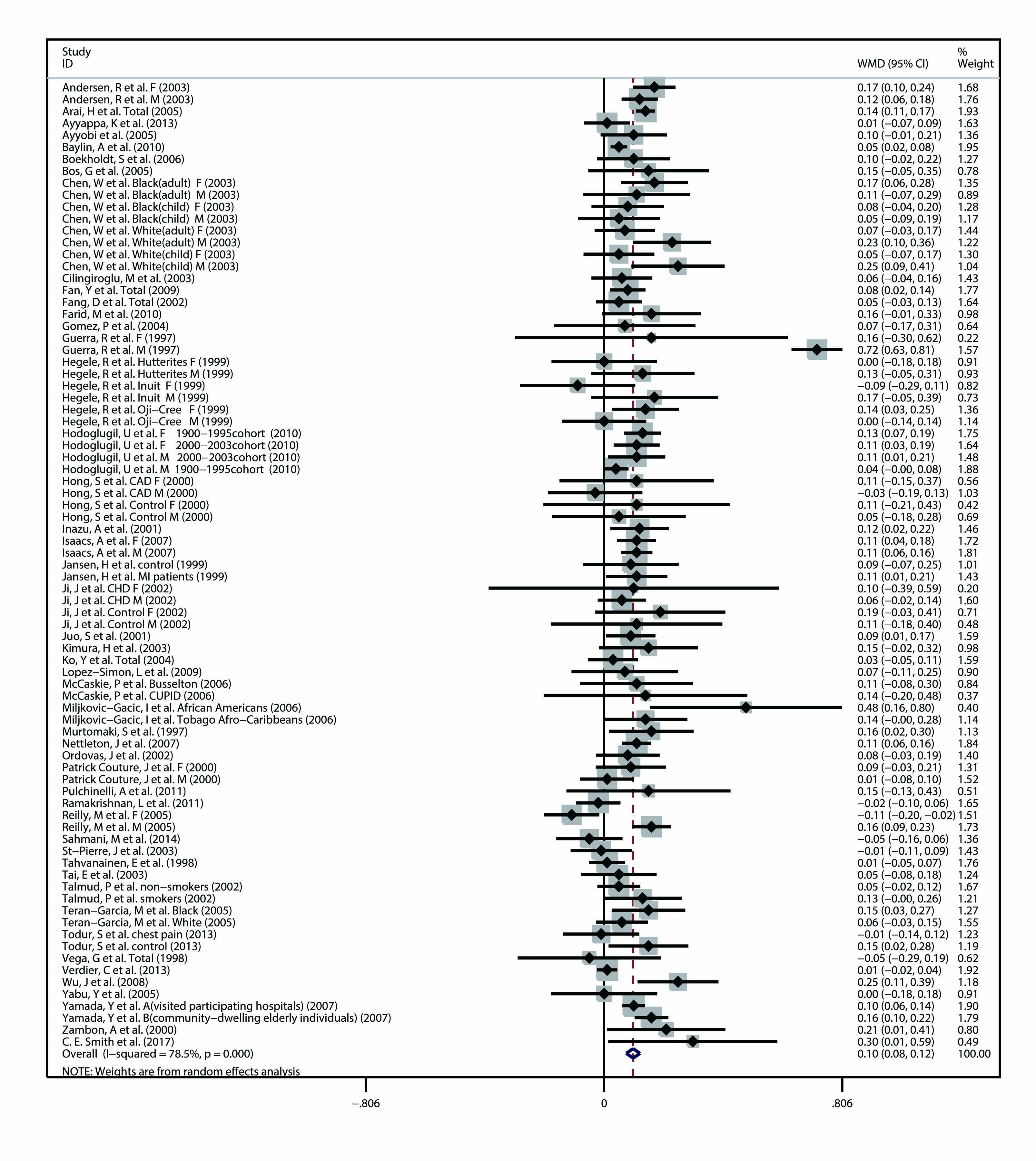

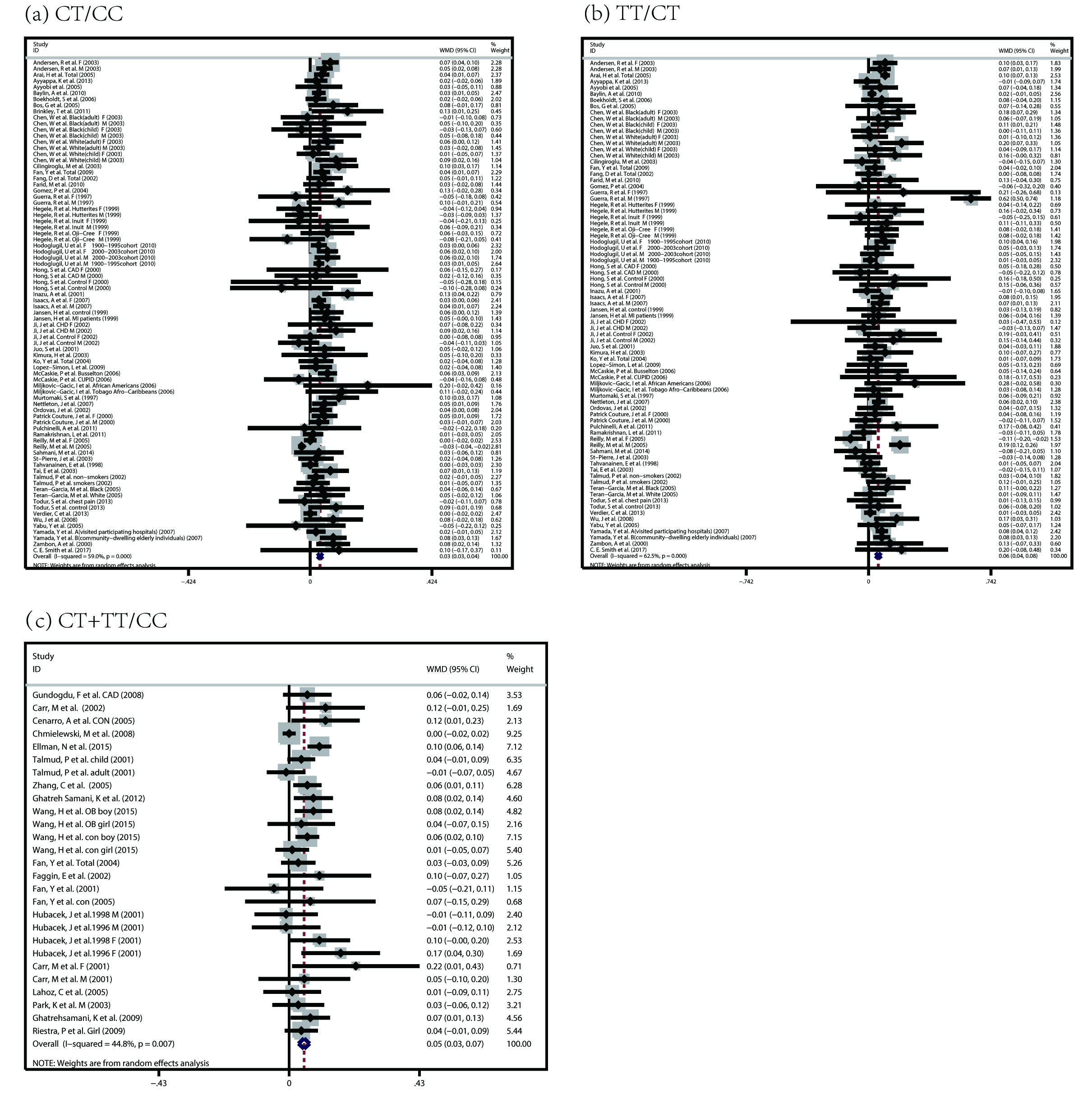
**

**A (TT vs CC)**

**CT +TT/CC**

-.43 0 .43

-.806 0 .806

**Figure S2B** Forest plots of LDL-c in C-514T


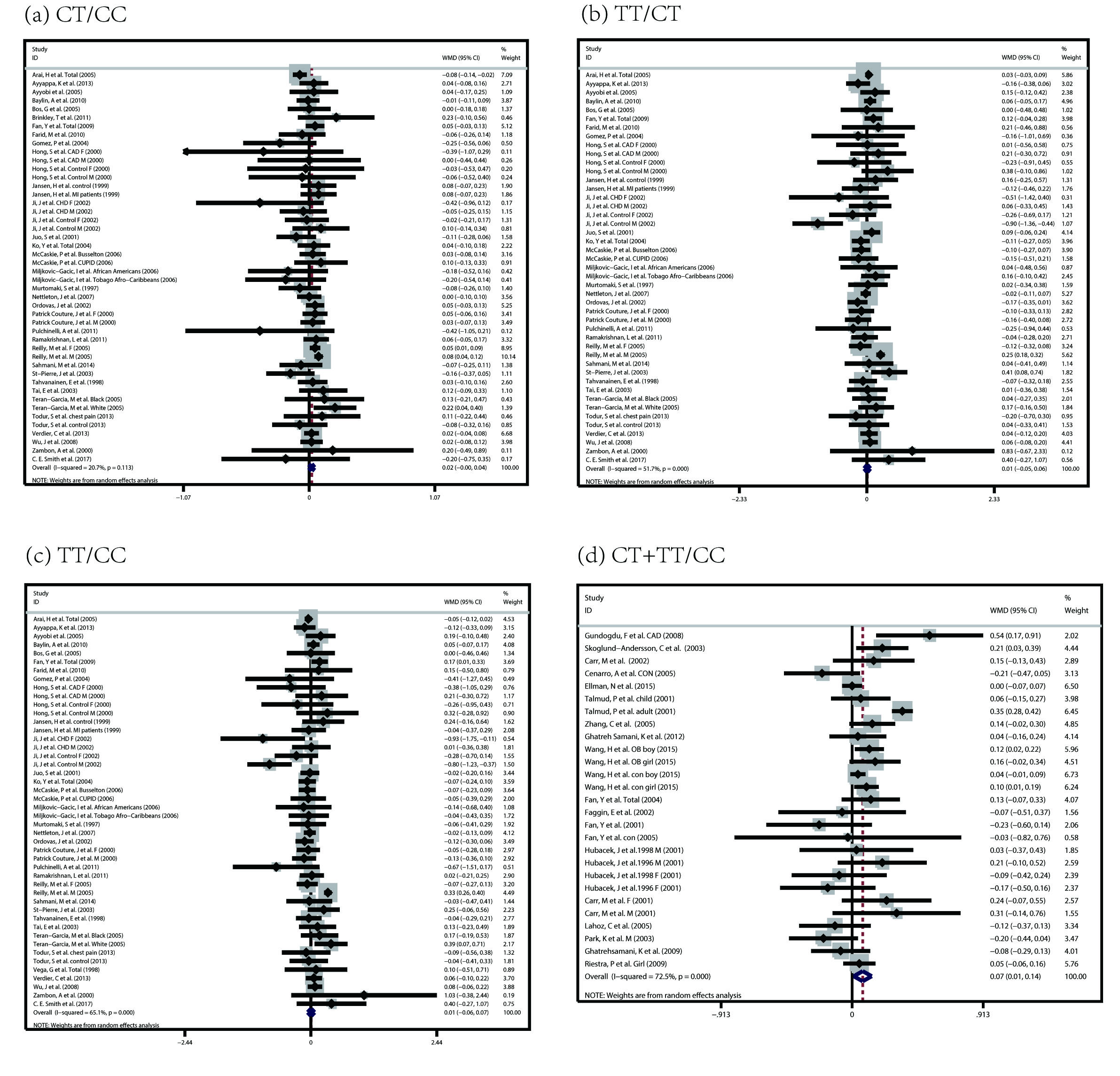


**CT/CC TT/CT**

-1.07 0 1.07 -2.33 0 2.33


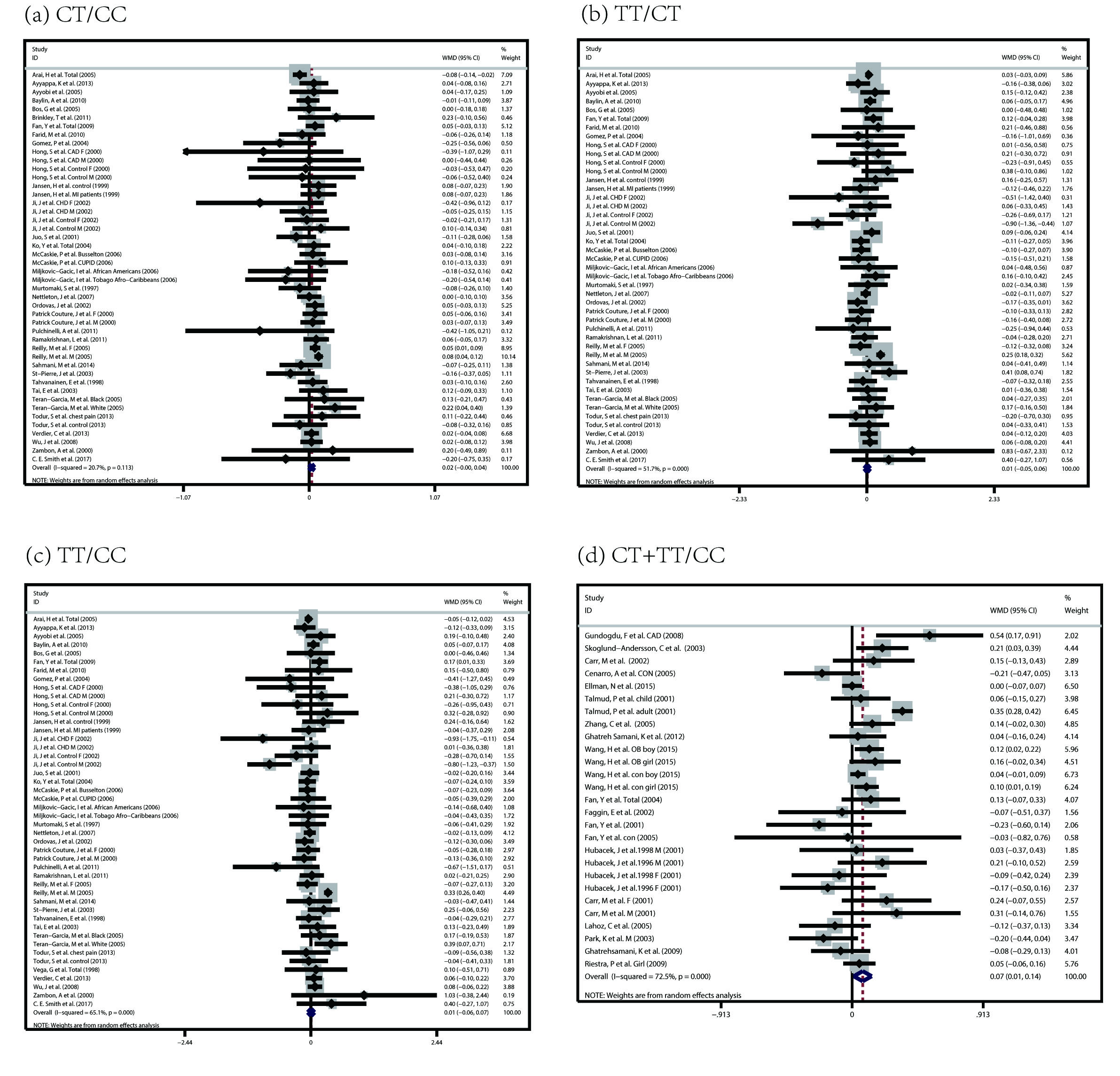

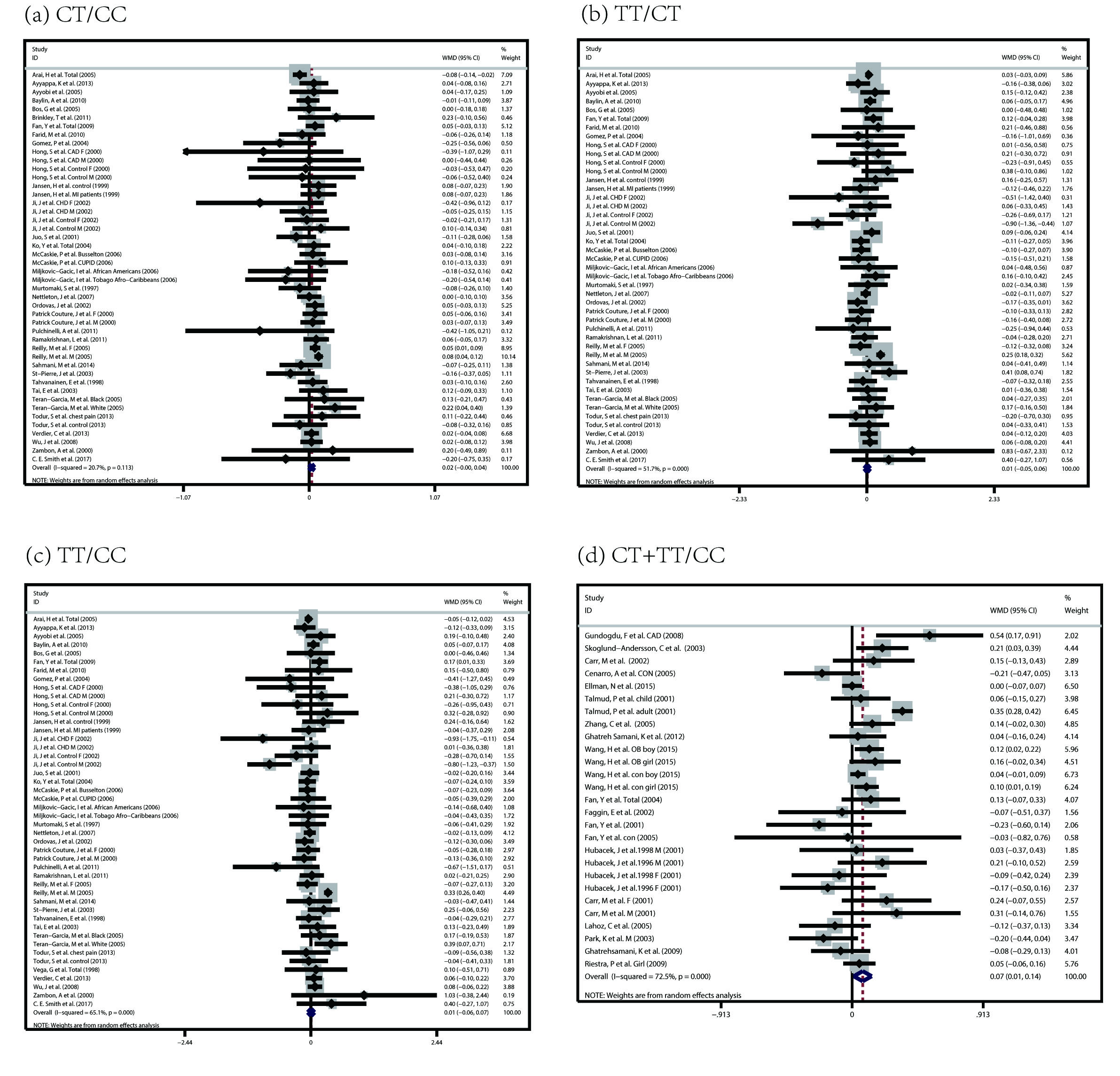


**TT/CC CT + TT/CC**

-.931 0 .931

-2.44 0 2.44

**Figure S2C** Forest plots of TC in C-514T


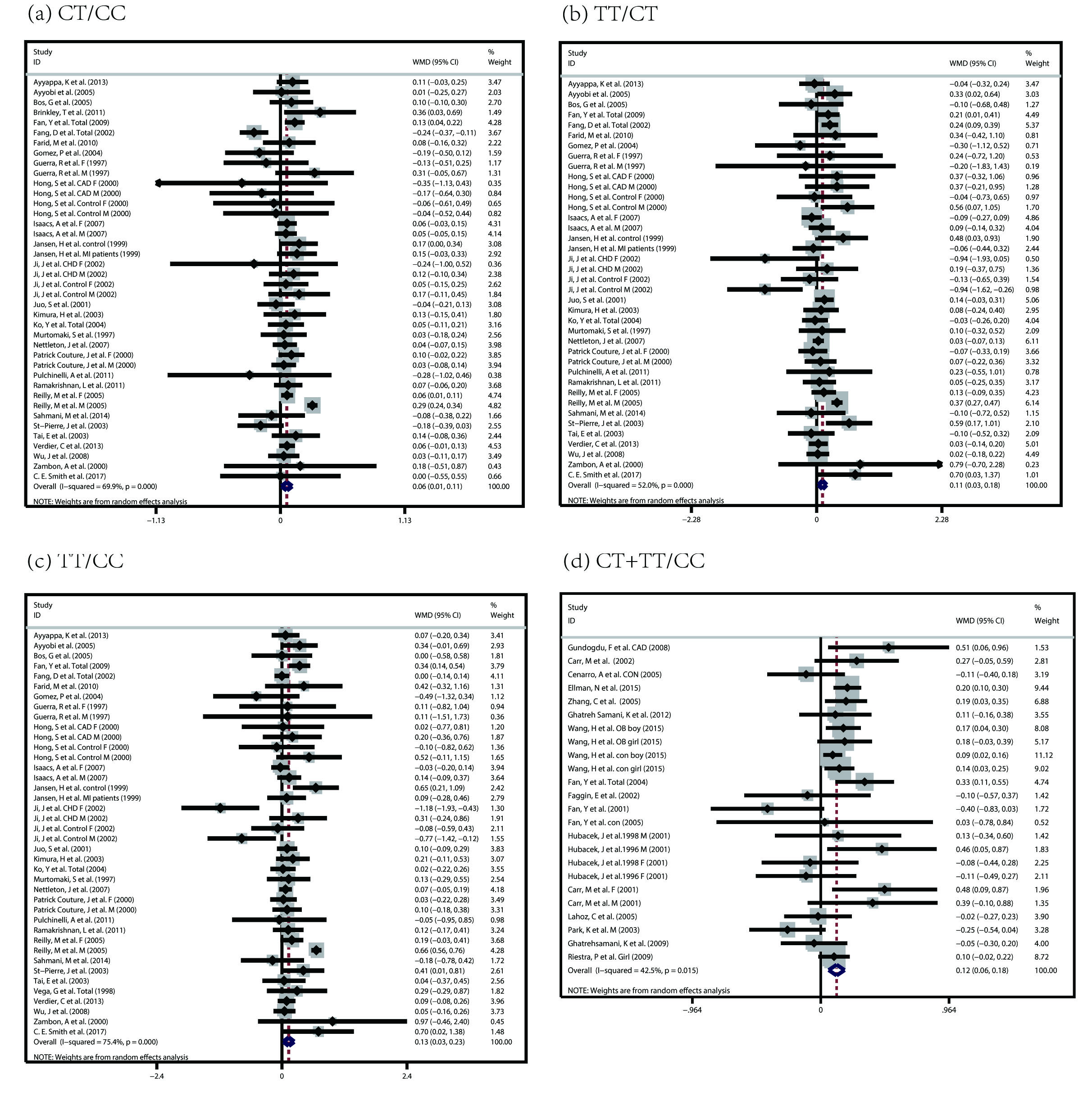


**CT/CC TT/CT**

-1.13 0 1.13 -2.28 0 2.28


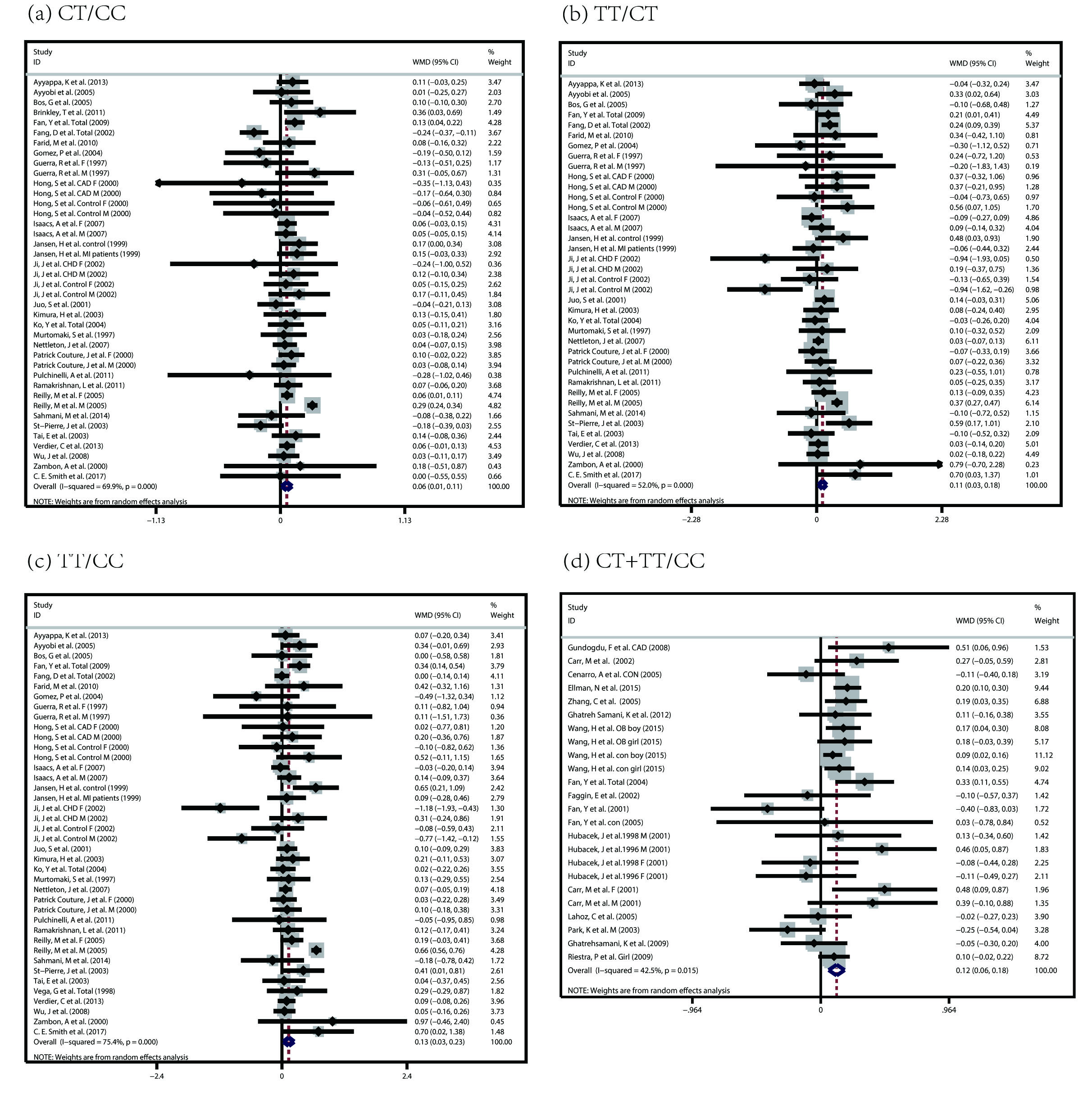


**TT/CC CT + TT/CC**


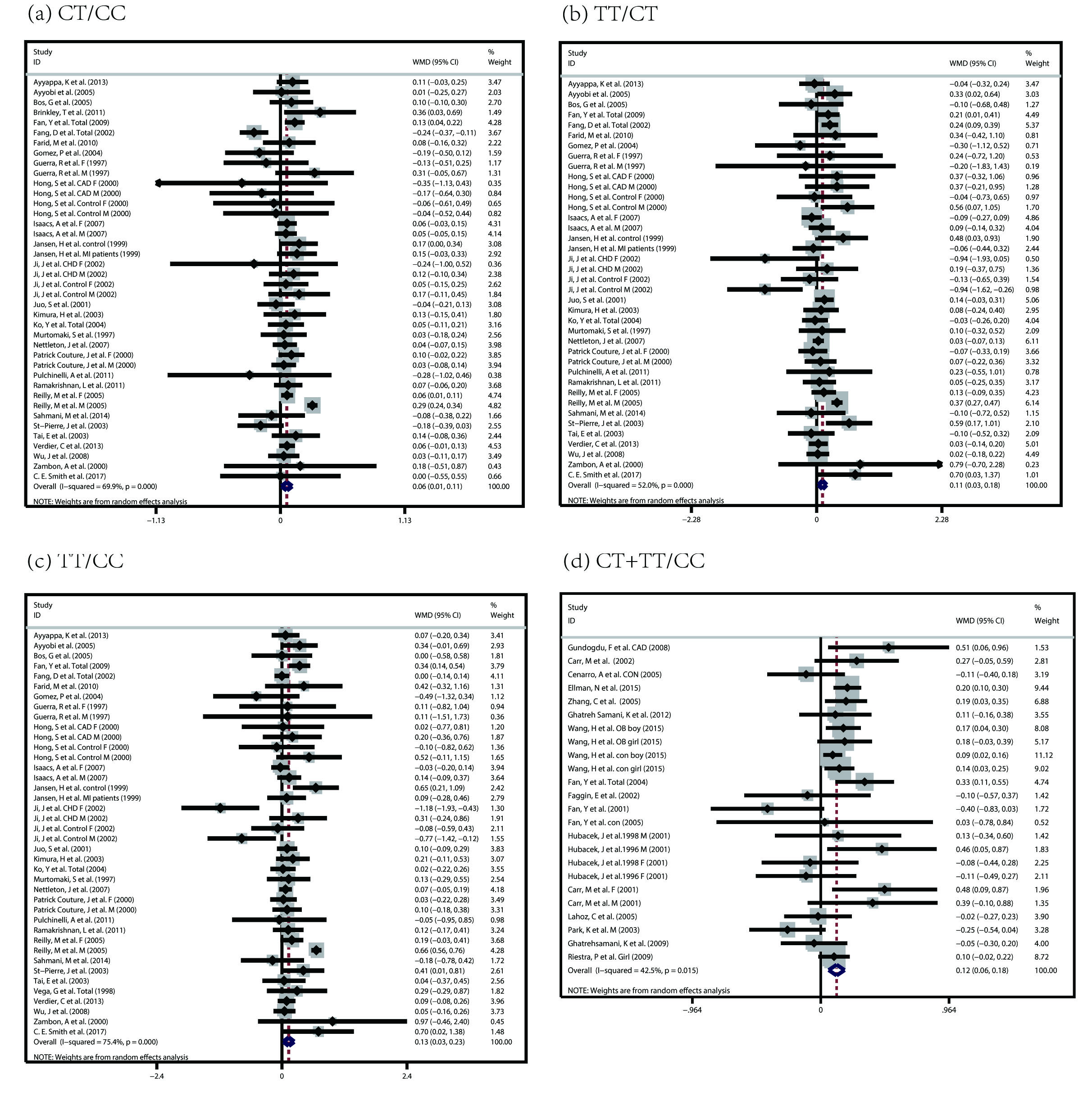


-.964 0 .964

-2.4 0 2.4

**Figure S2D** Forest plots of TG in C-514T

**CT/CC TT/CT**


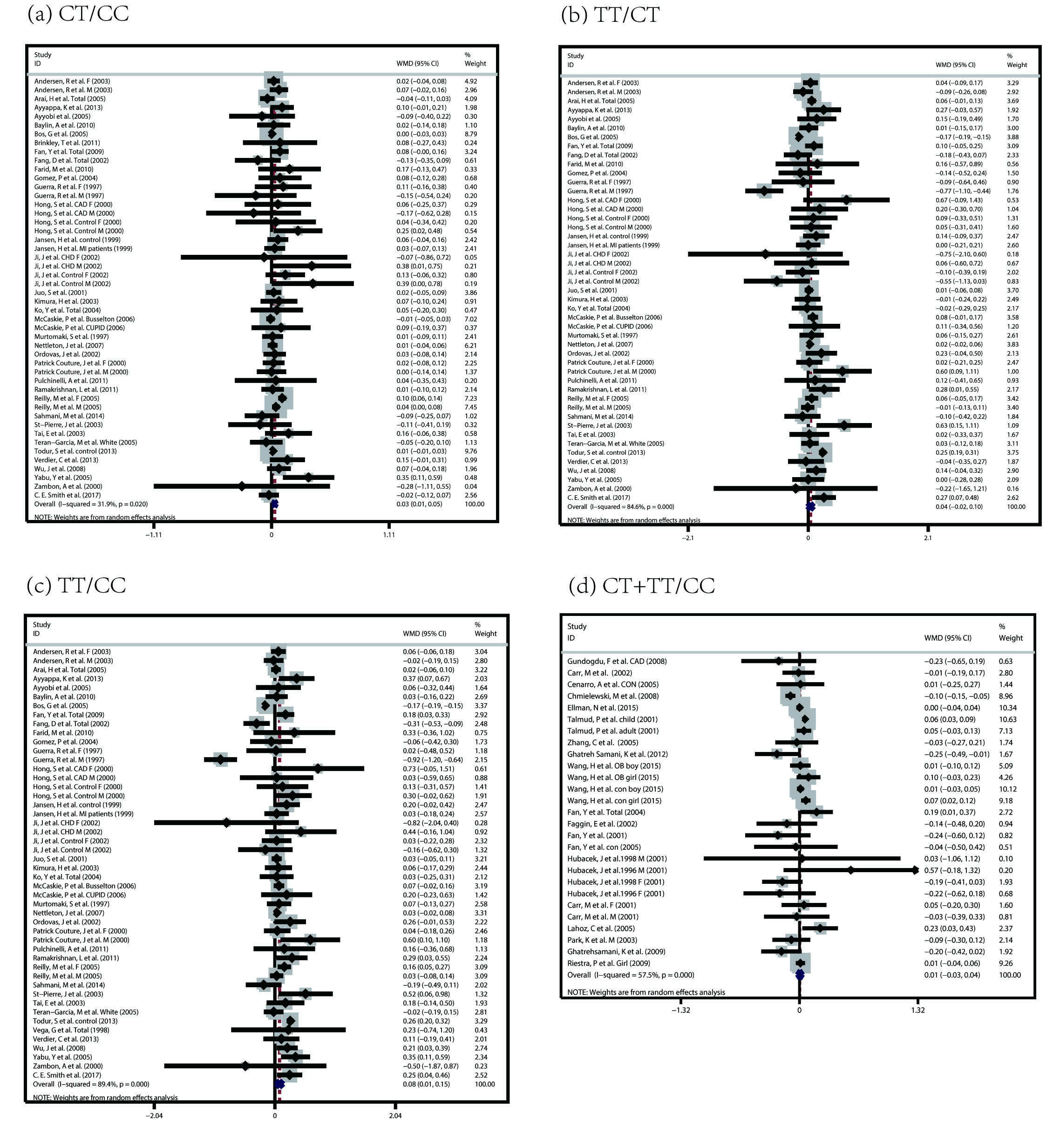

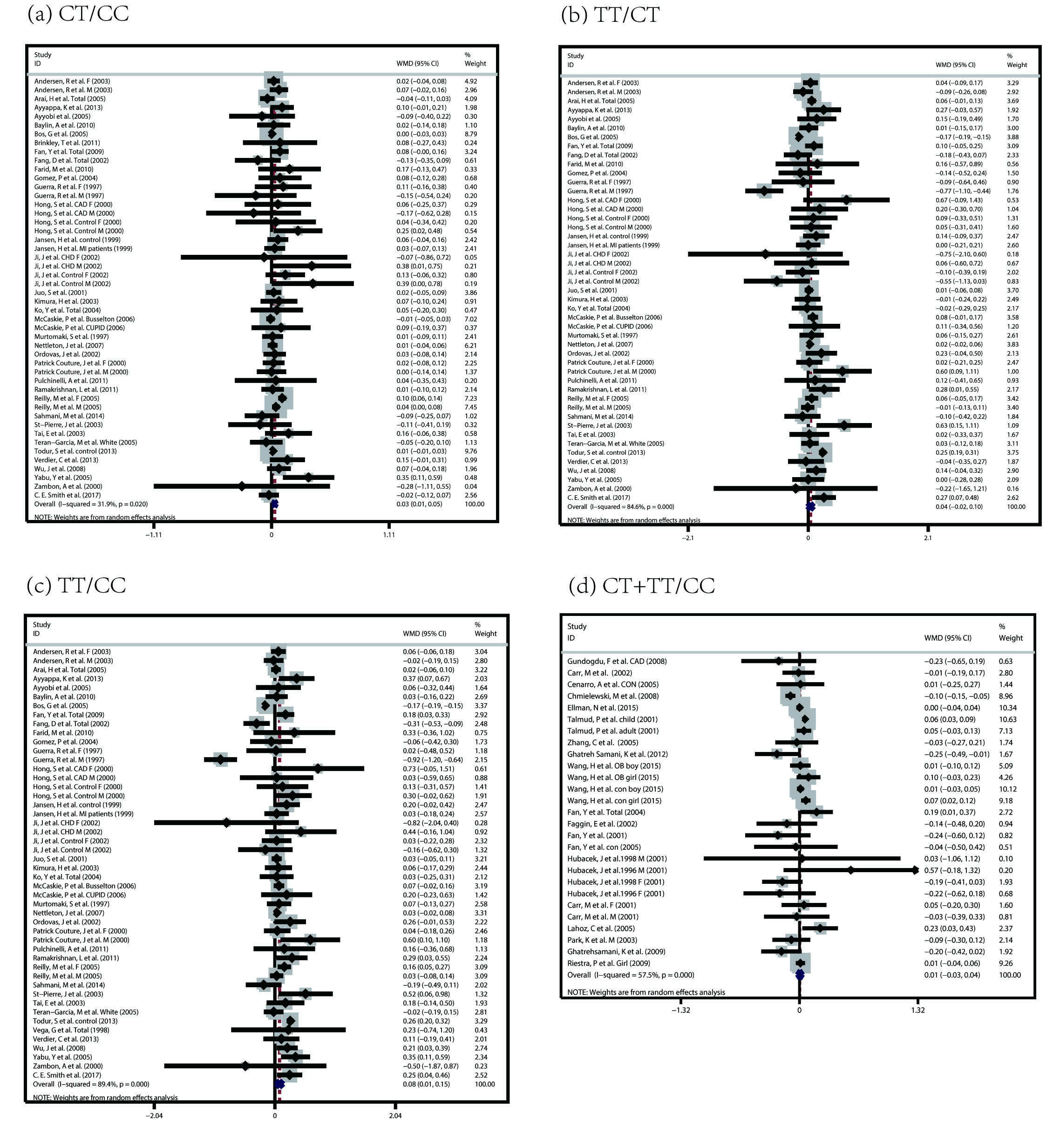

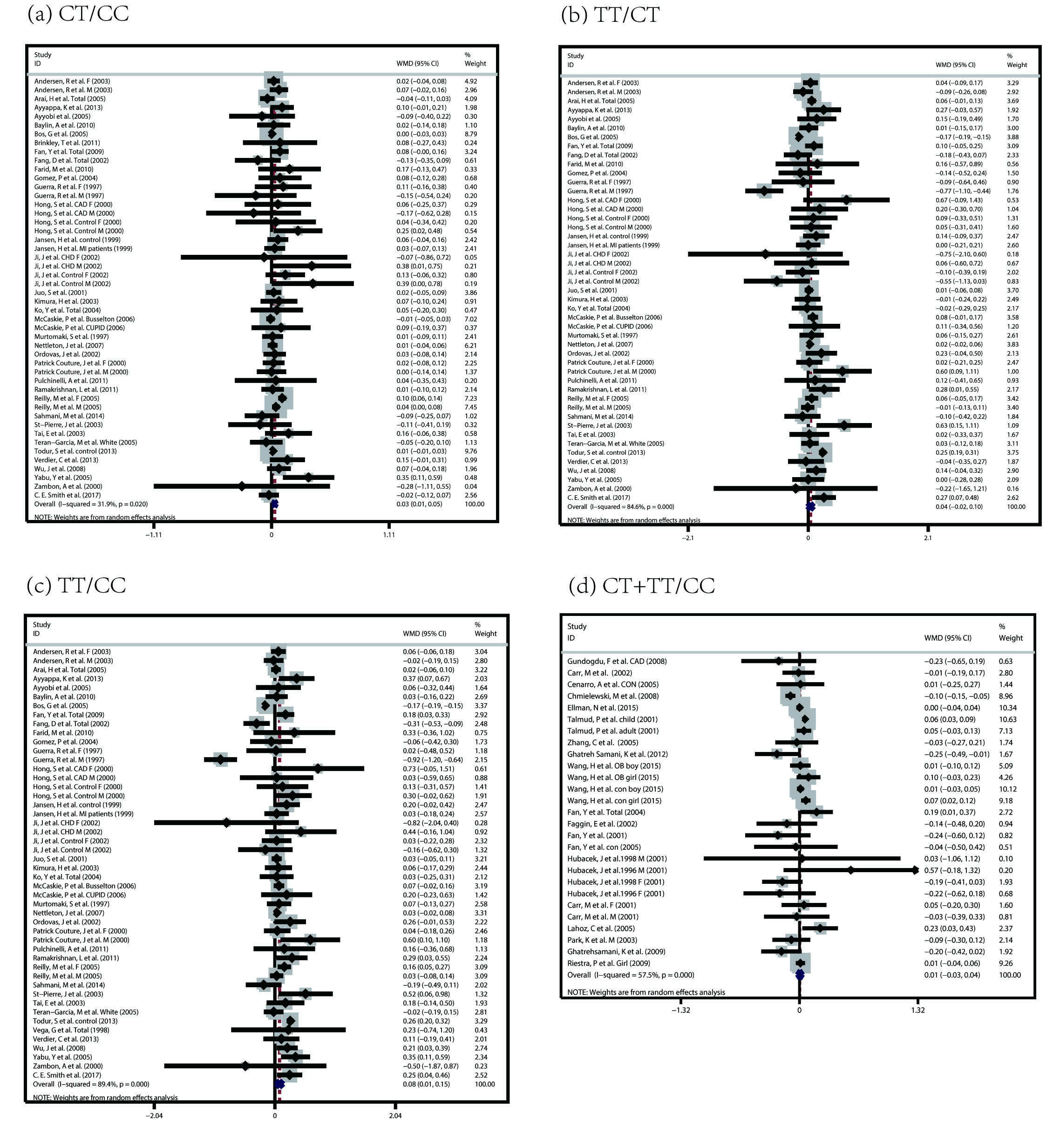


-1.32 0 1.32

-2.04 0 2.04

-1.11 0 1.11 -2.1 0 2.1

**TT/CC CT + TT/CC**

**Figure 2E** Forest plots of BMI in C-514T


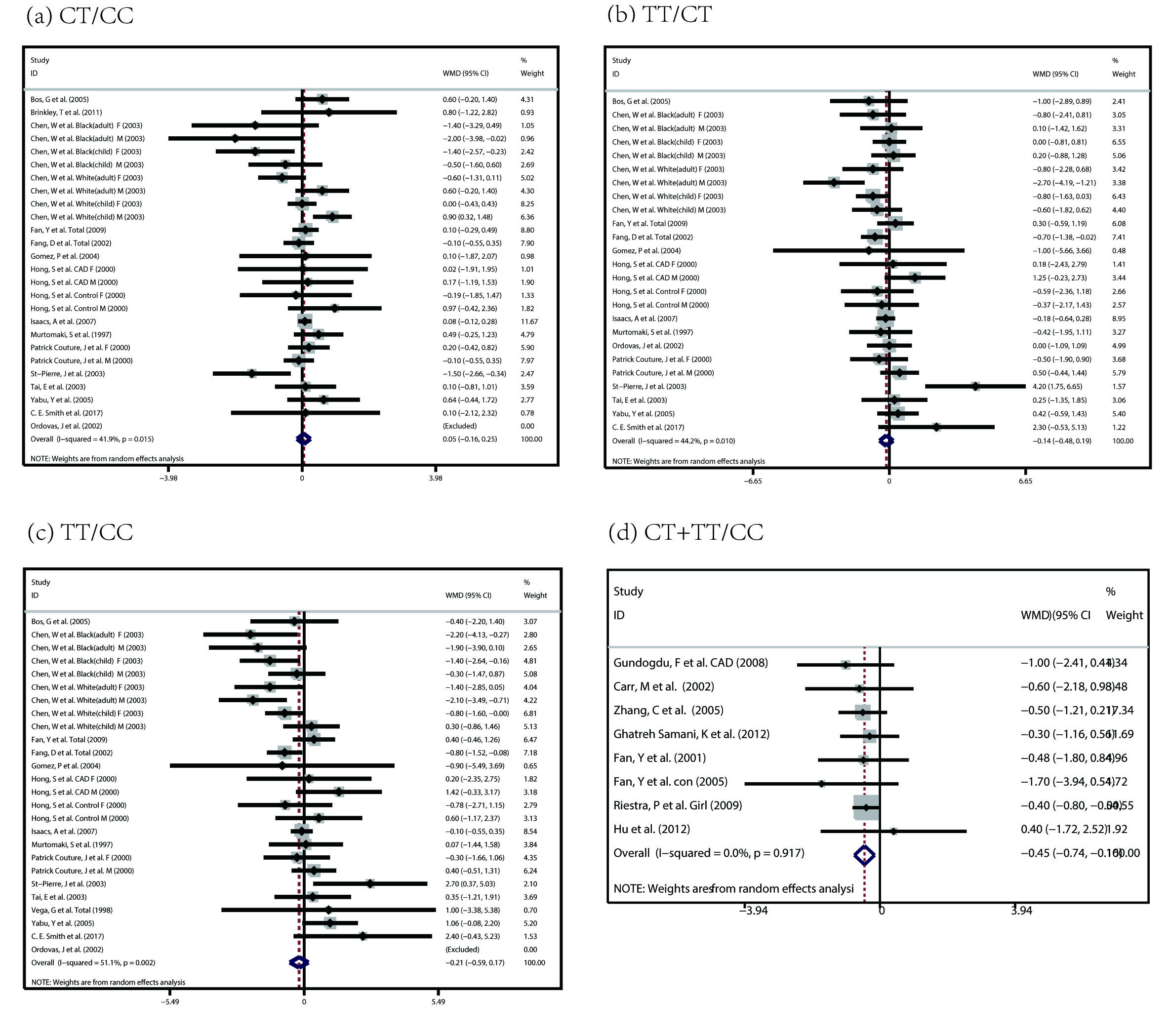


**CT/CC TT/CT**

-3.98 0 3.98 -6.65 0 6.65


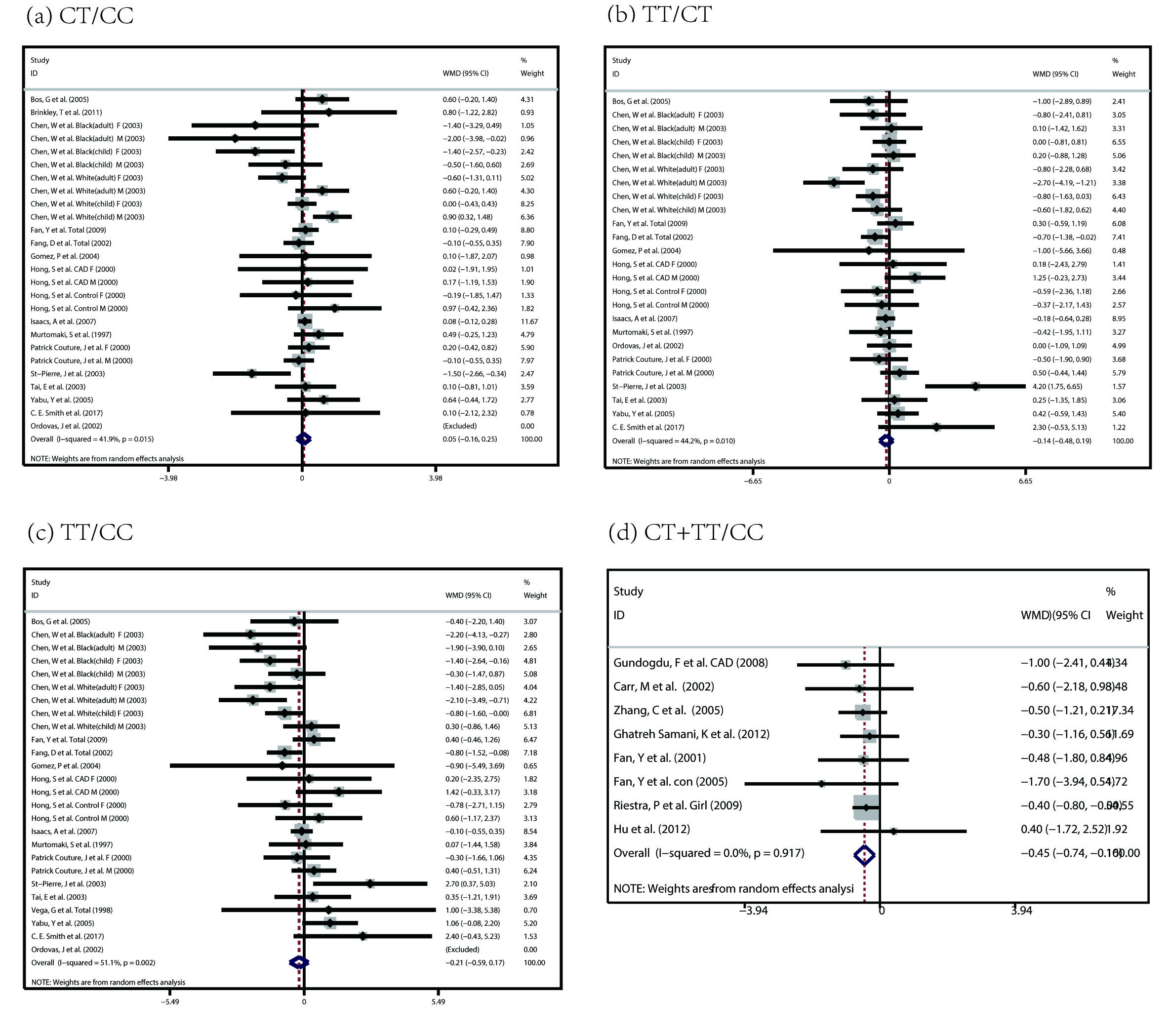

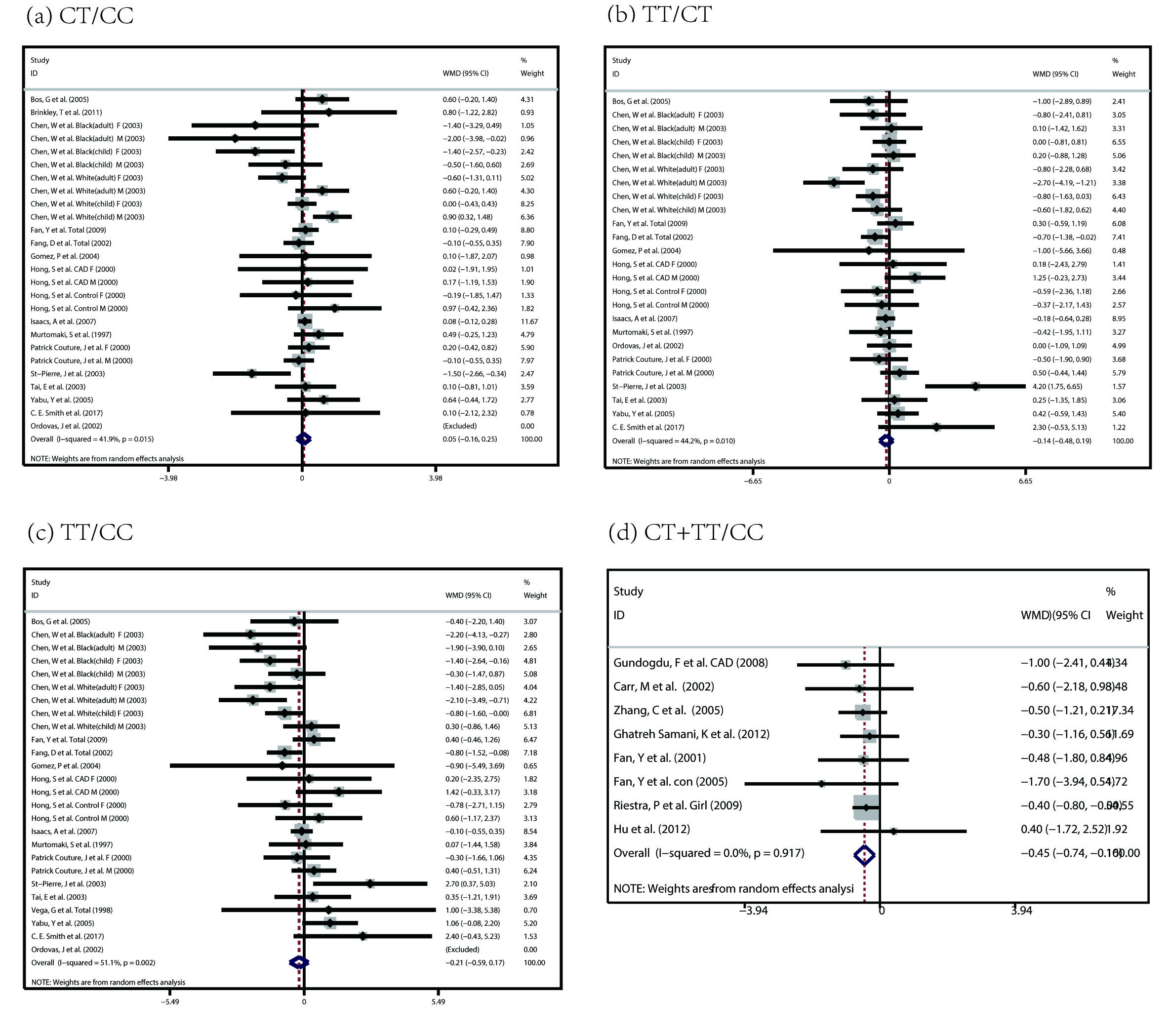


**TT/CC CT + TT/CC**

-3.94 0 3.94

-5.49 0 5.49

**Figure S2F** Forest plots of LIPC activity in C-514T


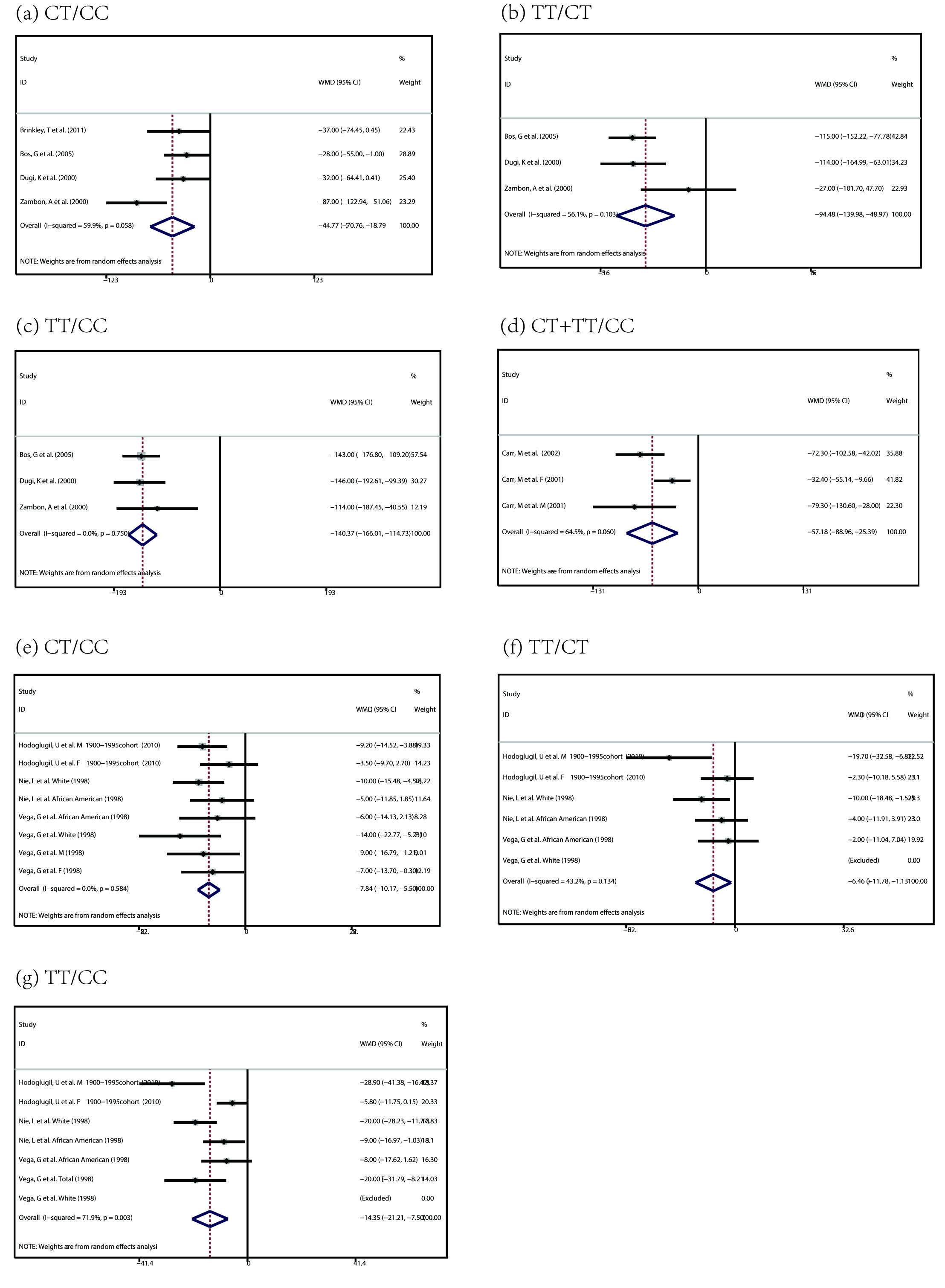

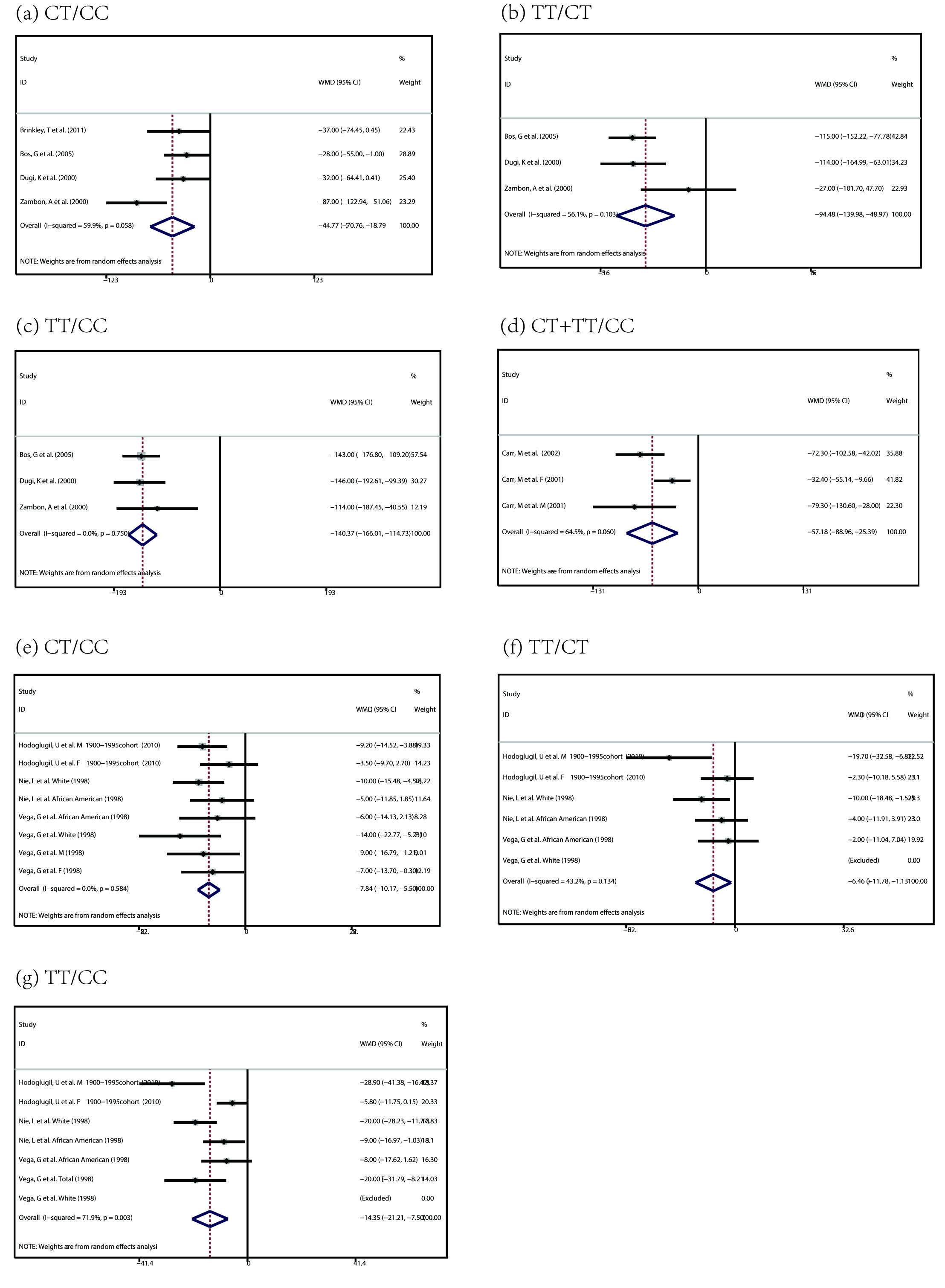


**CT/CC TT/CT**


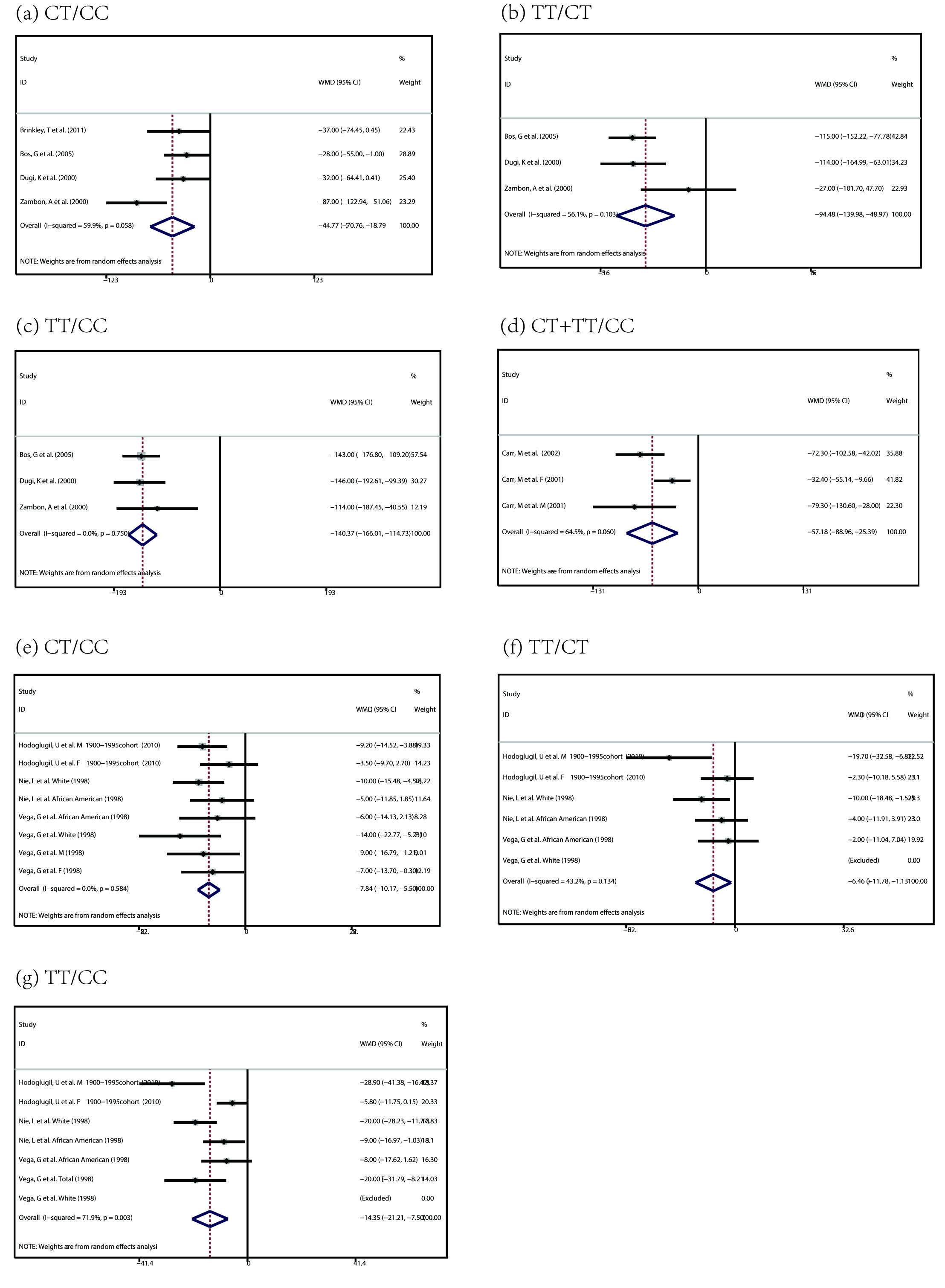

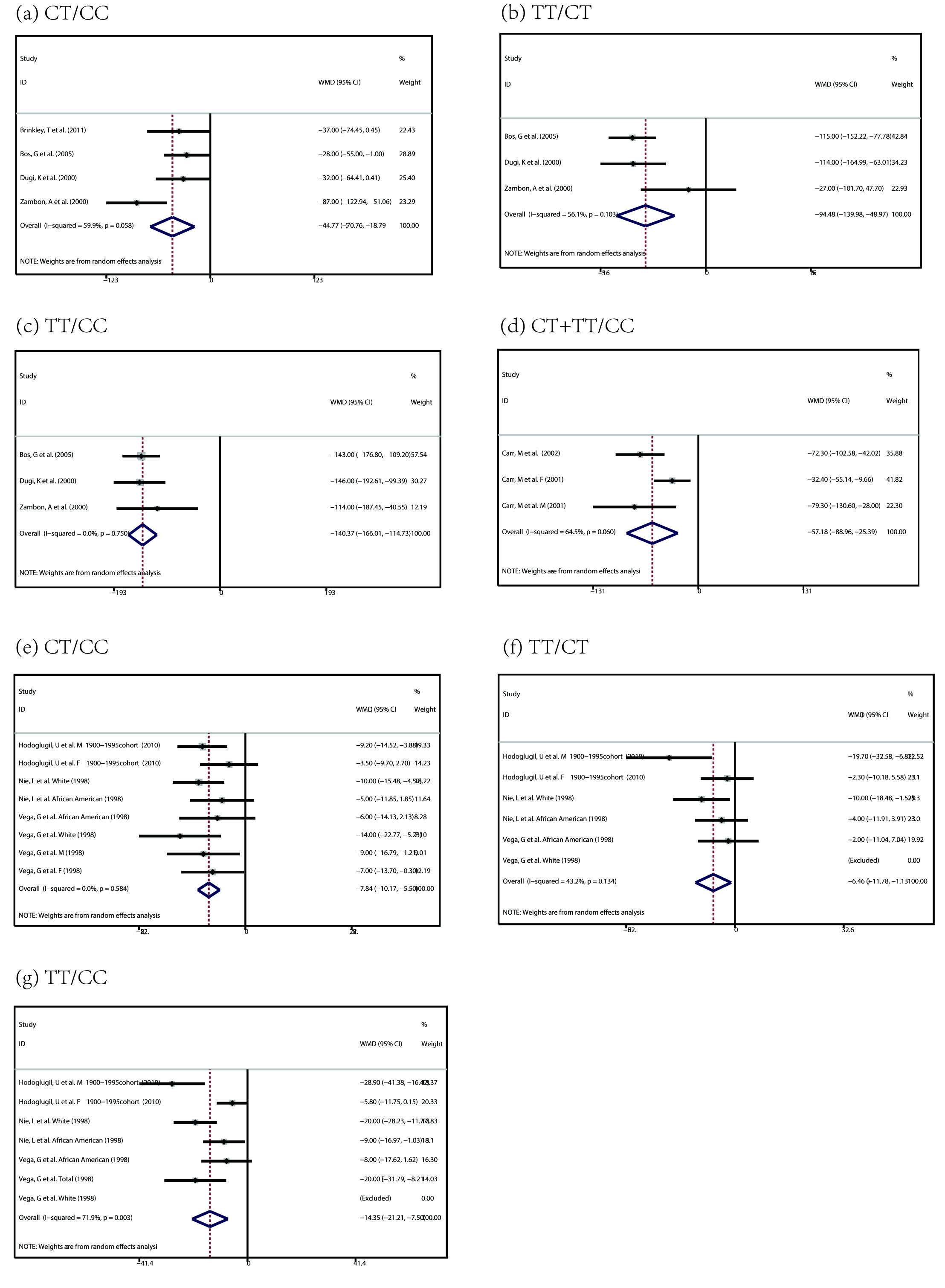


**TT/CC CT + TT/CC**

-123 0 123 -56 0 56


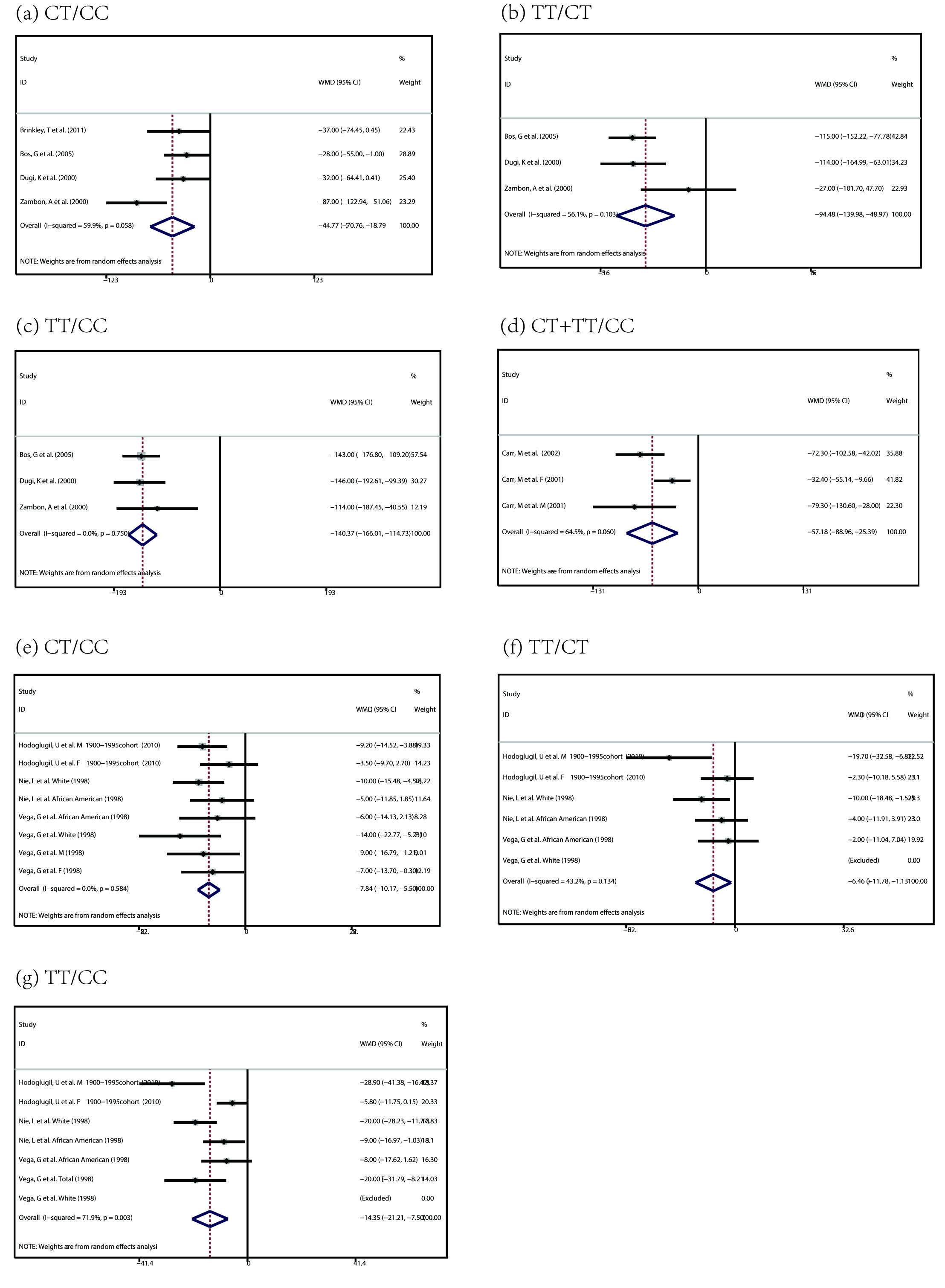

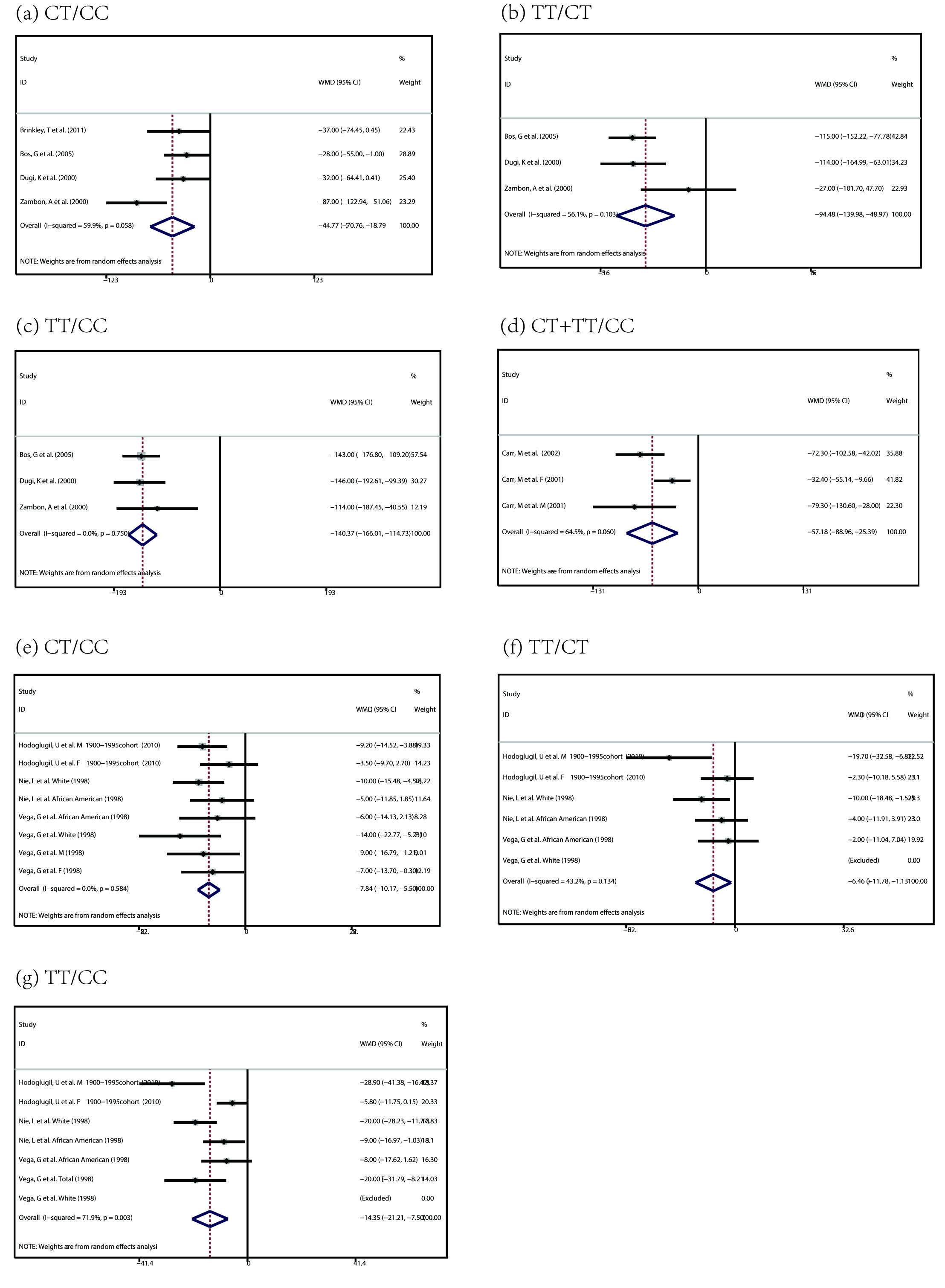


**CT/CC TT/CT**

-193 0 193 -131 0 131

-28 0 28 -32.6 0 32.6


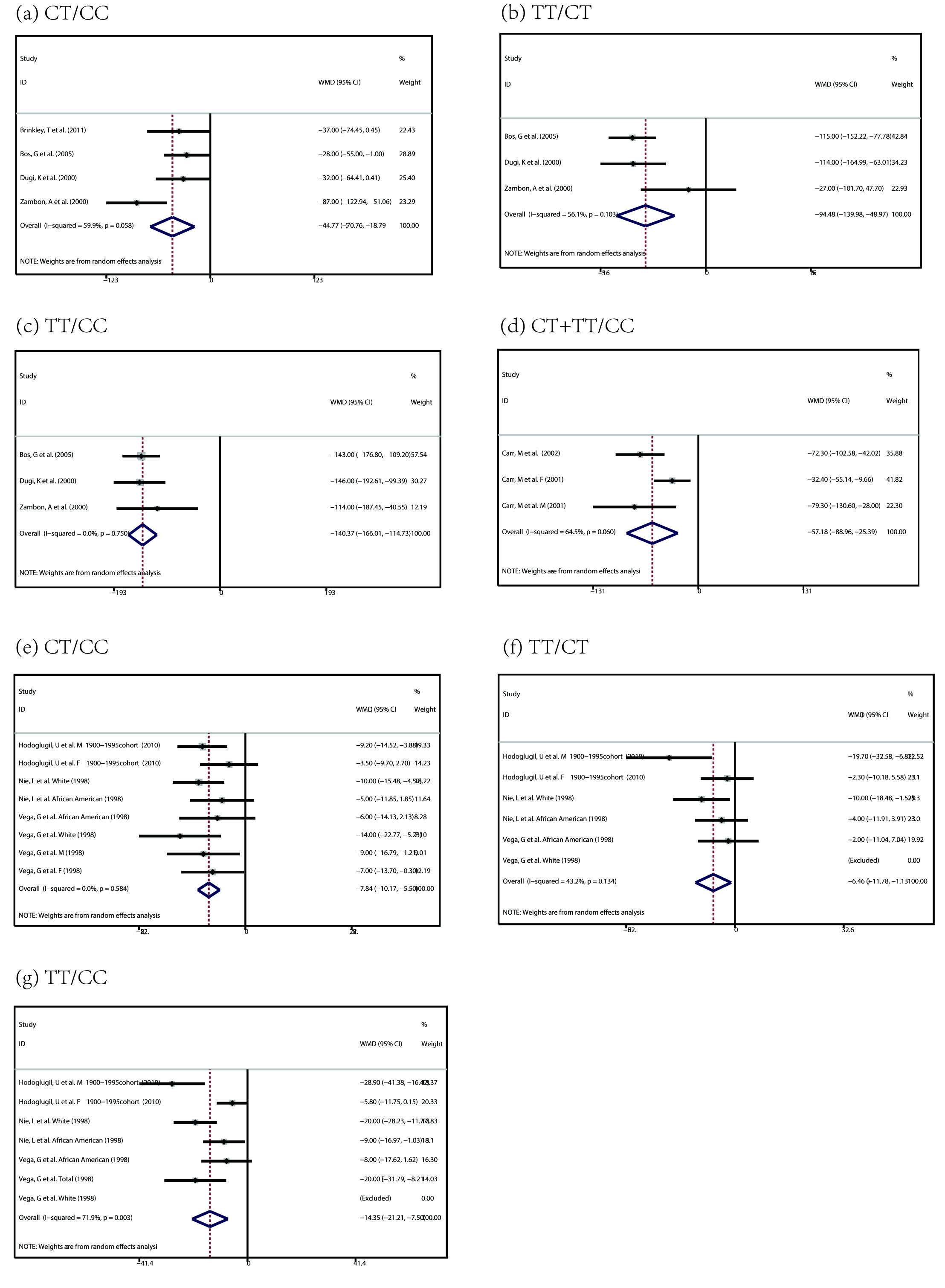


**TT/CC**

-41.4 0 41.4

**
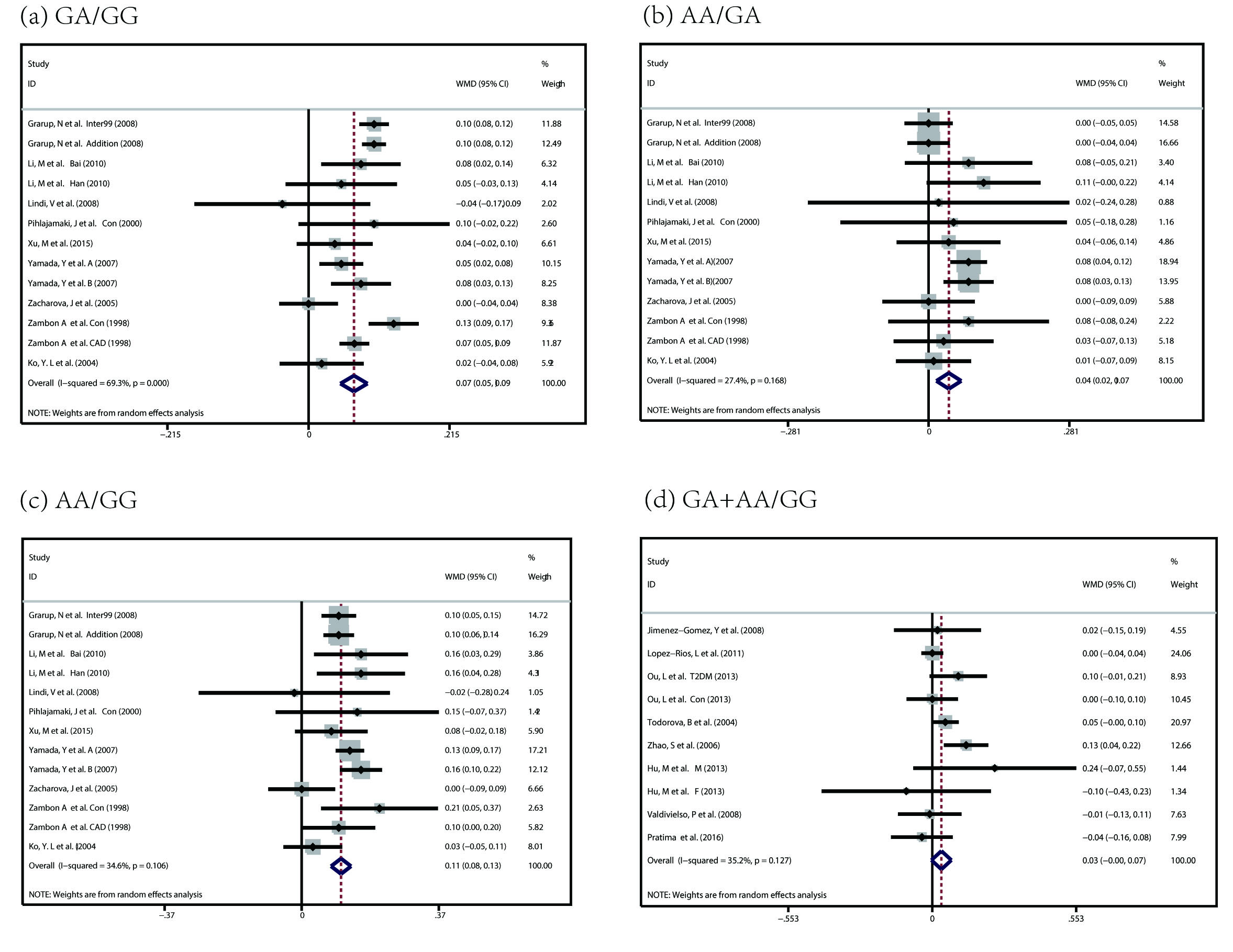
Figure S3A** Forest plots of HDL-c in G-250A

**GA/GG**

**AA/GA**

**
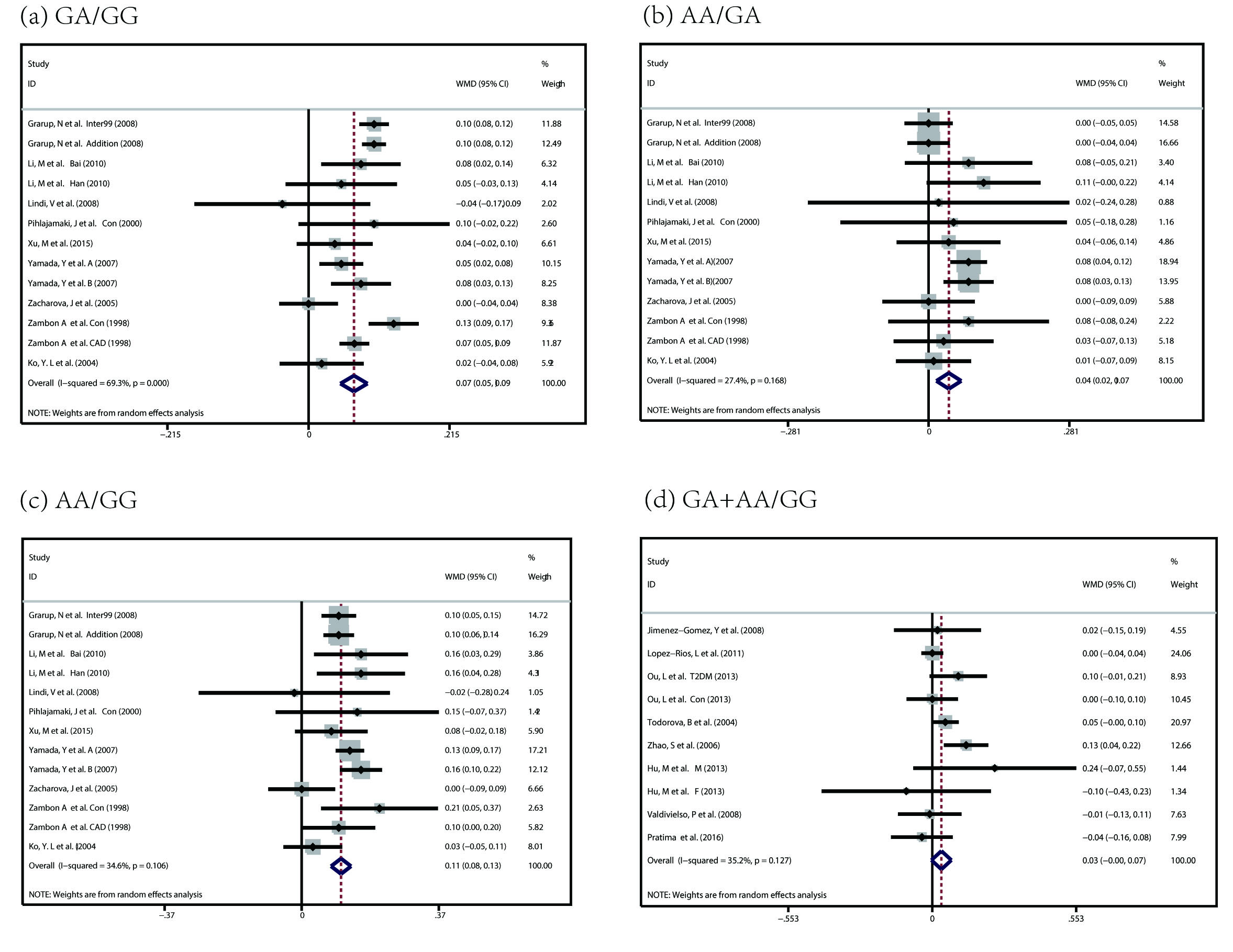

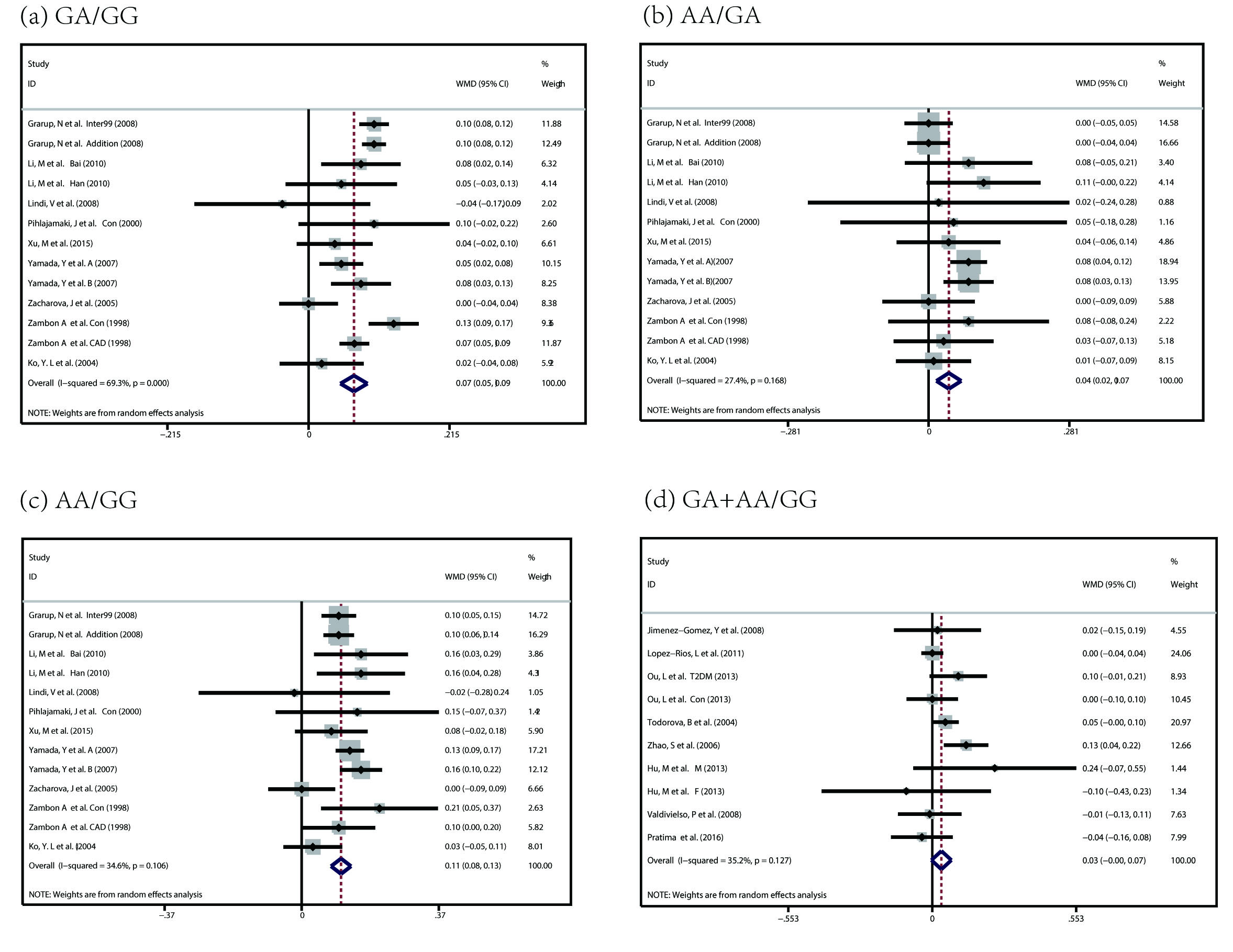
**

**GA + AA/GG**

**AA/GG**

-.281 0 .281

-.215 0 .215

-.533 0 .533

-.37 0 .37

**Figure S3B** Forest plots of LDL-c in G-250A


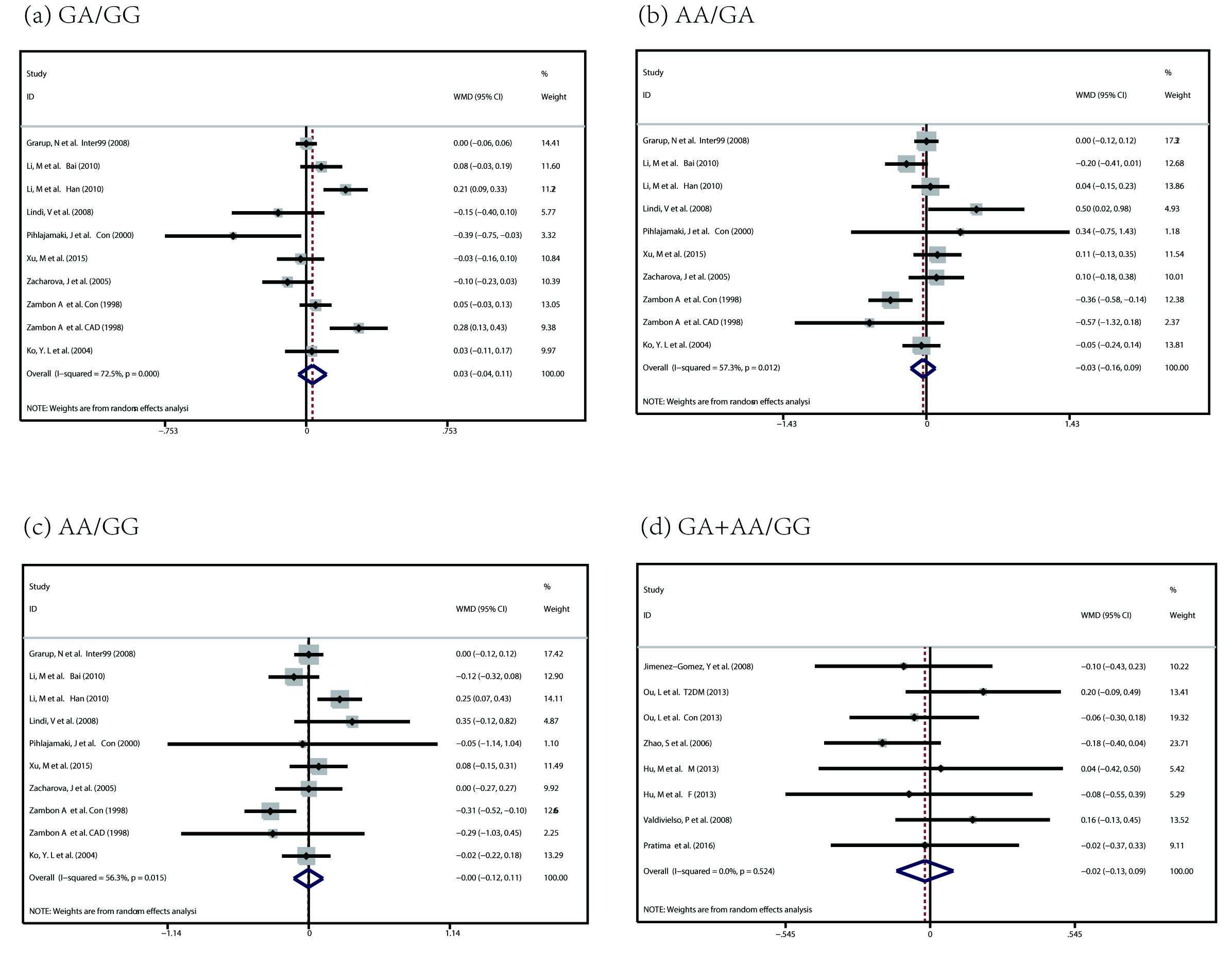

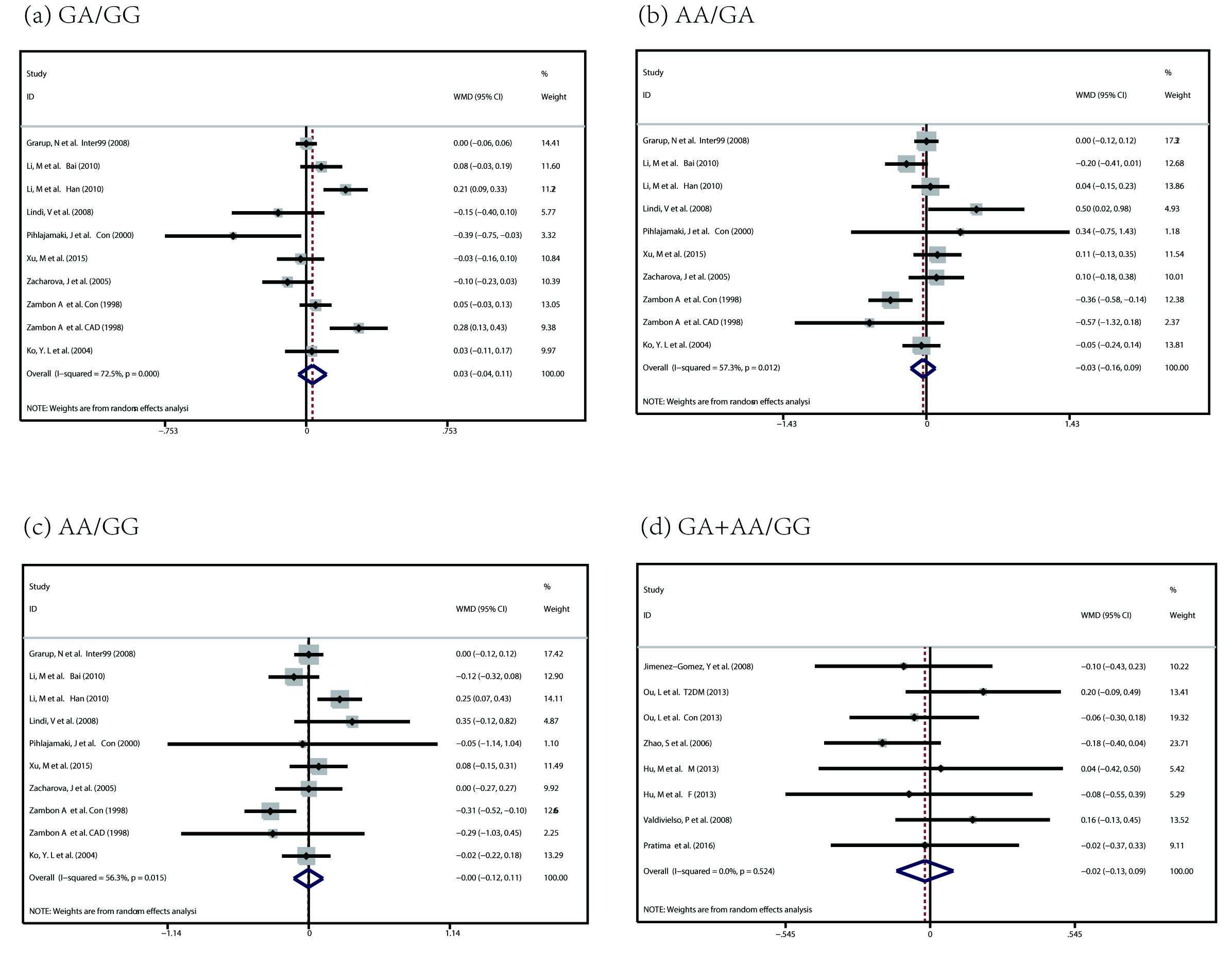


**AA/GA**

**GA/GG**


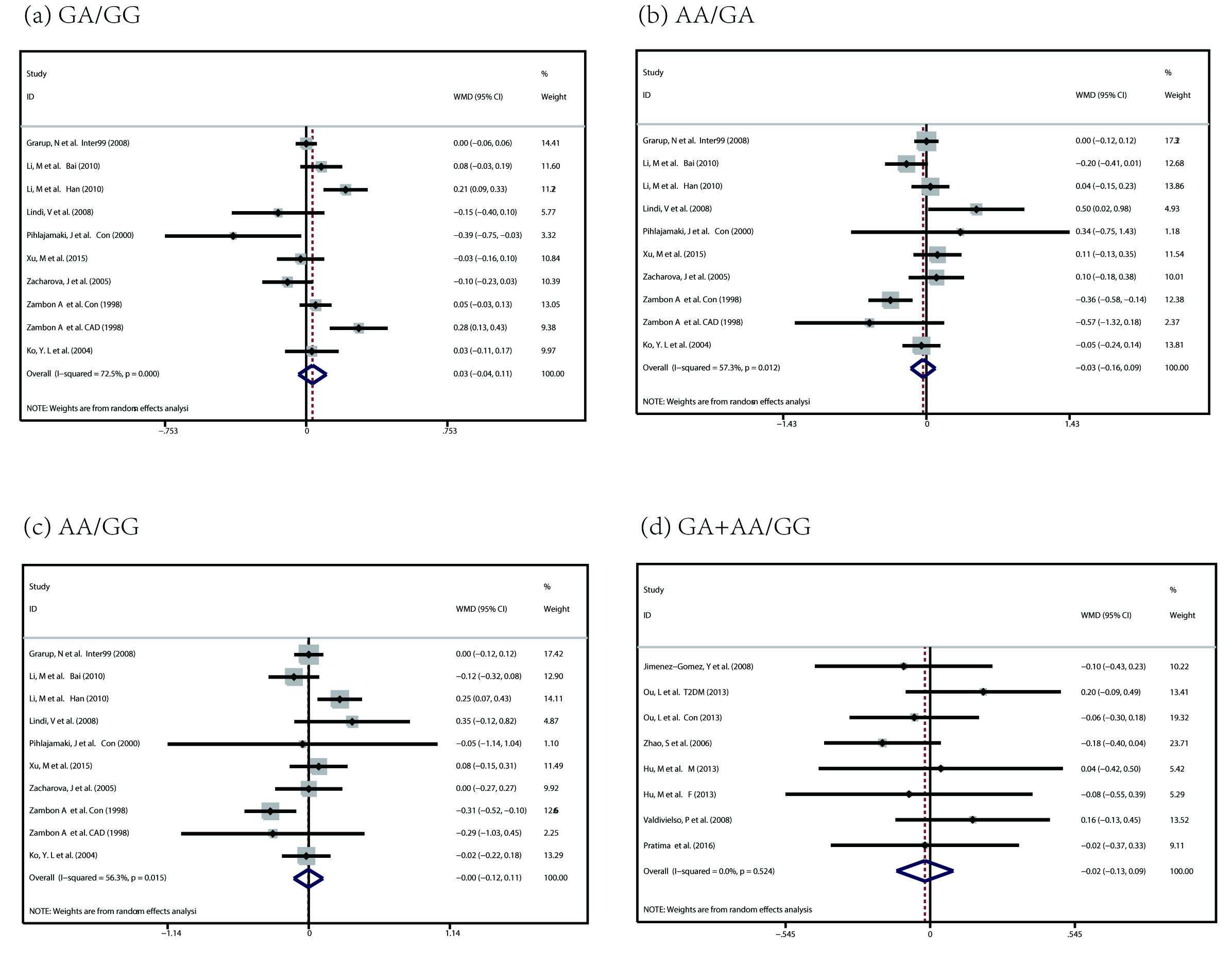


-1.43 0 1.43

-.753 0 .753

**GA + AA/GG**

**AA/GG**


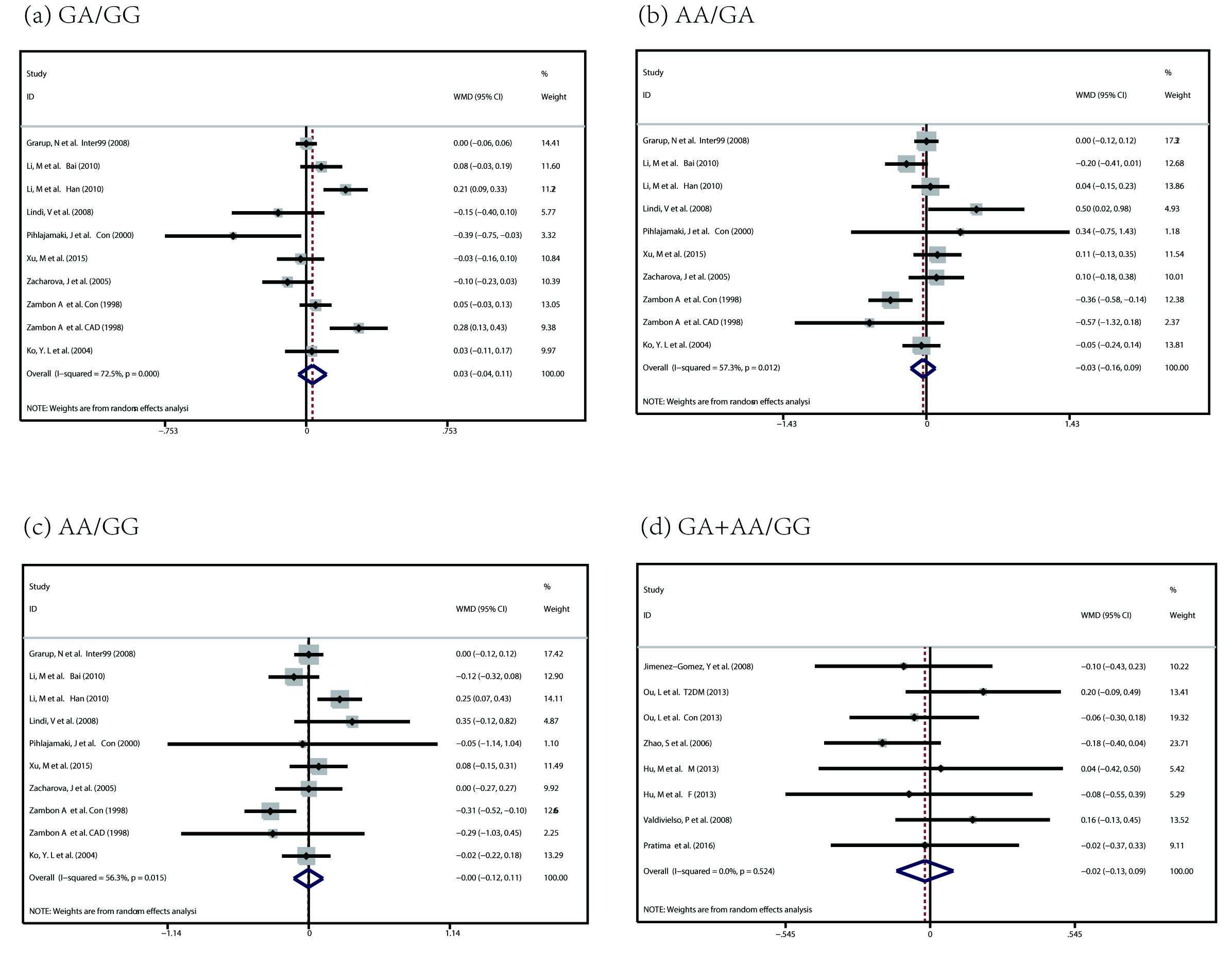


-.545 0 .545

-1.14 0 1.14

**Figure S3C** Forest plots of TC in G-250A


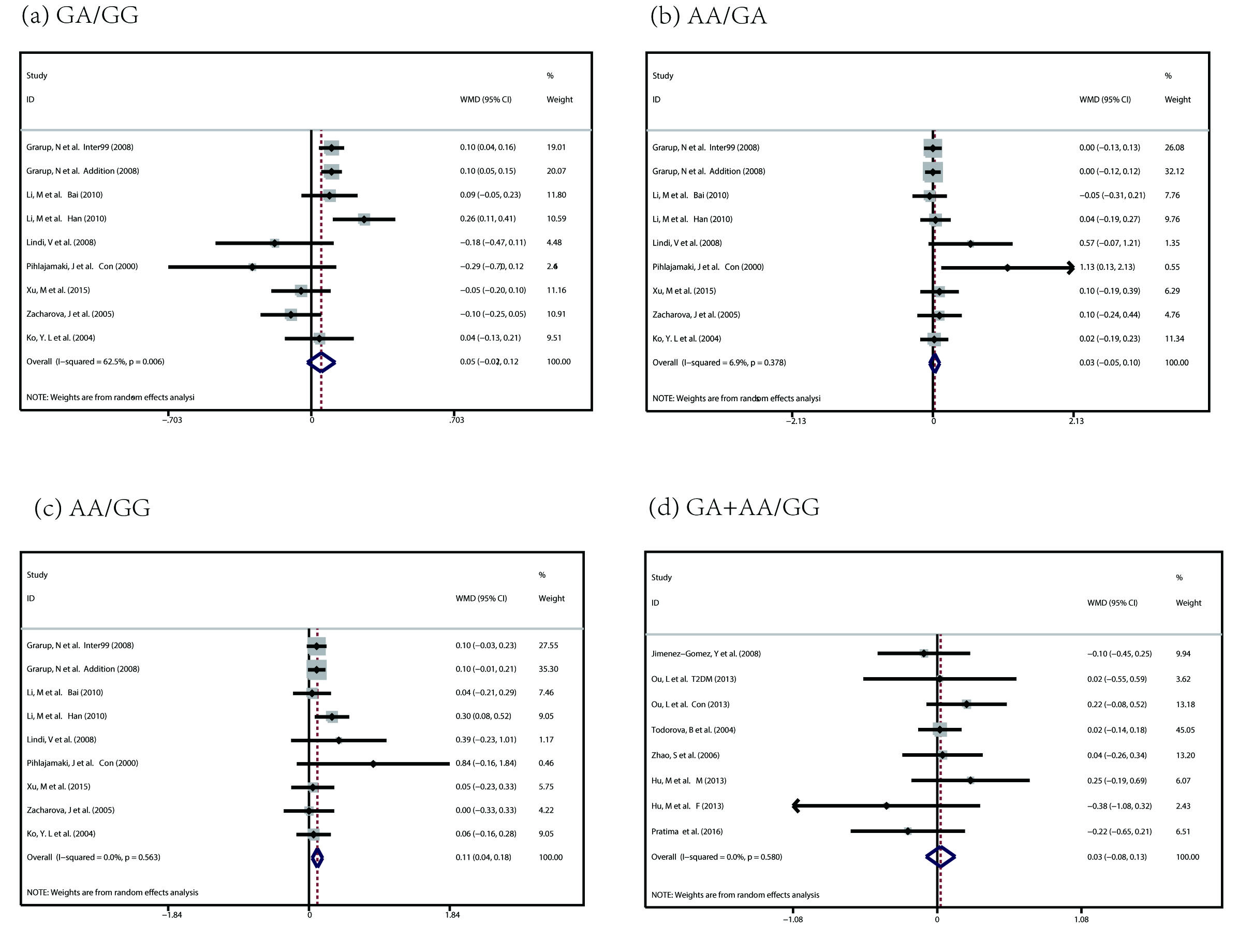


**AA/GA**

**GA/GG**


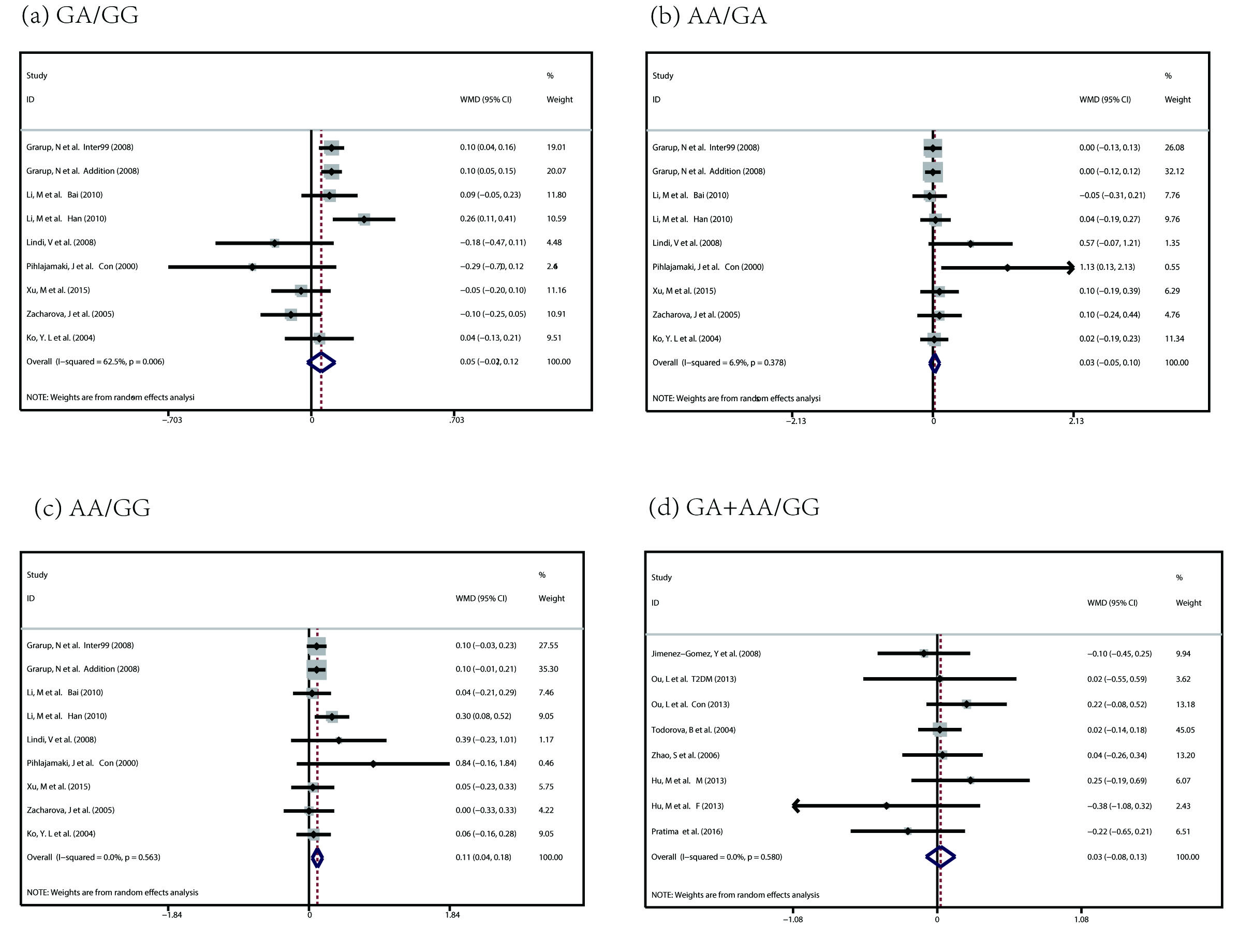


**GA + AA/GG**

**AA/GG**

-2.13 0 2.13

-.703 0 .703

-1.08 0 1.08

-1.84 0 1.84

**
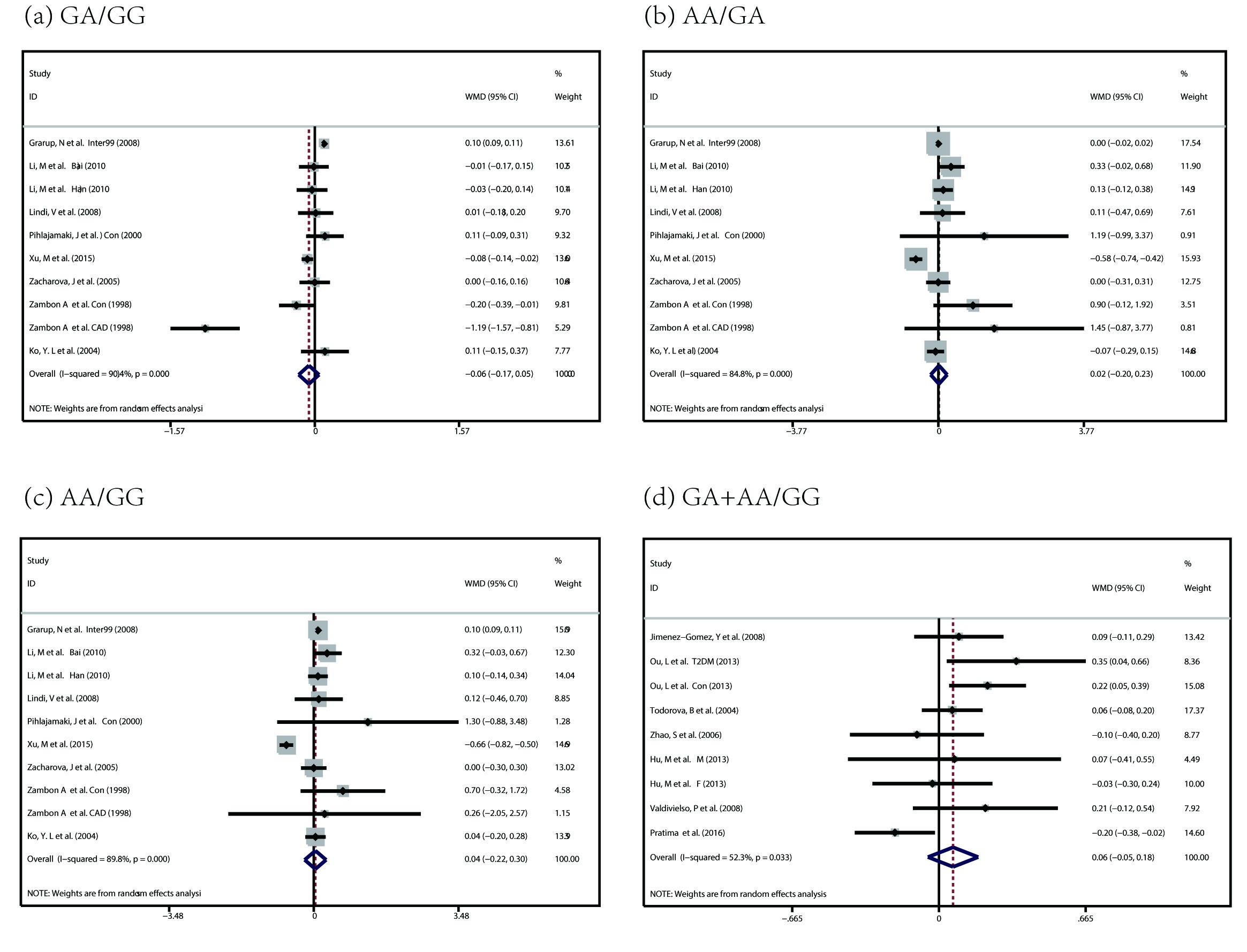
Figure S3D** Forest plots of TG in G-250A

**AA/GA**

**GA/GG**


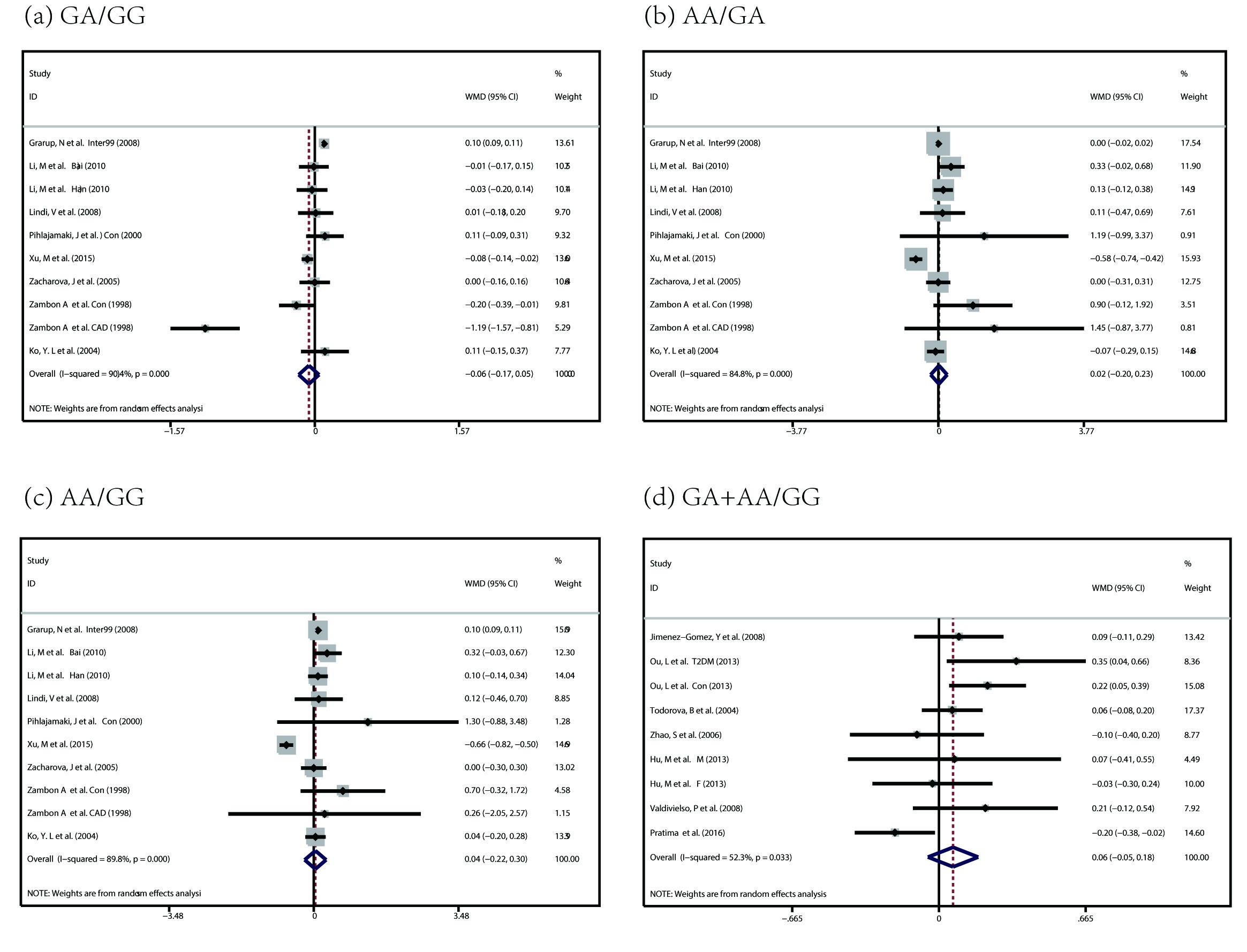


**GA + AA/GG**

**AA/GG**

-3.77 0 3.77

-1.57 0 1.57

-.655 0 .655

-3.48 0 3.48

**Figure S3E** Forest plots of BMI in G-250A


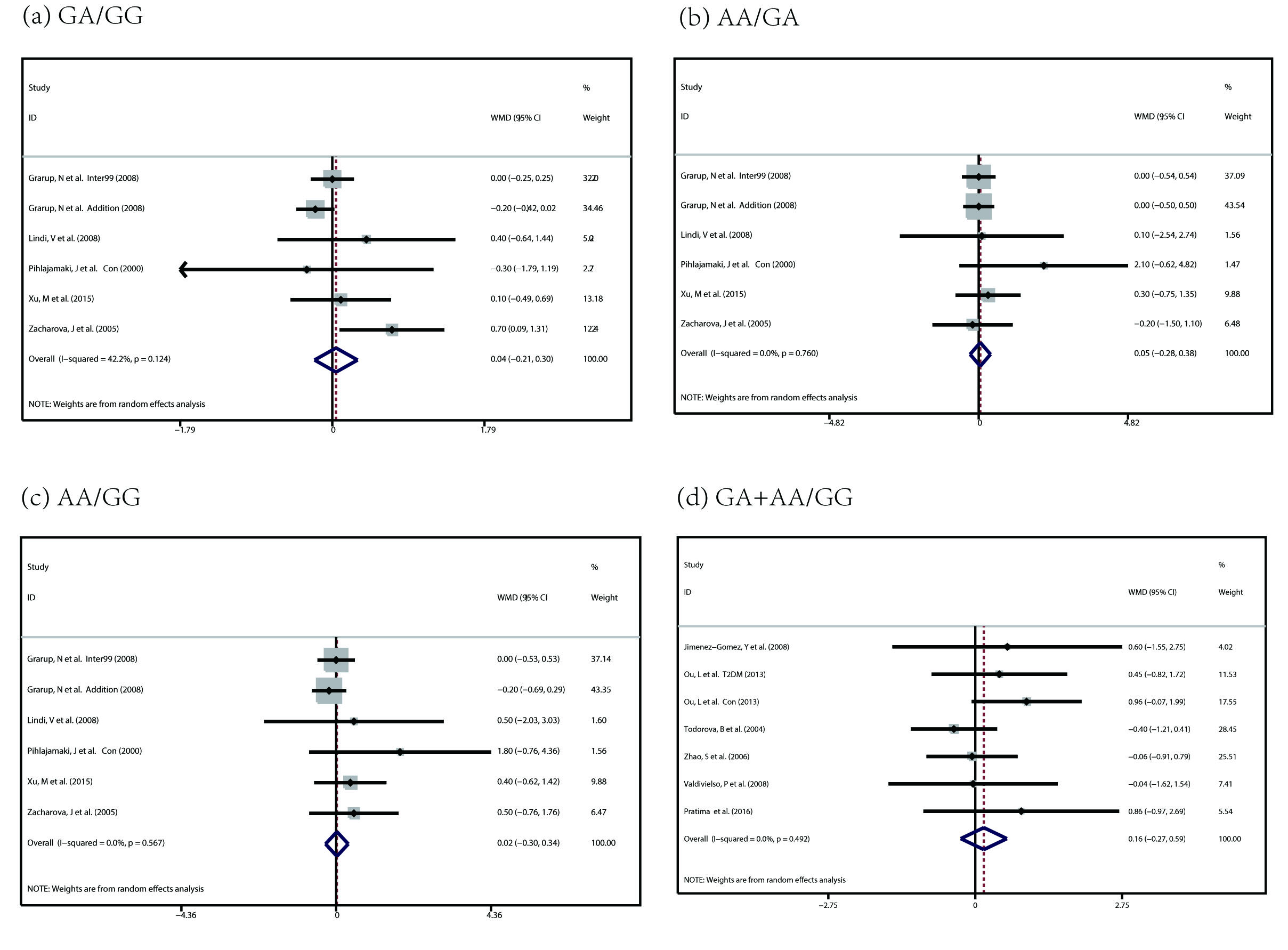

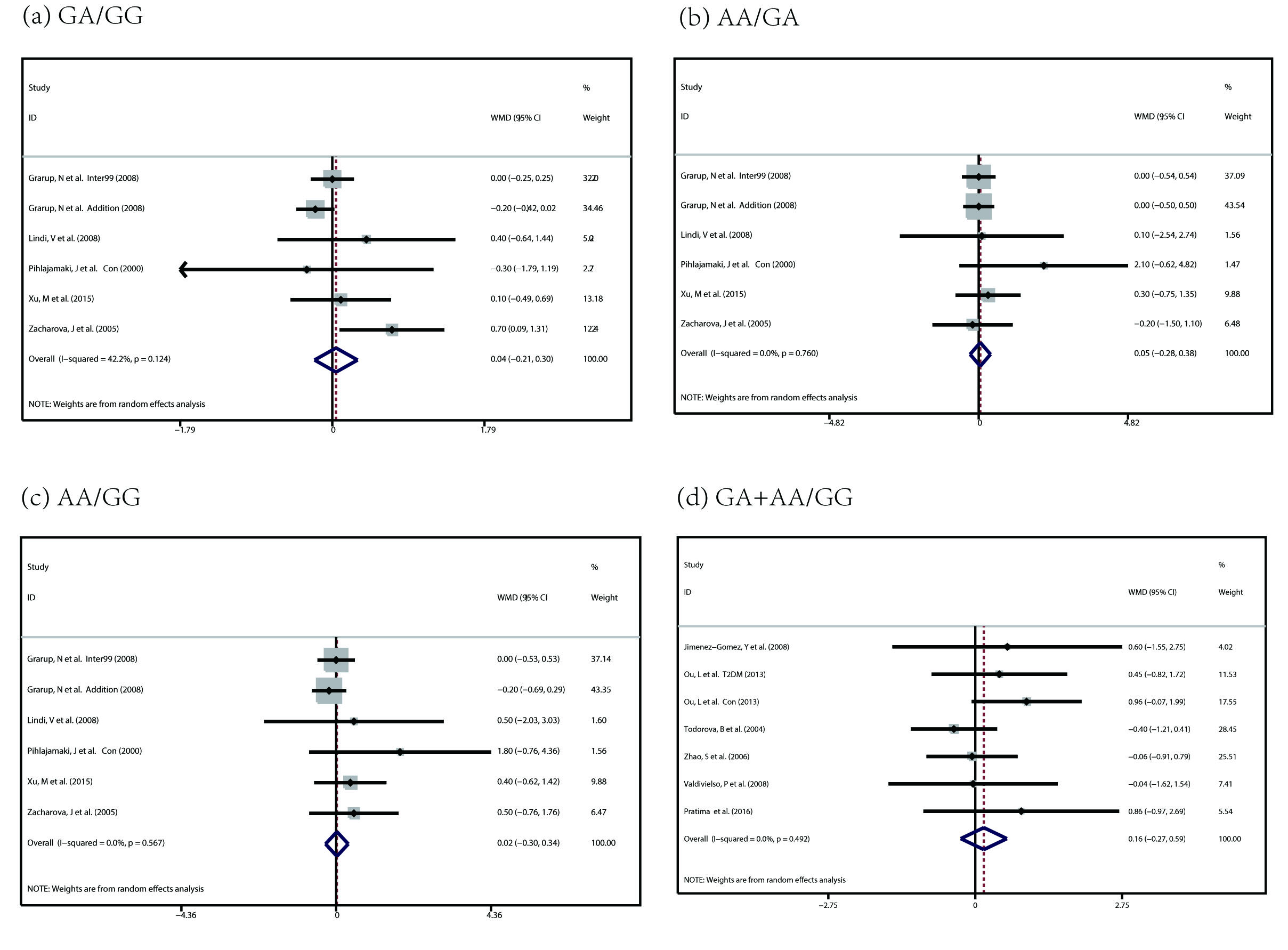


**AA/GA**

**GA/GG**


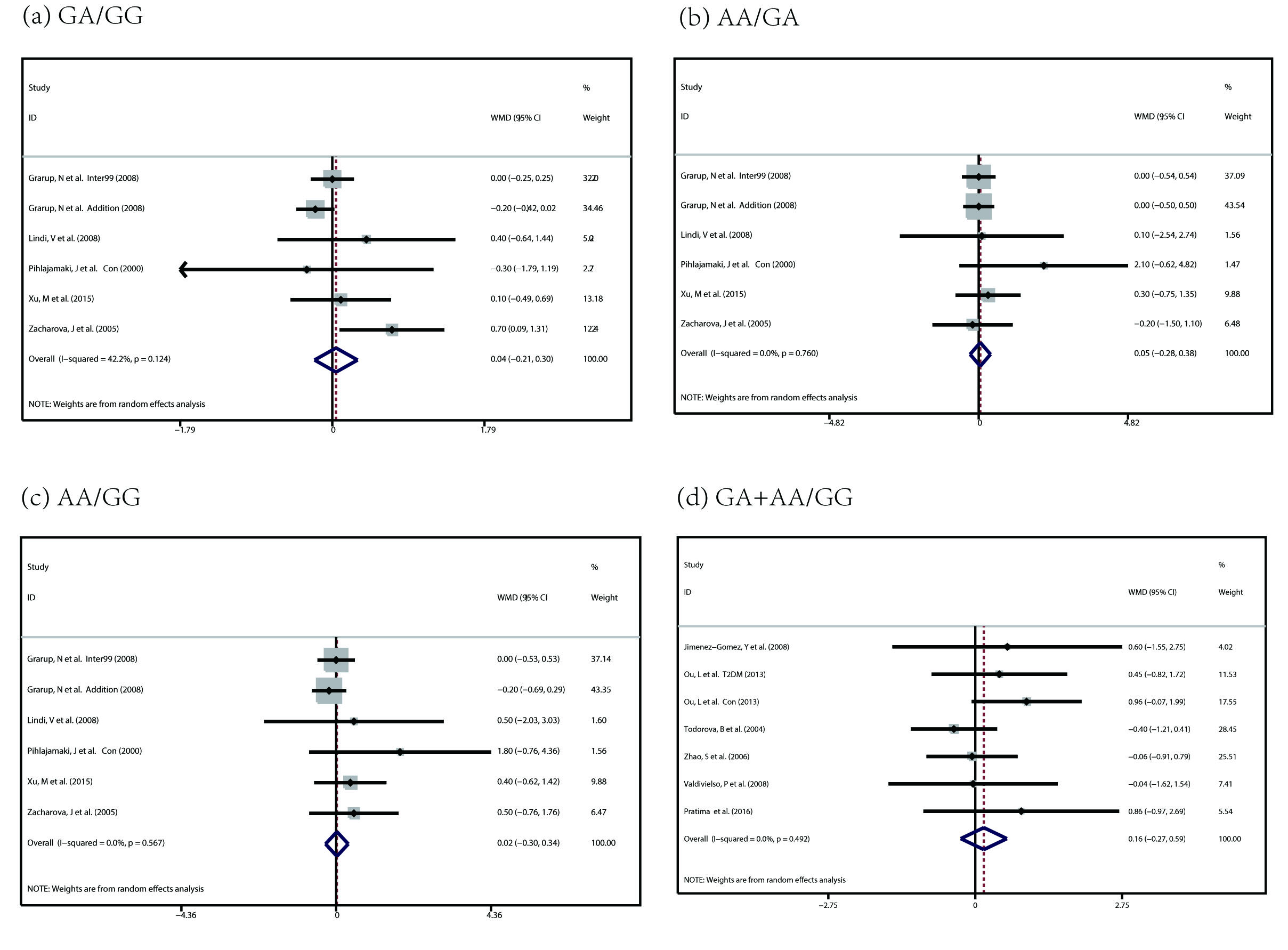


**AA/GG**

**GA + AA/GG**

-4.82 0 4.82

-1.79 0 1.79


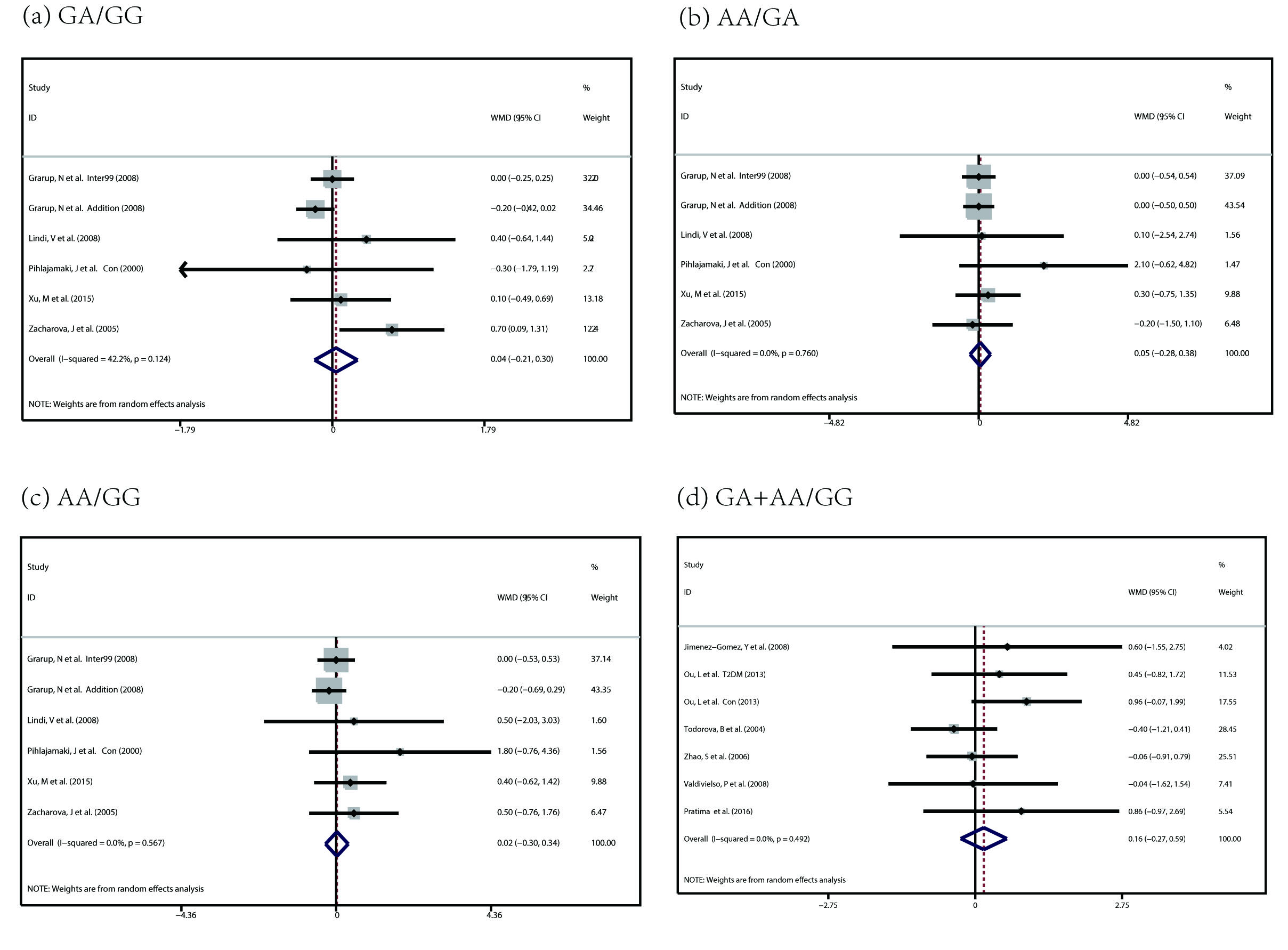


-2.75 0 2.75

-4.63 0 4.63
