# Supplementary material for: The cross‐sectional study of hepatic lipase SNPs and plasma lipid levels
Source: Food Sci Nutr. 2020 Jan 13;8(2):1162–72. doi: 10.1002/fsn3.1403 (PMC7180388; doi:10.1002/fsn3.1403)
Supplement: Supplementary file 2 [file FSN3-8-1162-s002.docx]

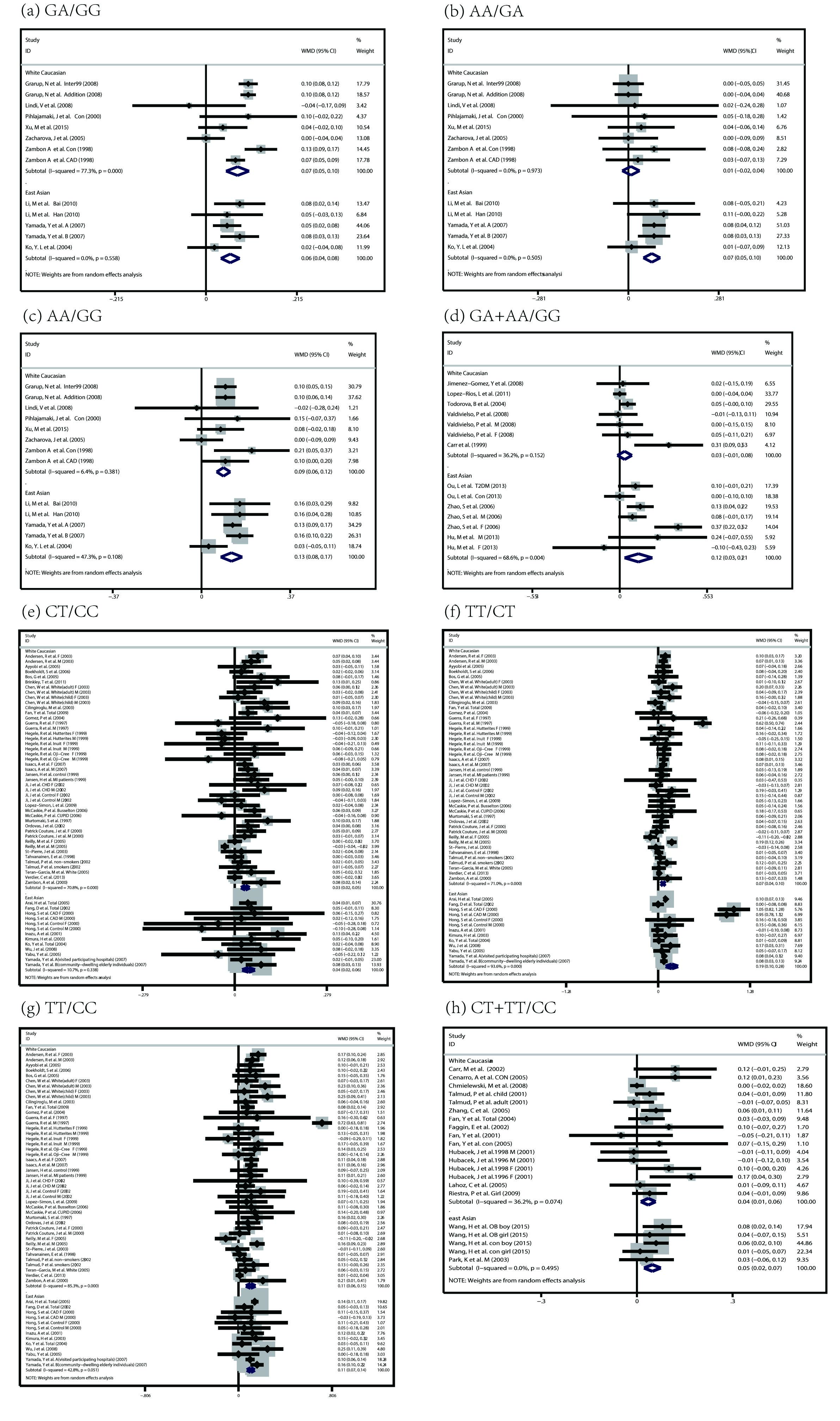
**Figure S4A** Subgroup analysis of HDL-c by race in C-514T and G-250A

**GA/GG AA/GA**

-.215 0 .215 -.281 0 .281

**AA/GG GA+ AA/GG**


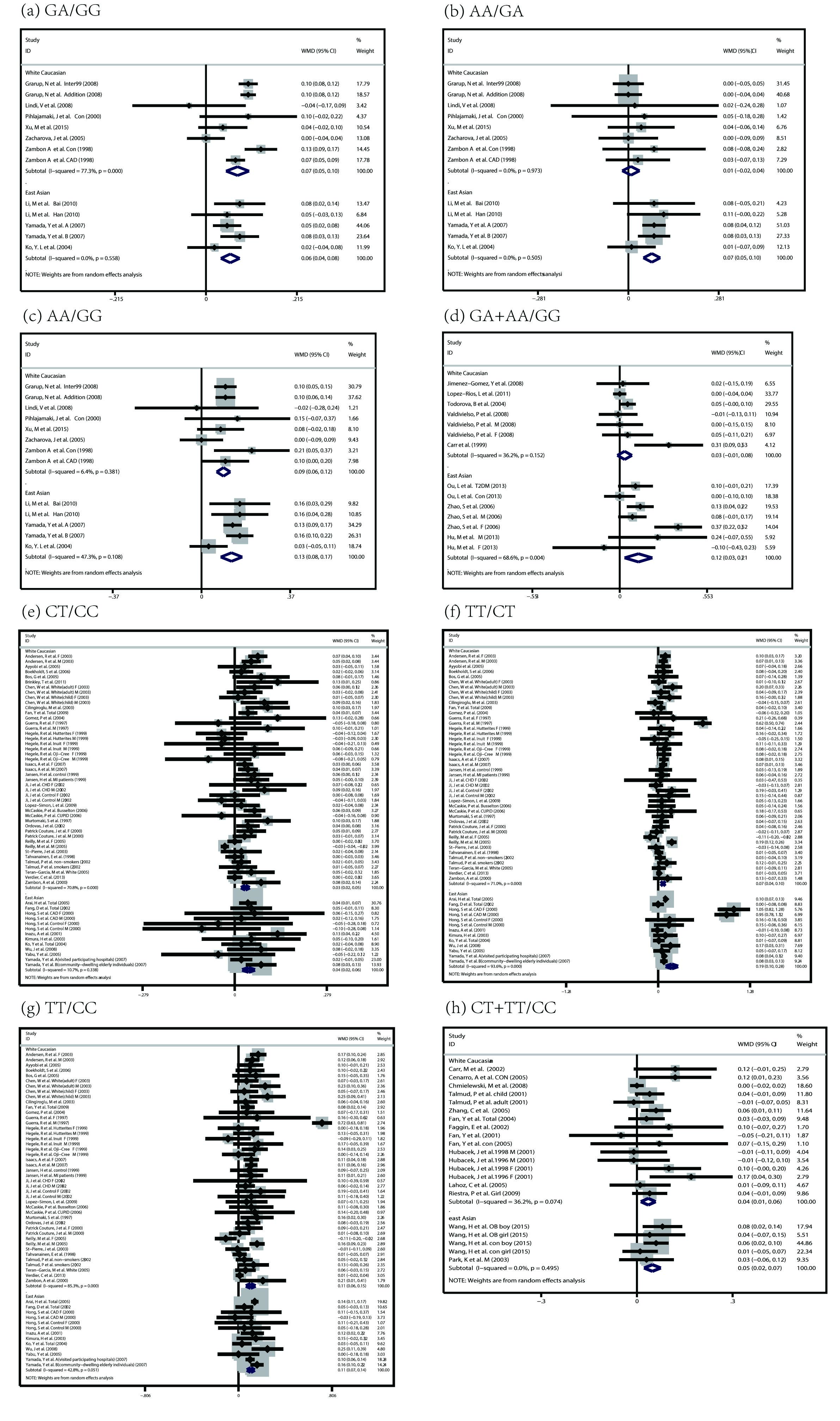


-.37 0 .37 -.553 0 .553


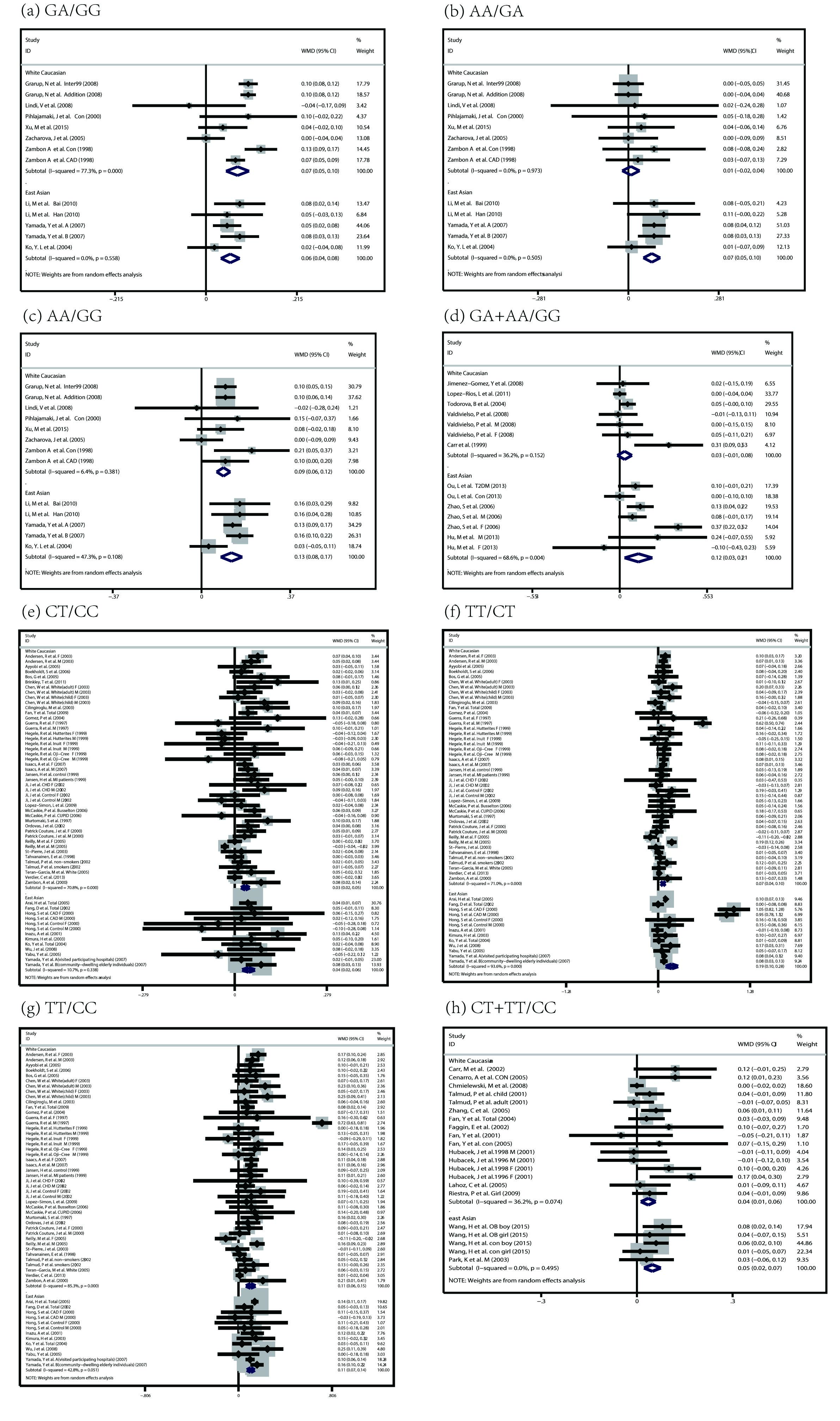

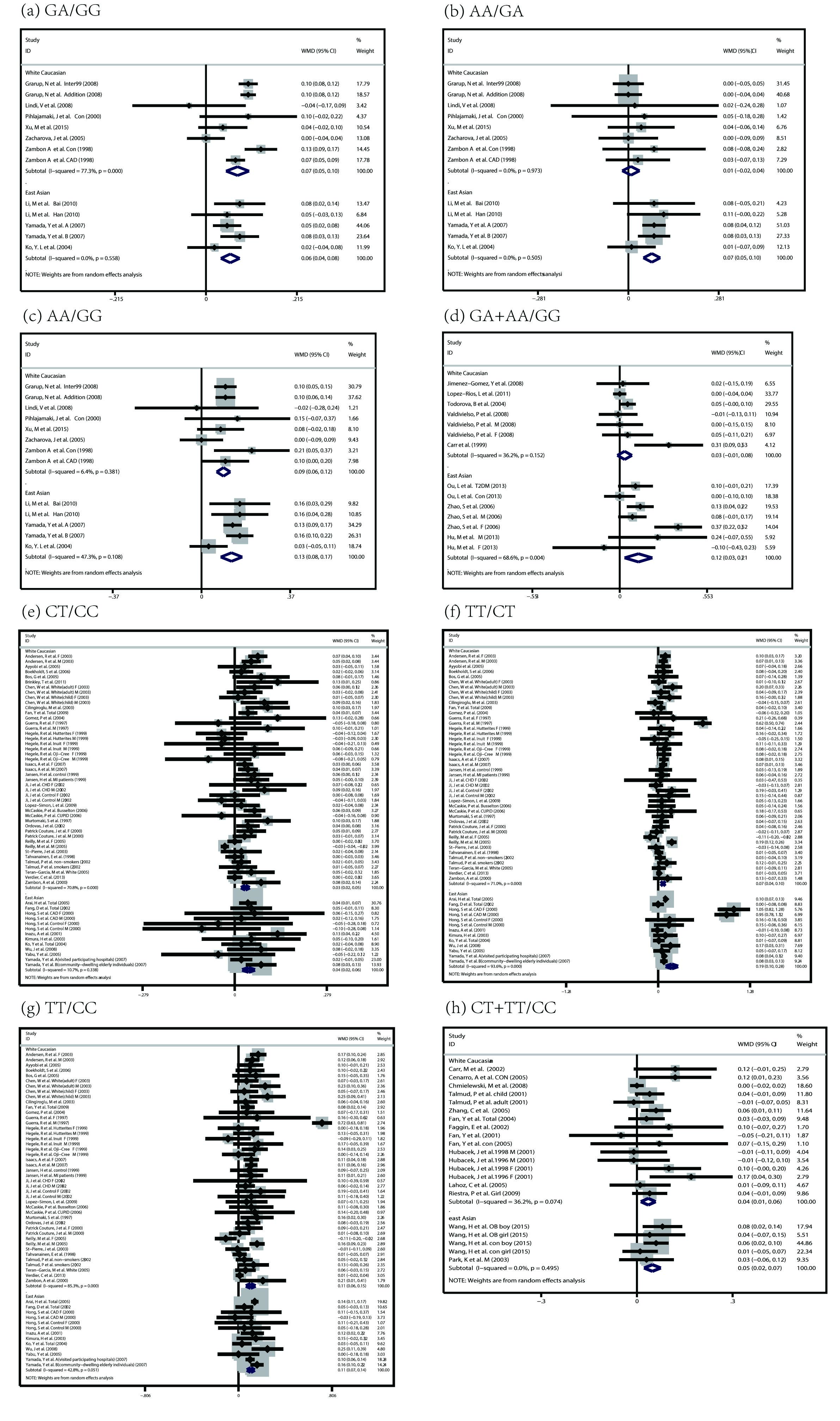


-.3 0 .3

-.806 0 .806

-.27 9 0 .279 -.128 0 .128

**CT/CC TT/CT**


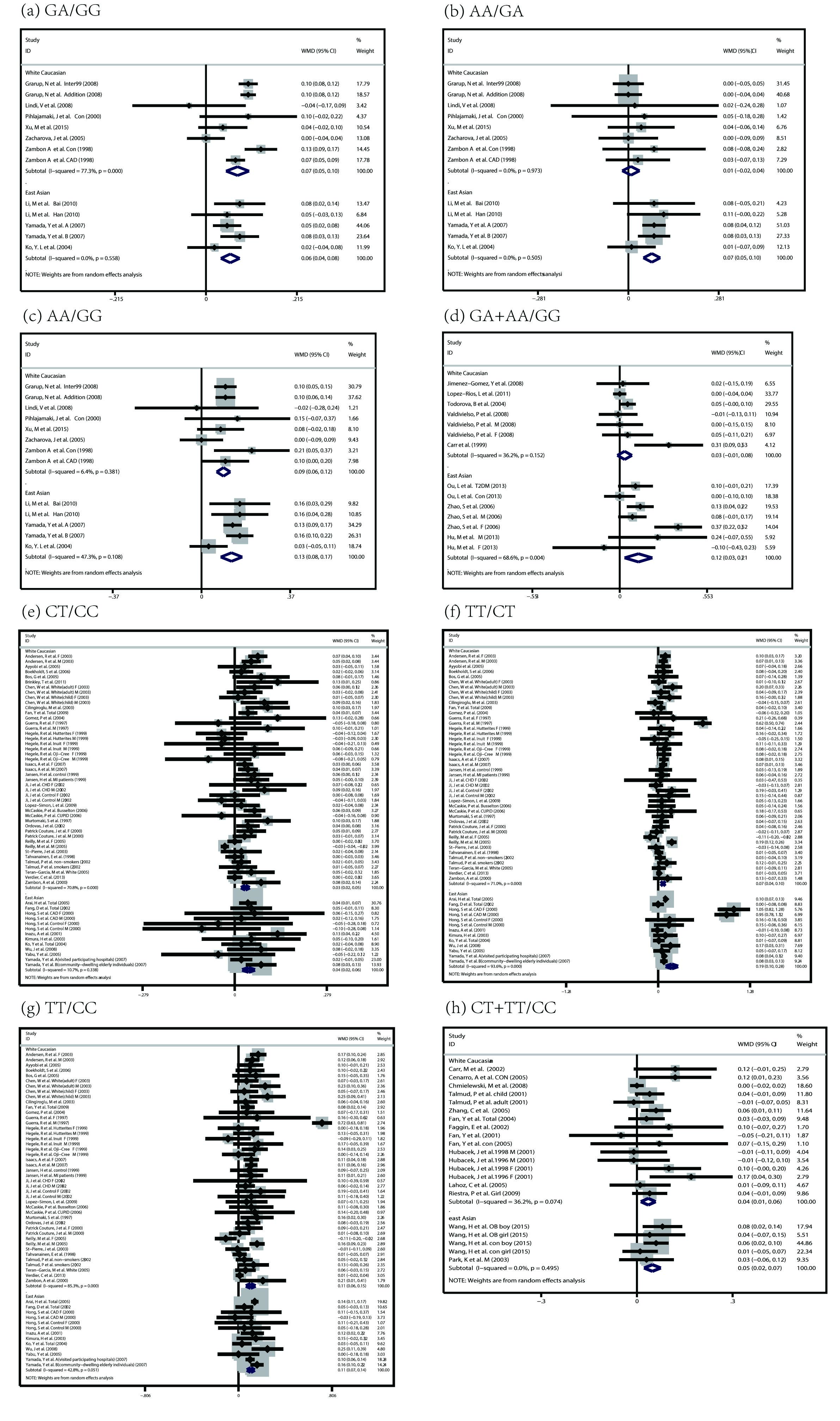


**TT/CC CT + TT/CC**

**Figure S4B** Subgroup analysis of LDL-c by race in C-514T and G-250A

**GA/GG AA/GA**


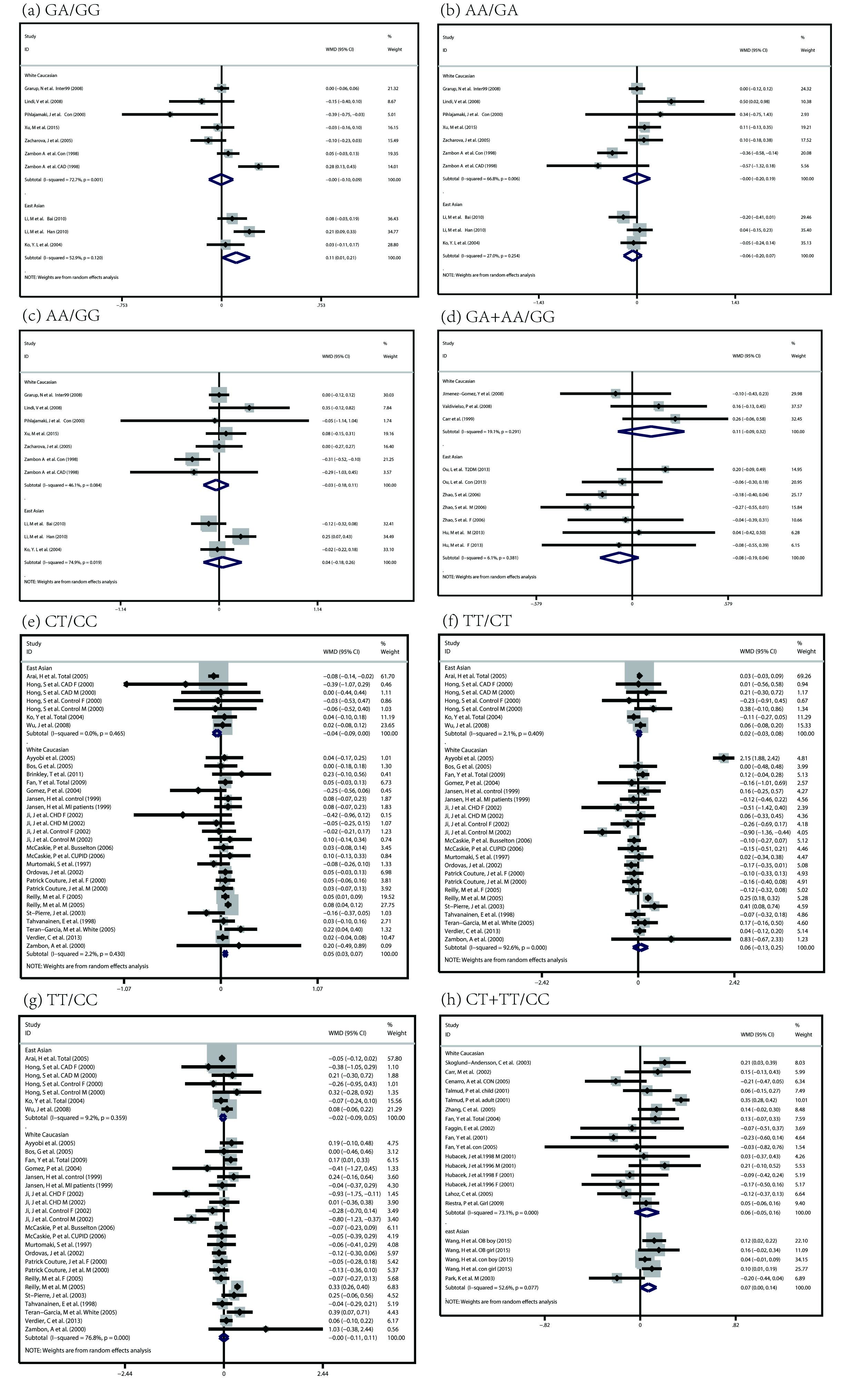

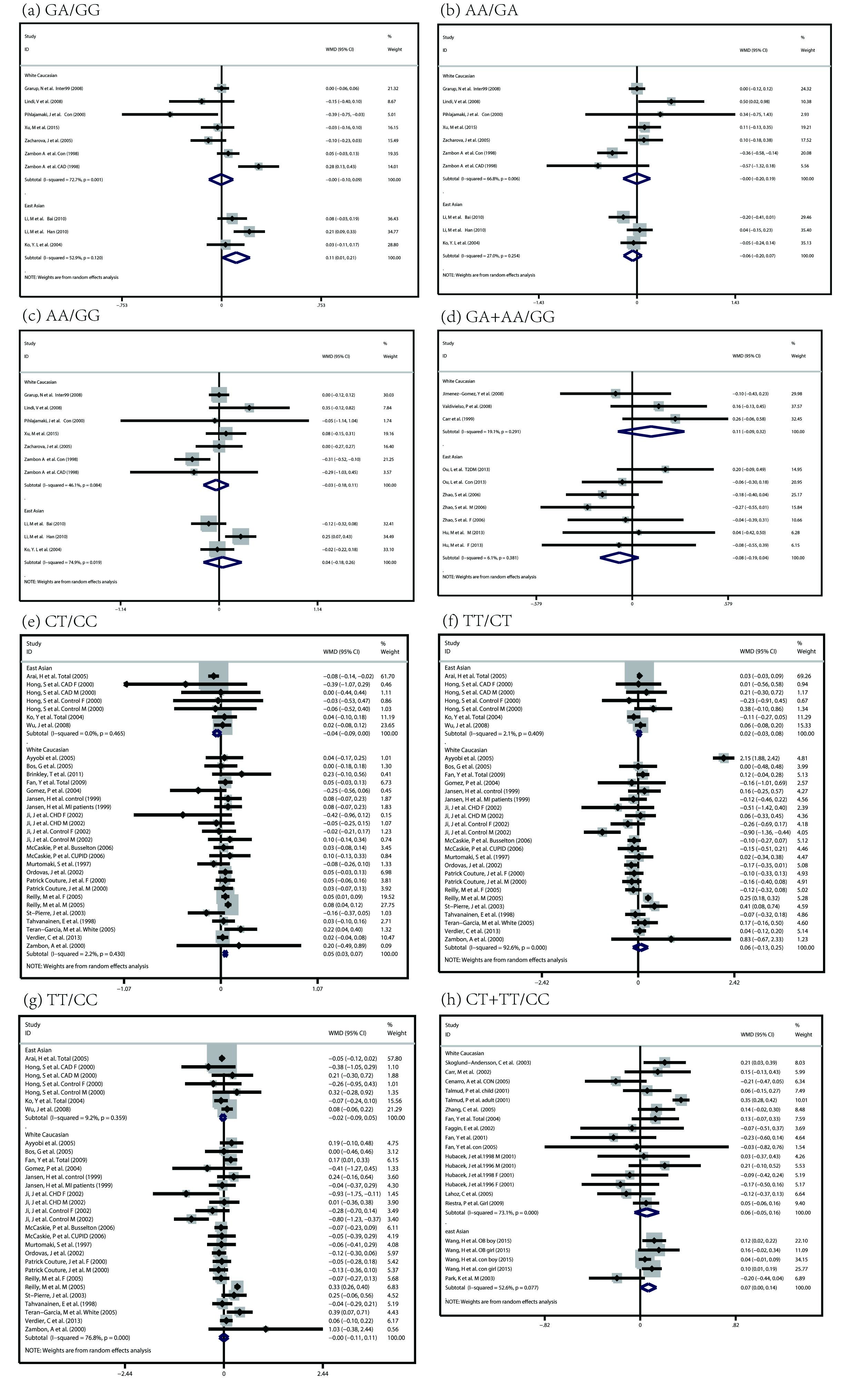

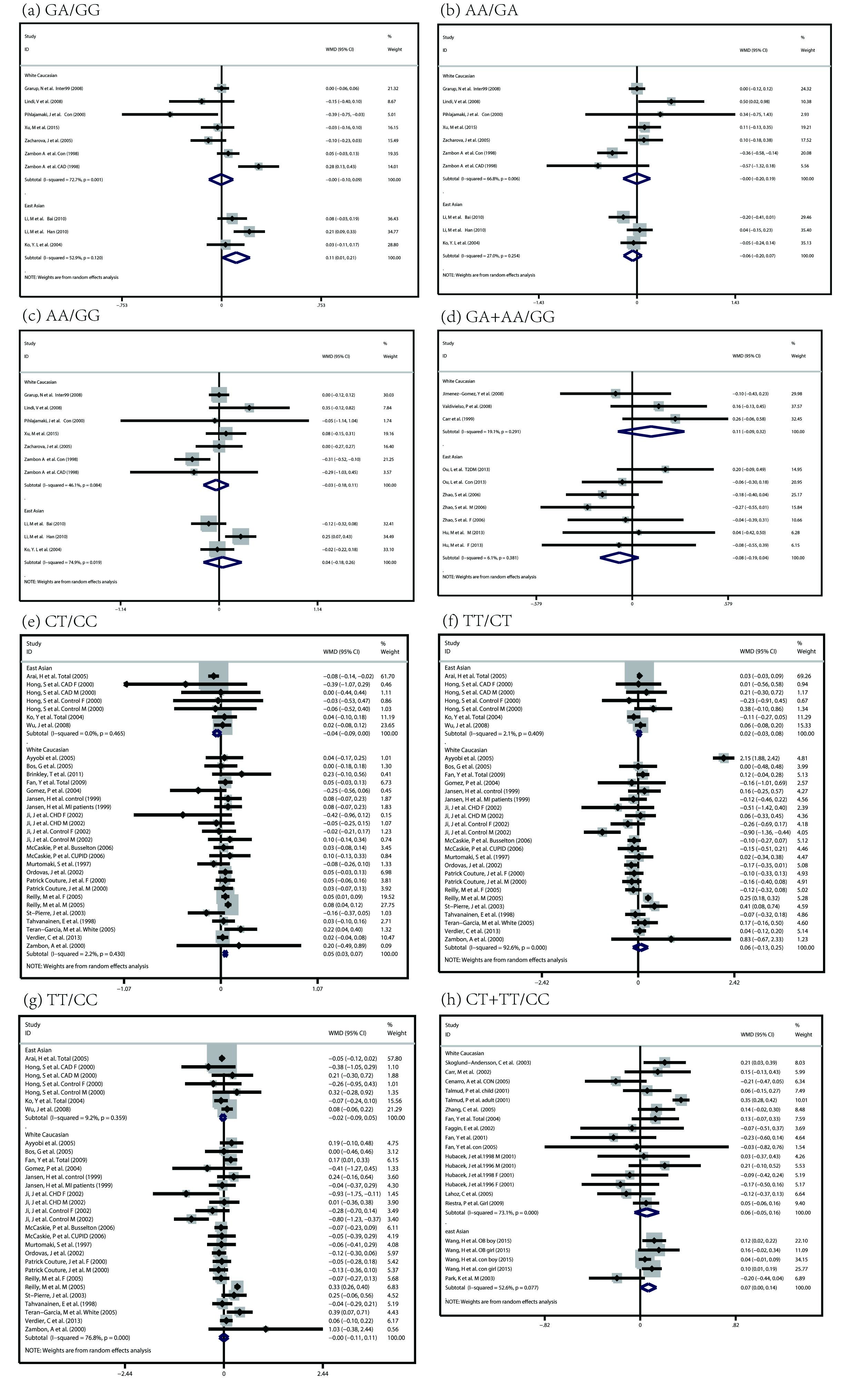


**AA/GG GA+ AA/GG**

-.753 0 .753 -.143 0 .143

-1.14 0 1.14 -.579 0 .579


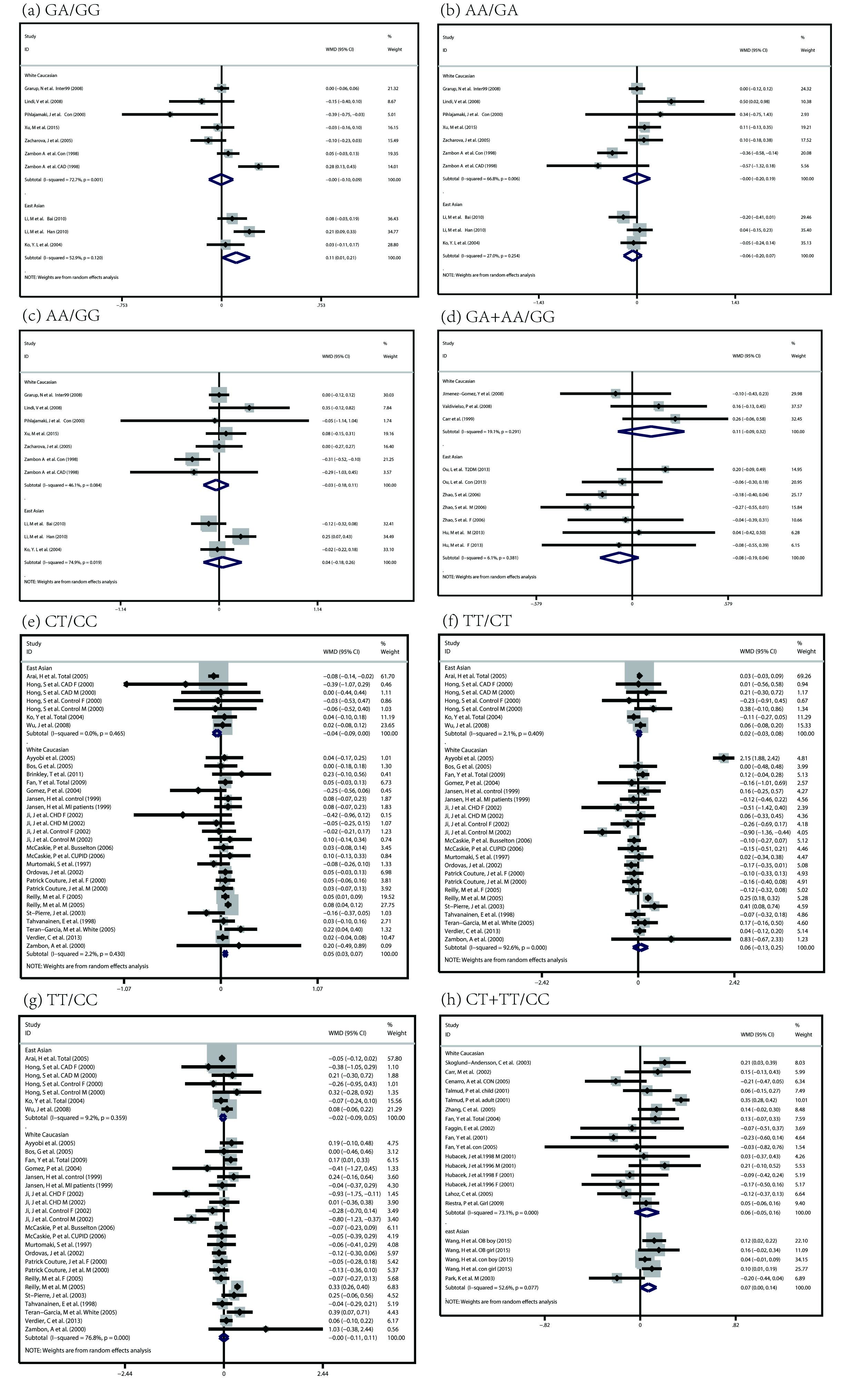

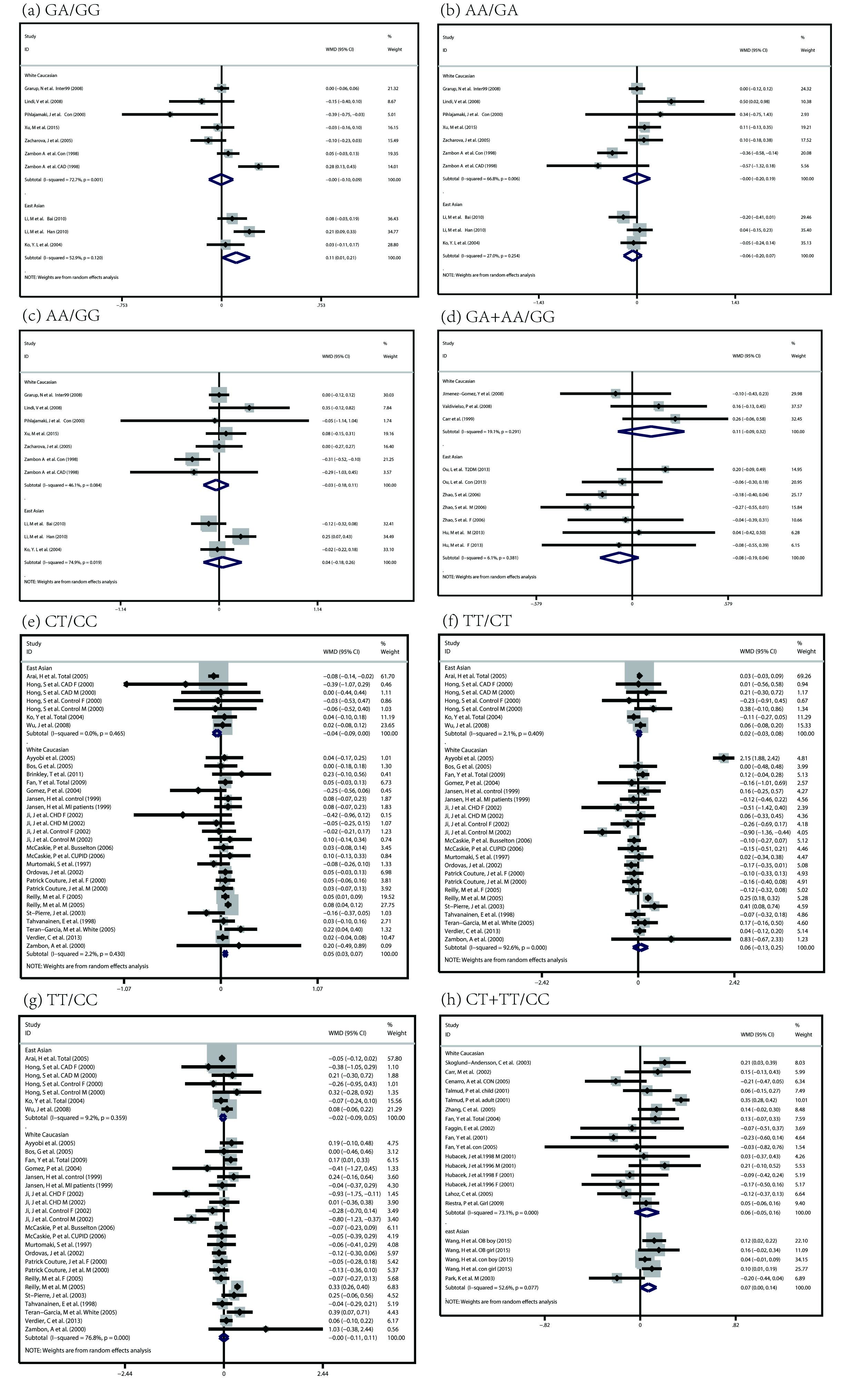


**CT/CC TT/CT**


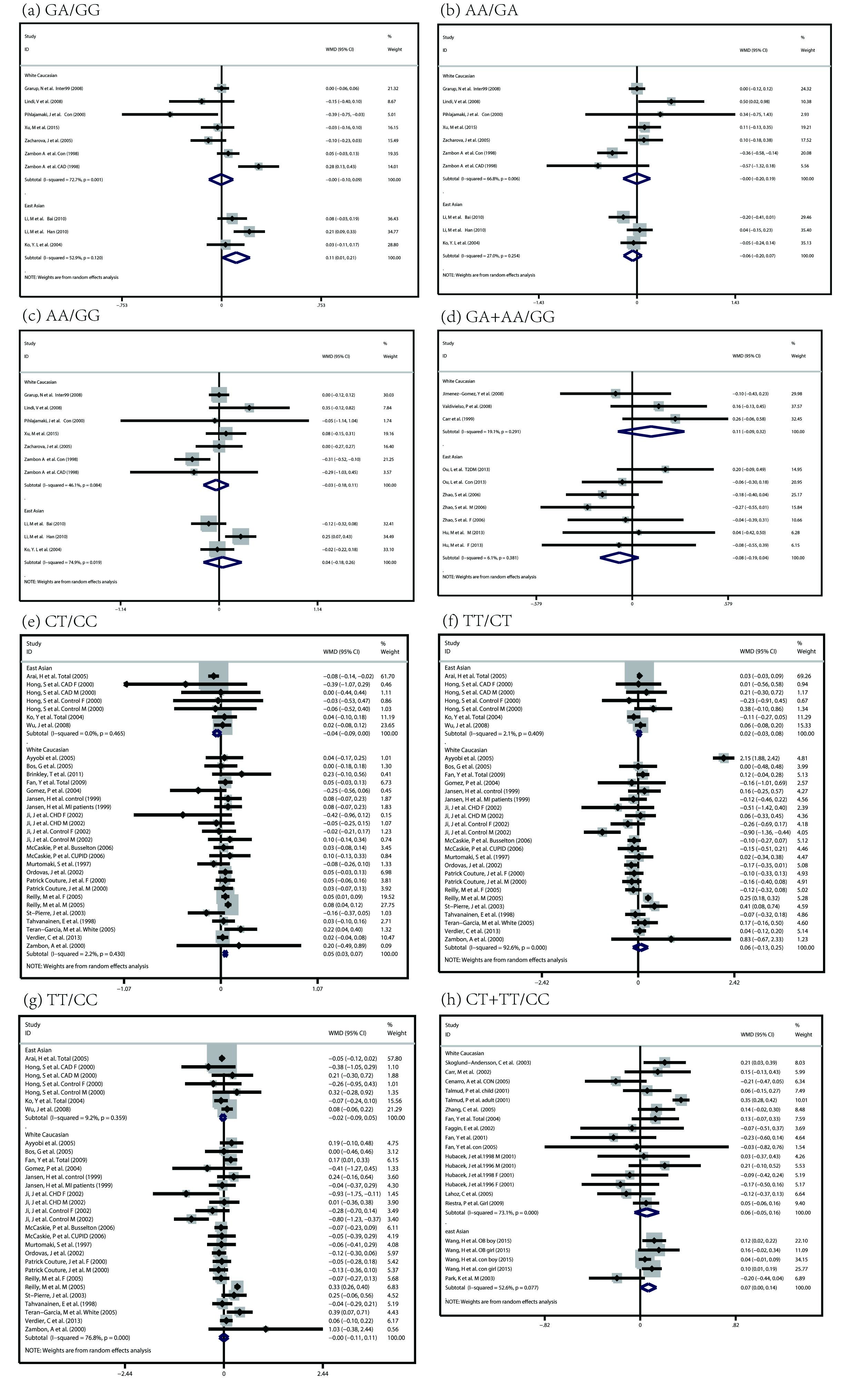

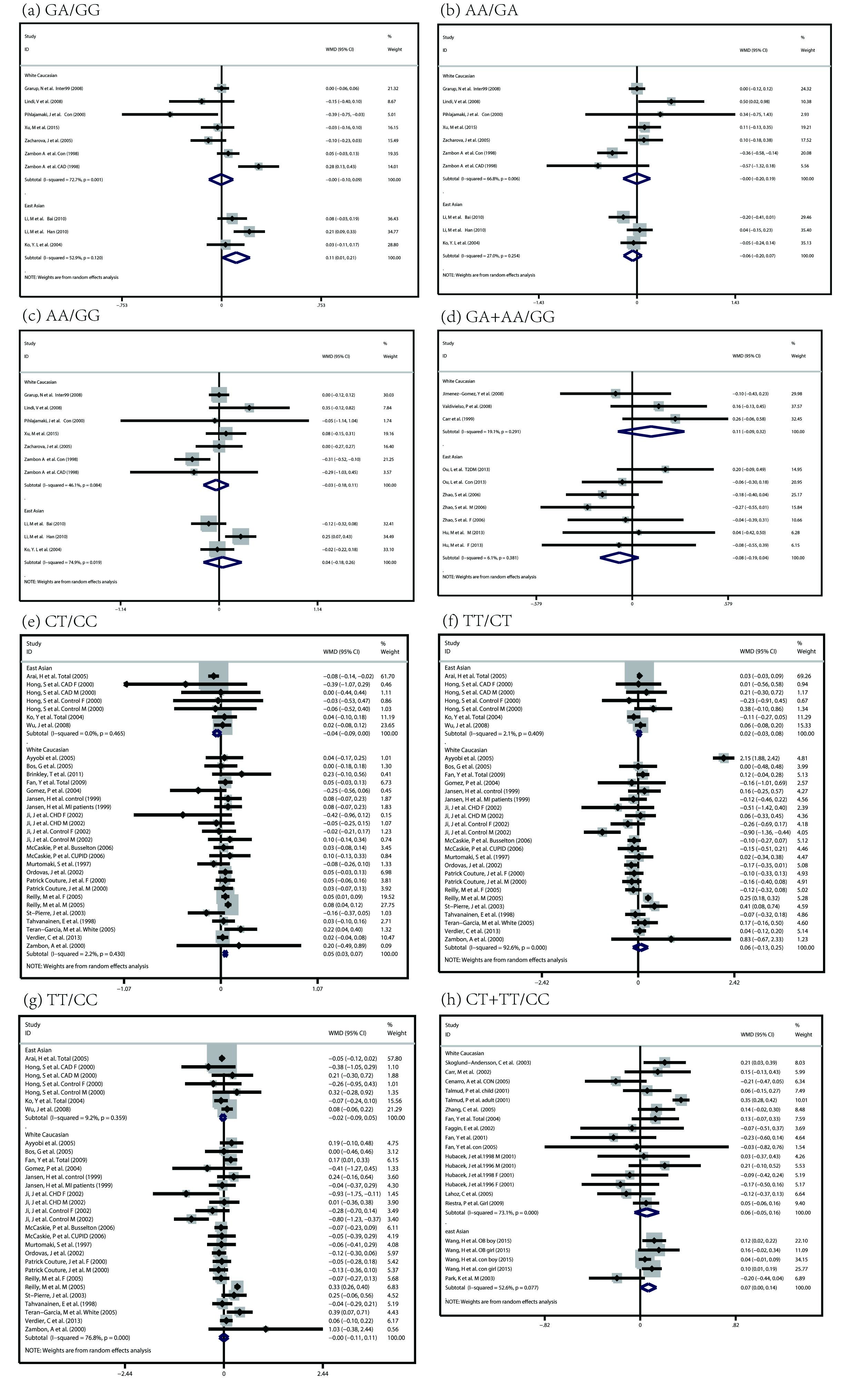


**TT/CC CT + TT/CC**

-1.07 0 1.07 -2.42 0 2.42

-.82 0 .82

-2.44 0 2.44

**Figure S4C** Subgroup analysis of TC by race in C-514T and G-250A


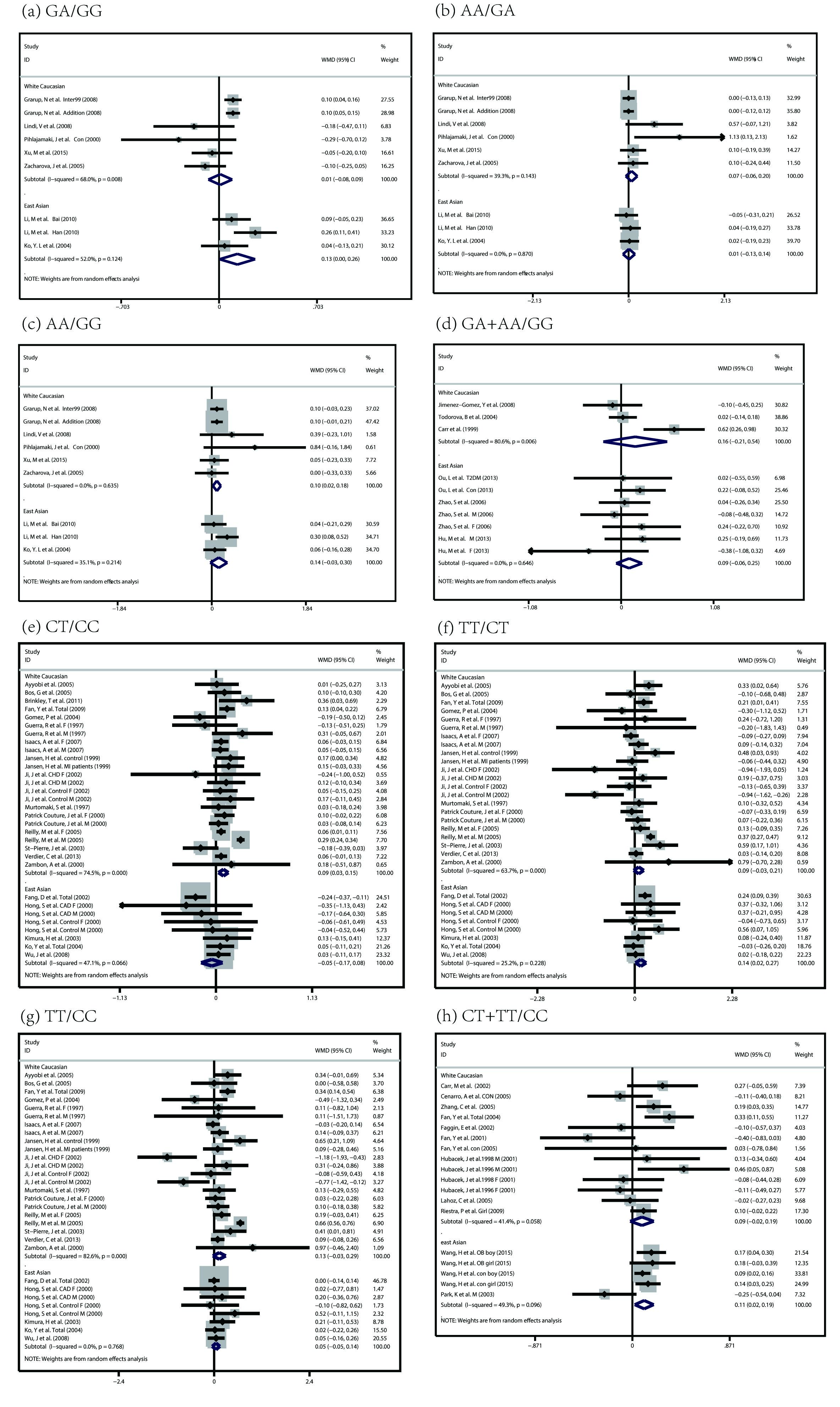

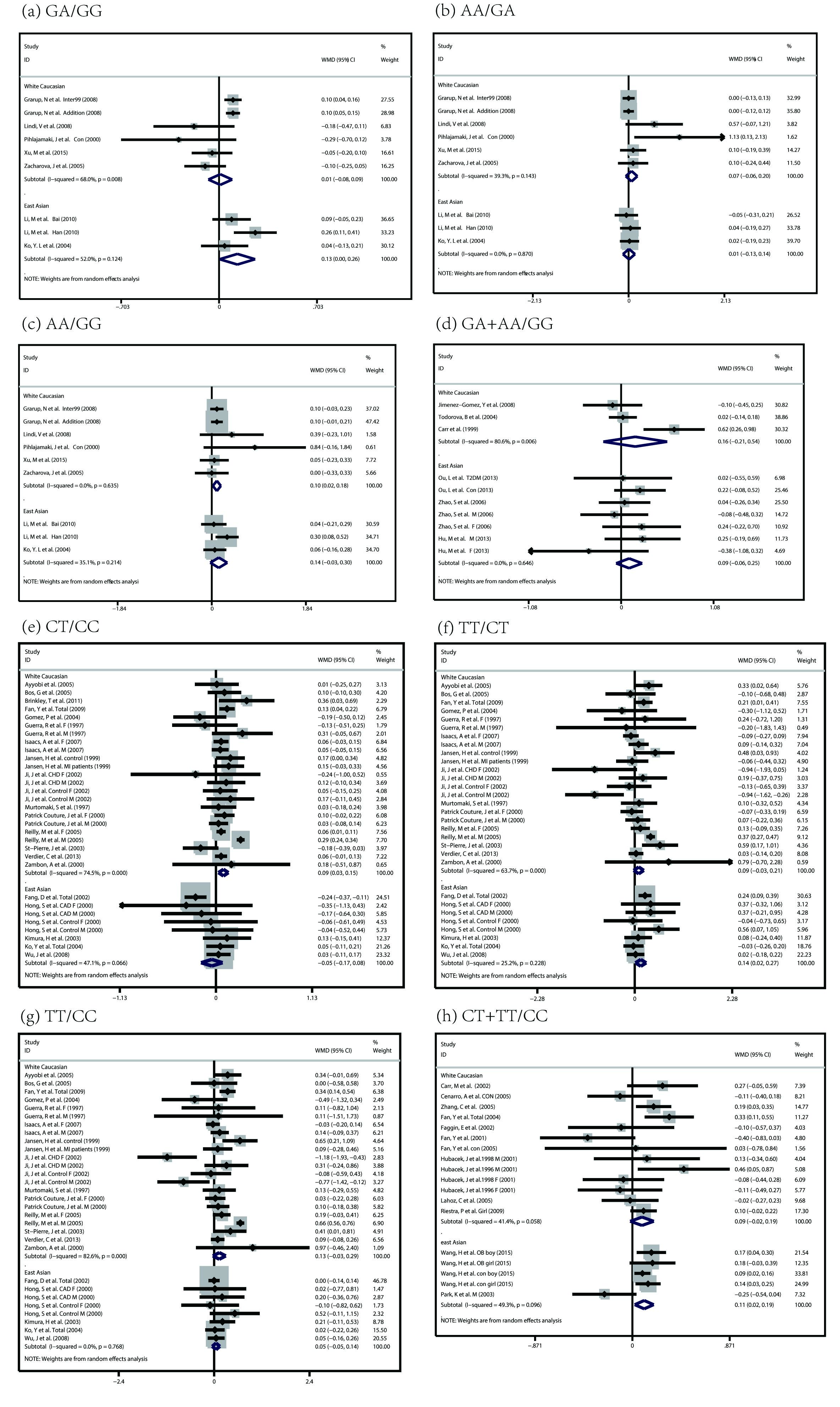


-.703 0 .703 2.13 0 2.13

**GA/GG AA/GA**


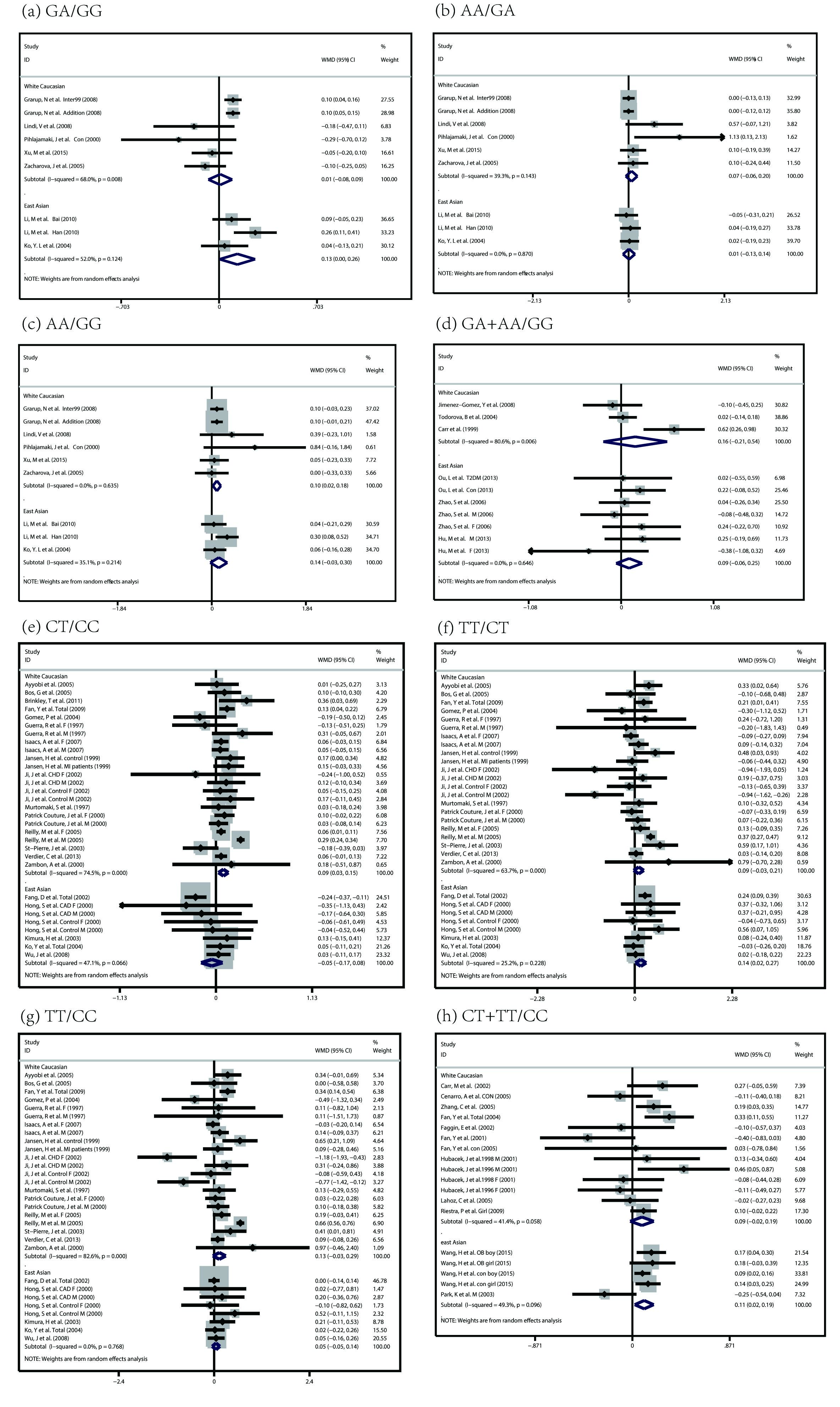

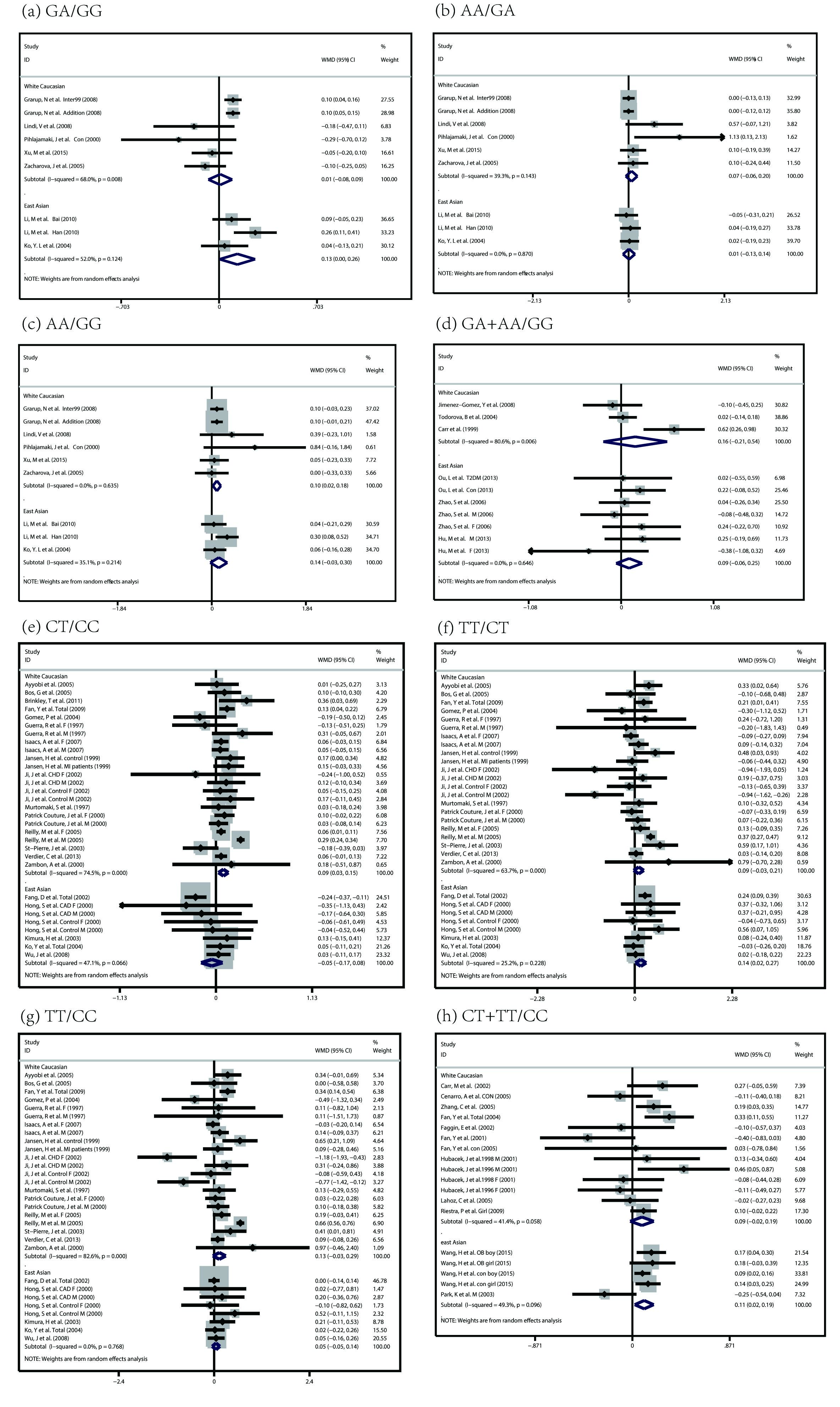


**AA/GG GA + AA/GG**

-1.84 0 1.84 1.08 0 1.08


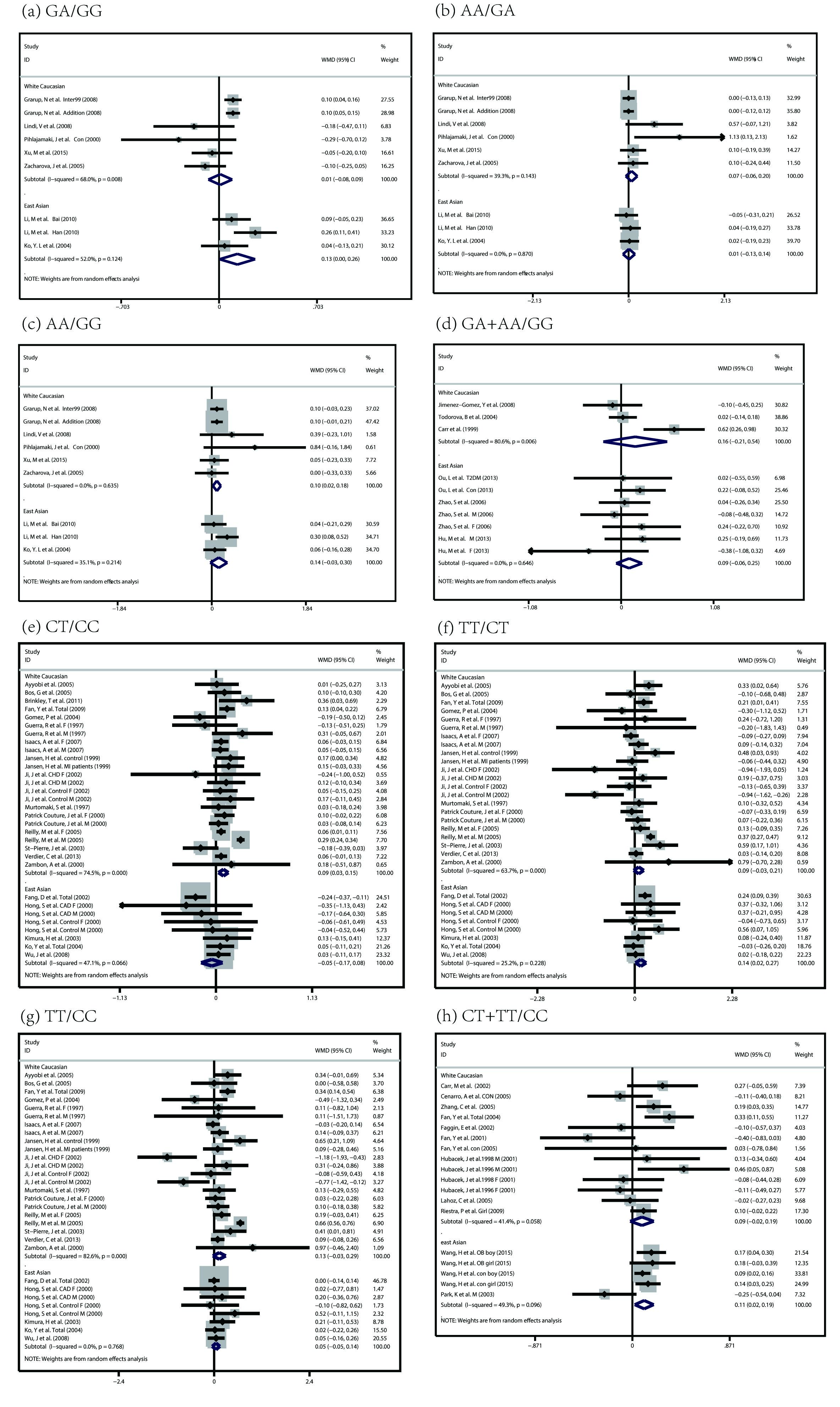


**CT/CC TT/CT**


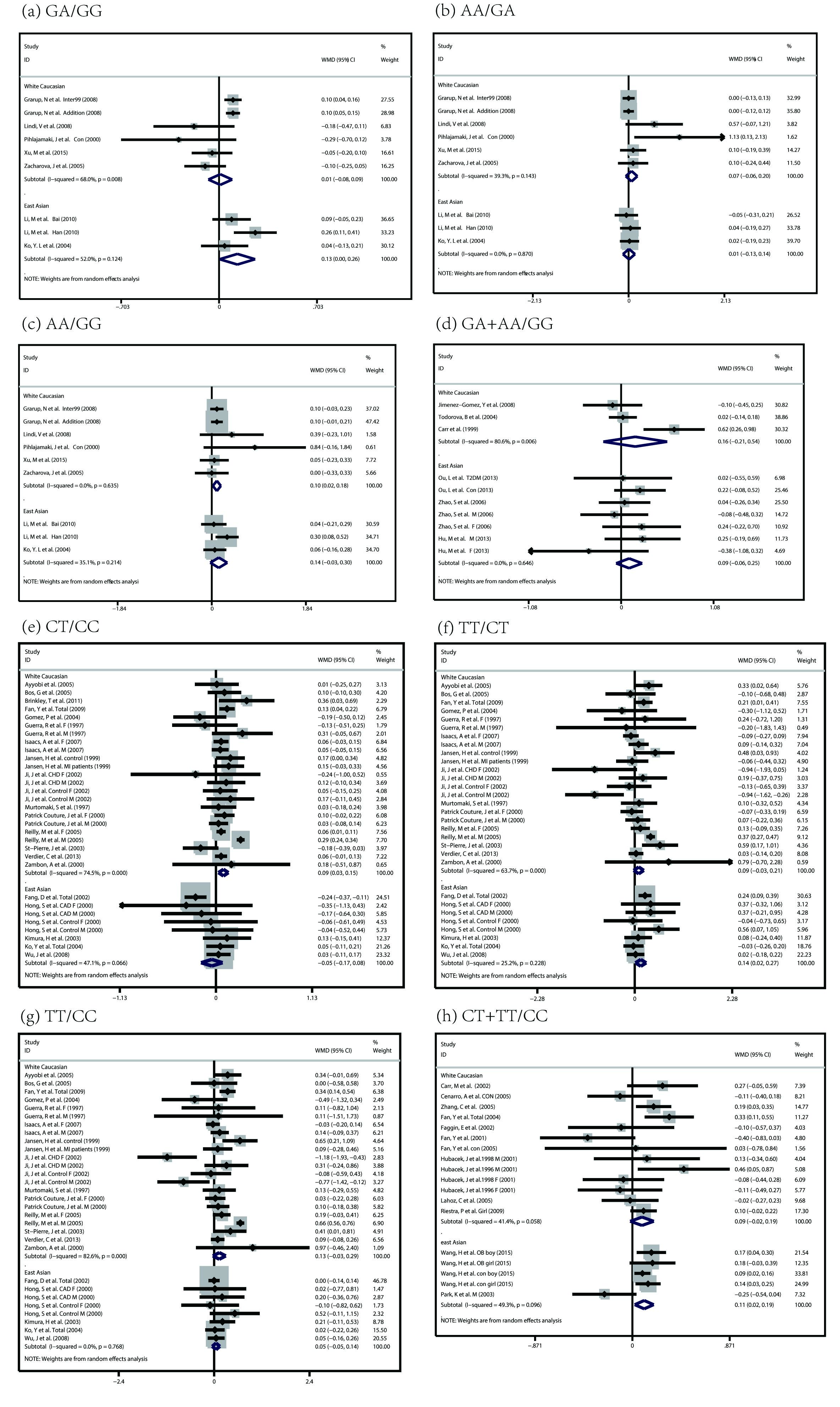

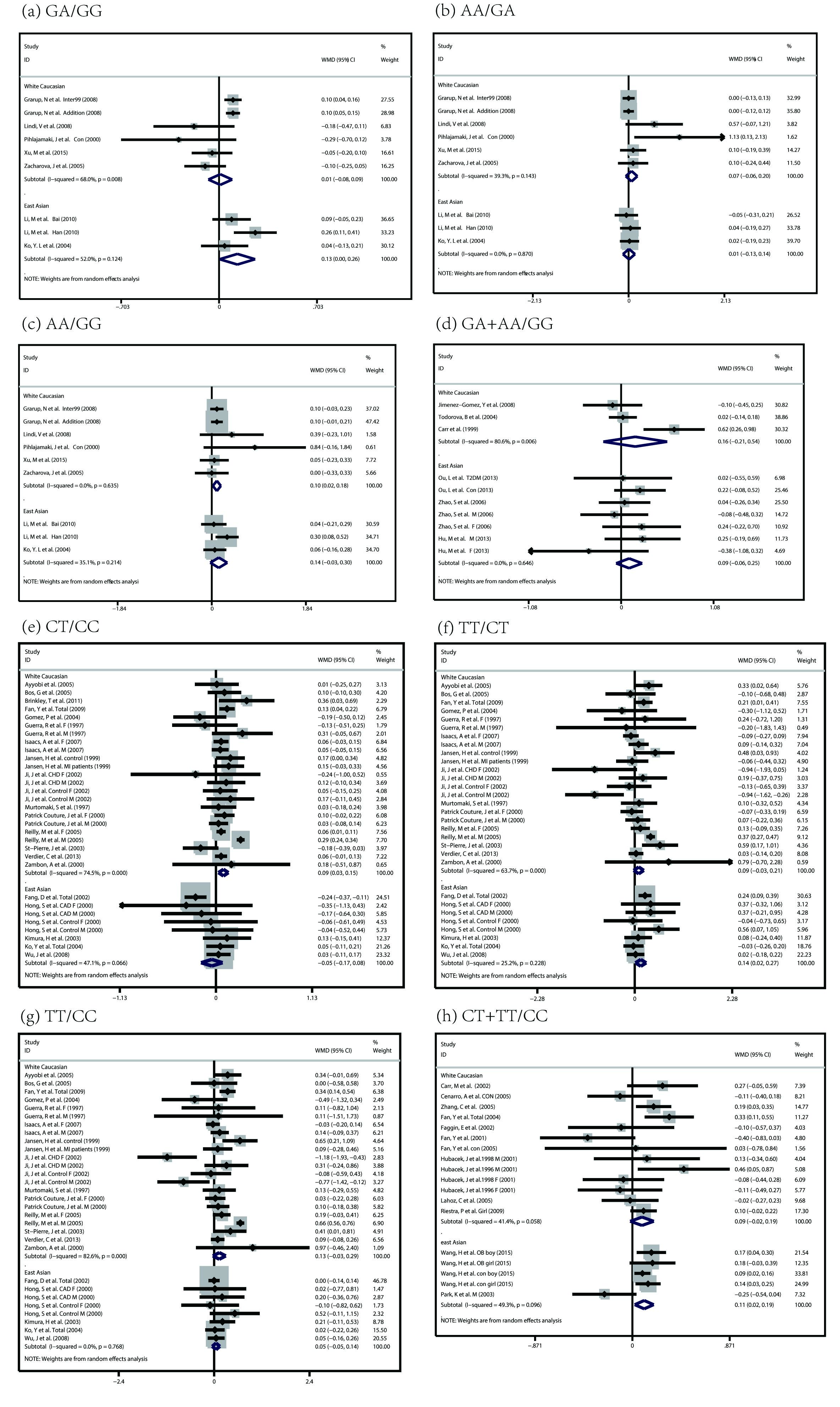


**TT/CC CT + TT/CC**

-1.13 0 1.13 -2.28 0 2.28

-.871 0 .871

-2.4 0 2.4

**Figure S4D** Subgroup analysis of TG by race in C-514T and G-250A


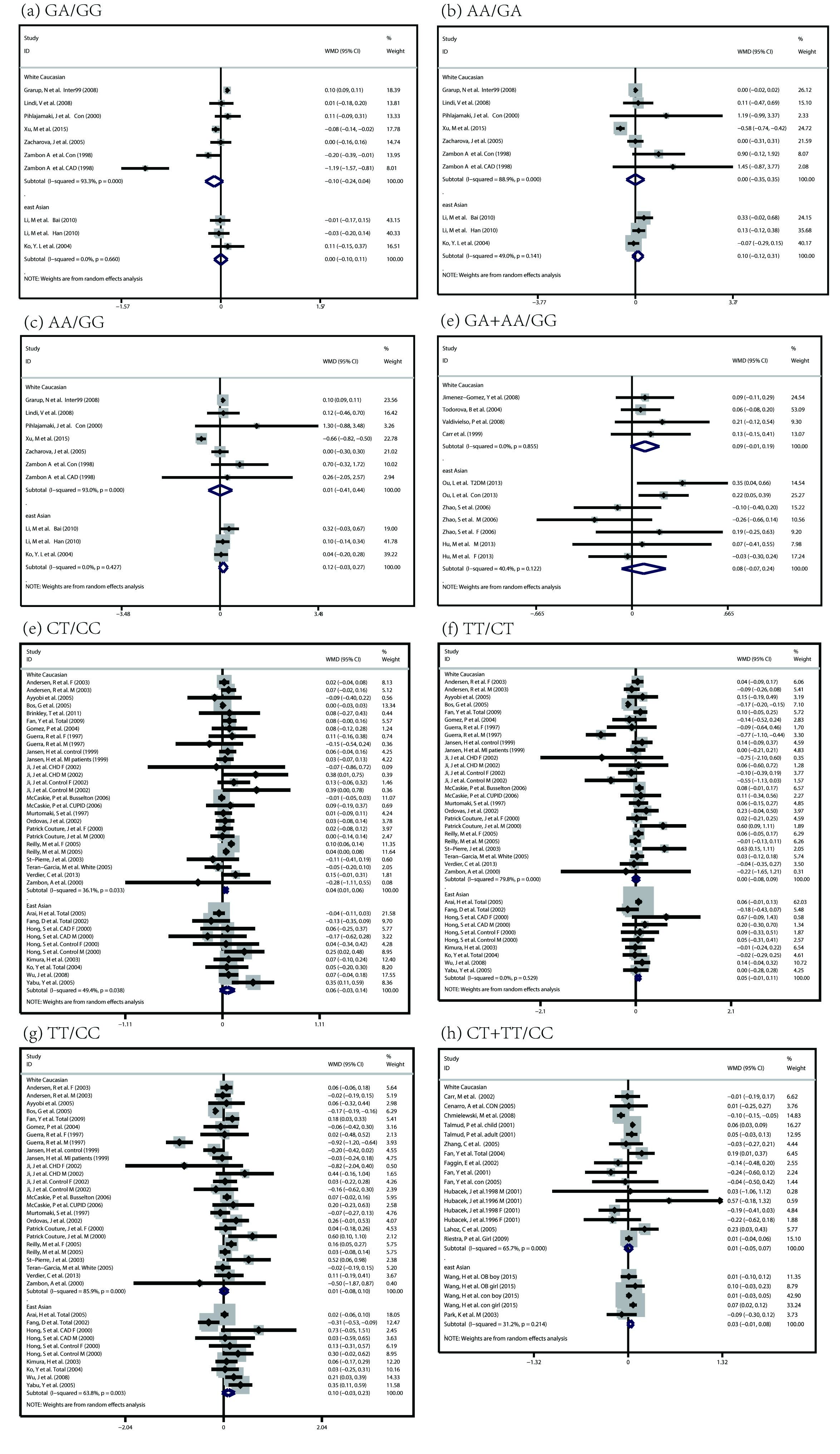

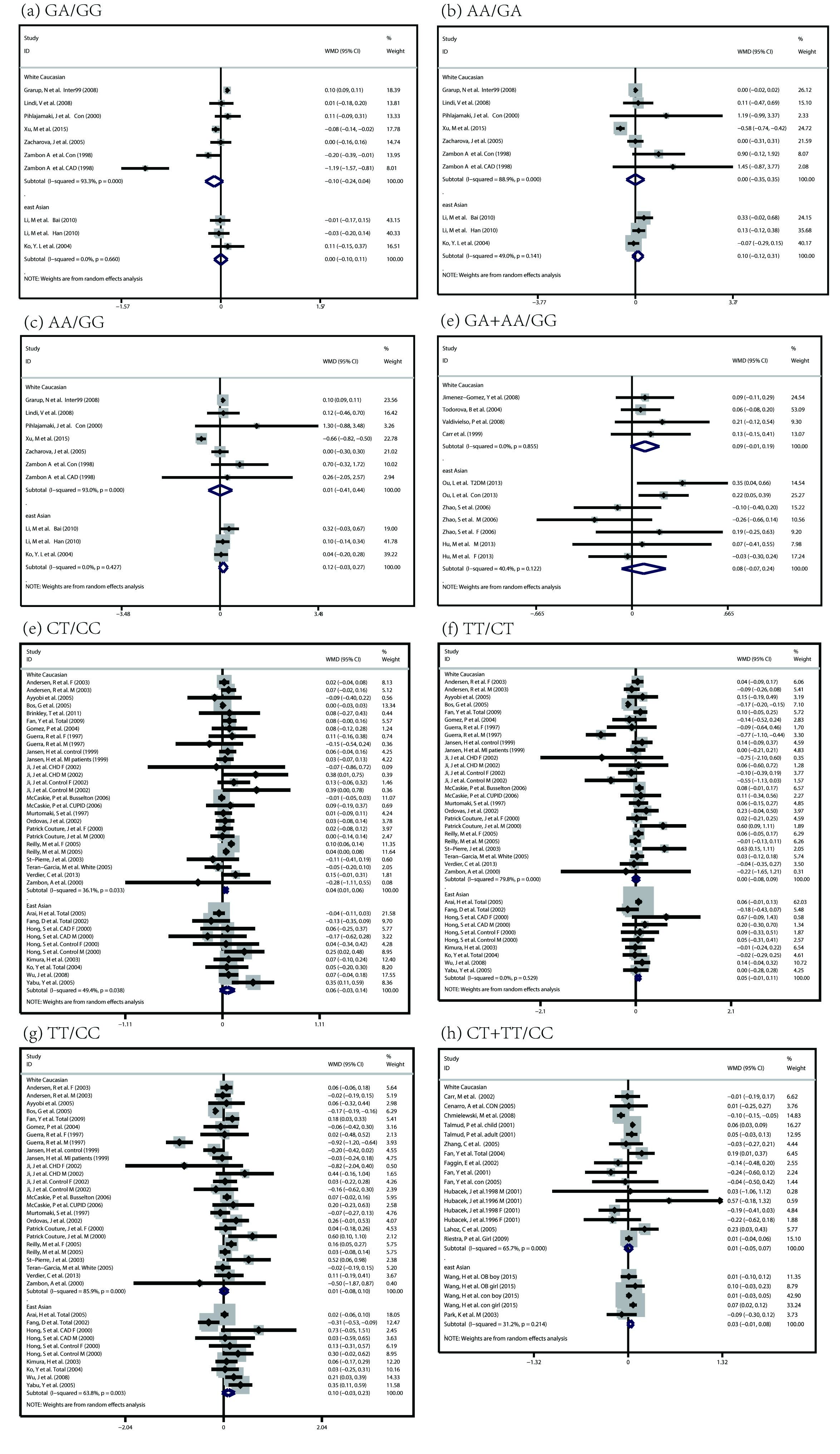


**GA/GG AA/GA**

-1.57 0 1.57 -3.77 0 3.77

**AA/GG GA + AA/GG**


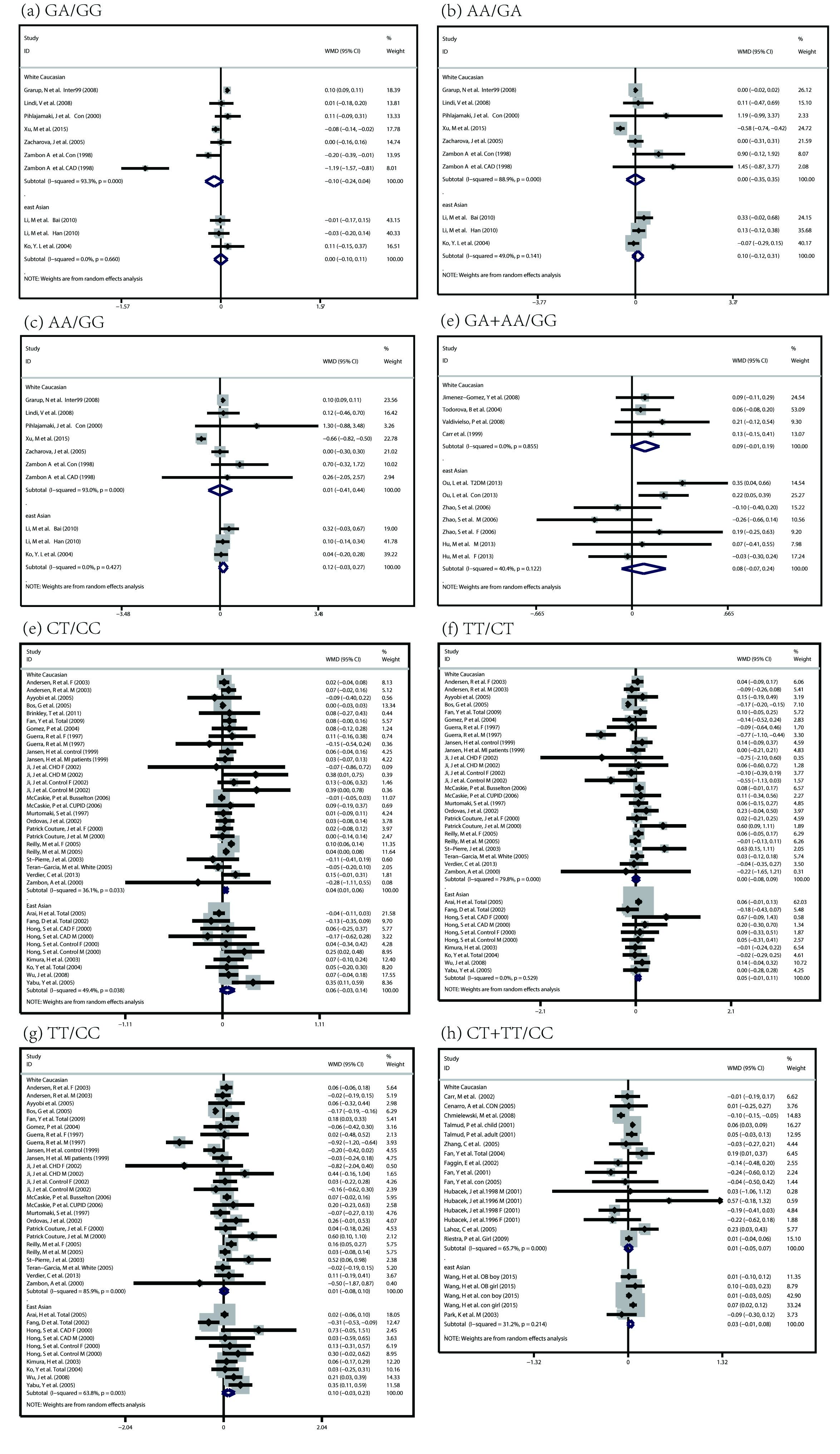


-3.48 0 3.48 -.665 0 .665


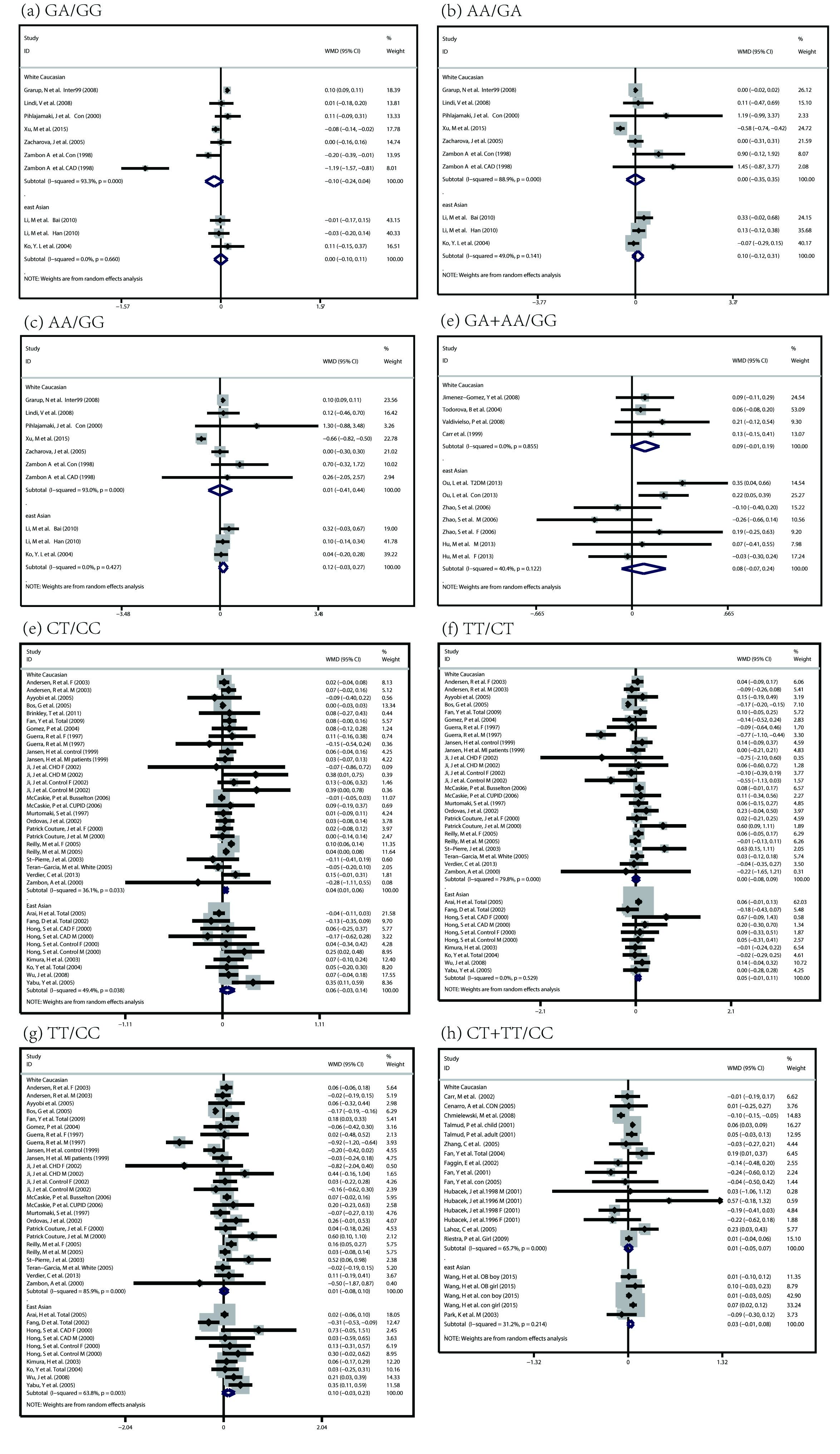

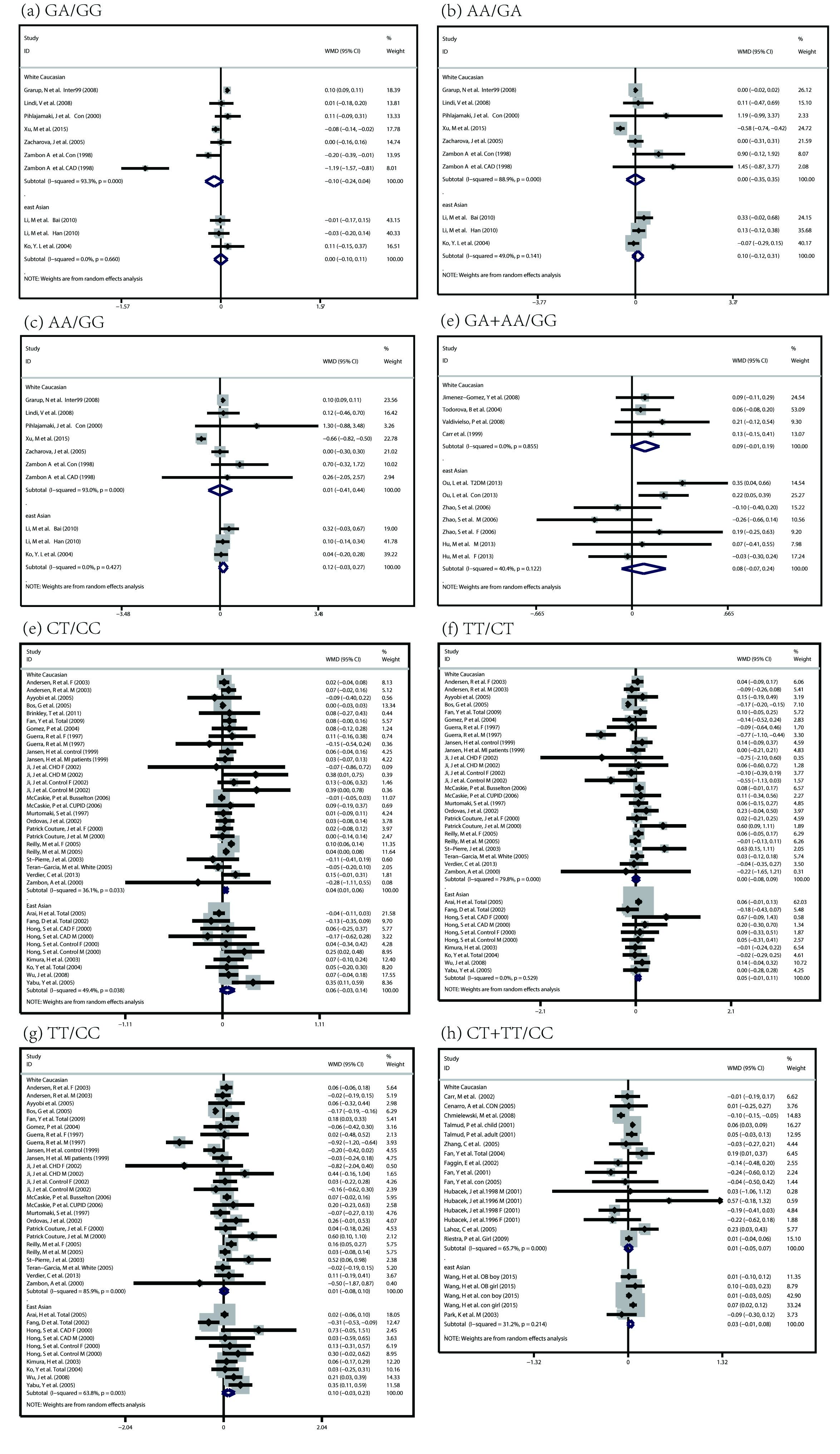

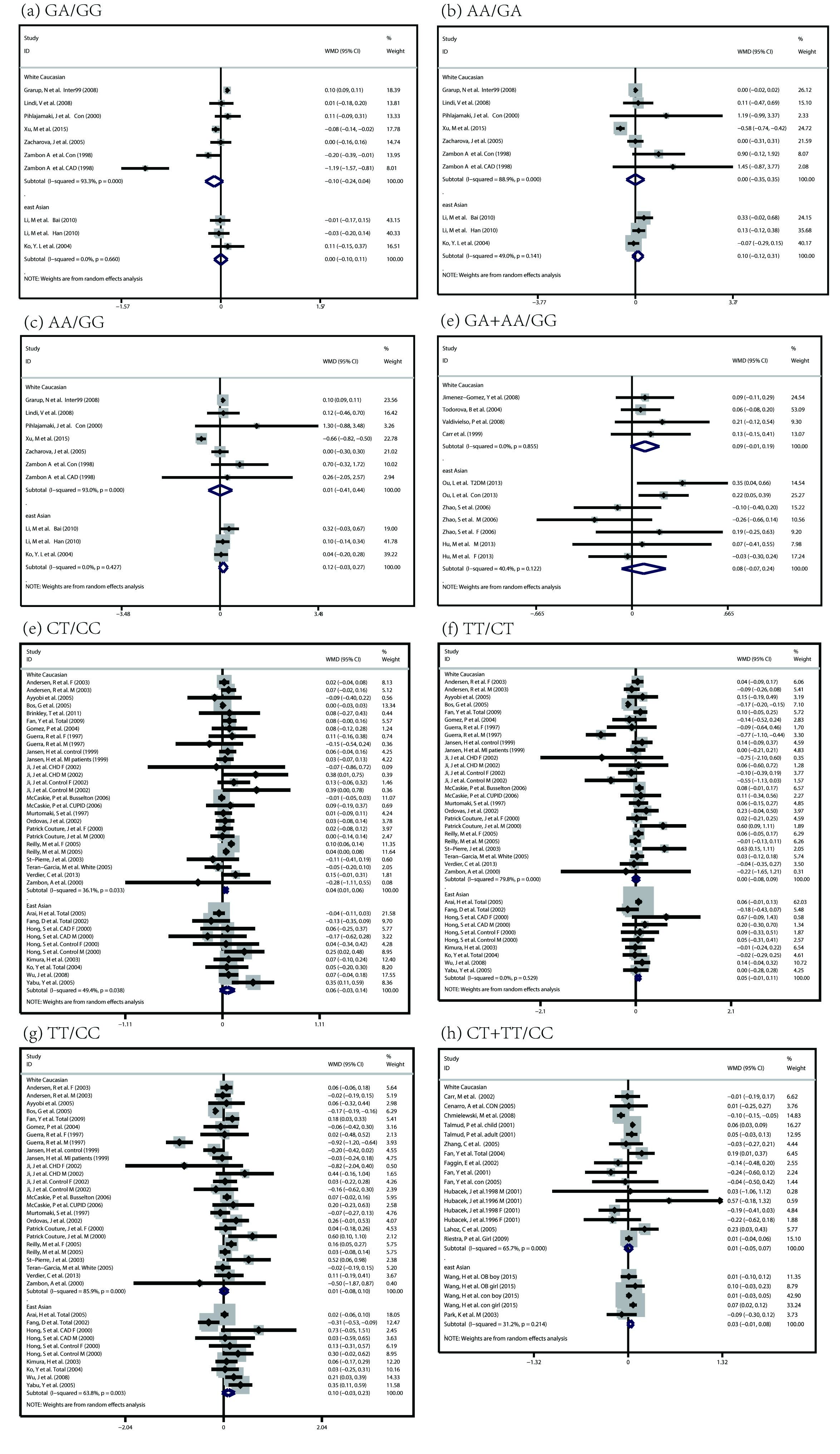

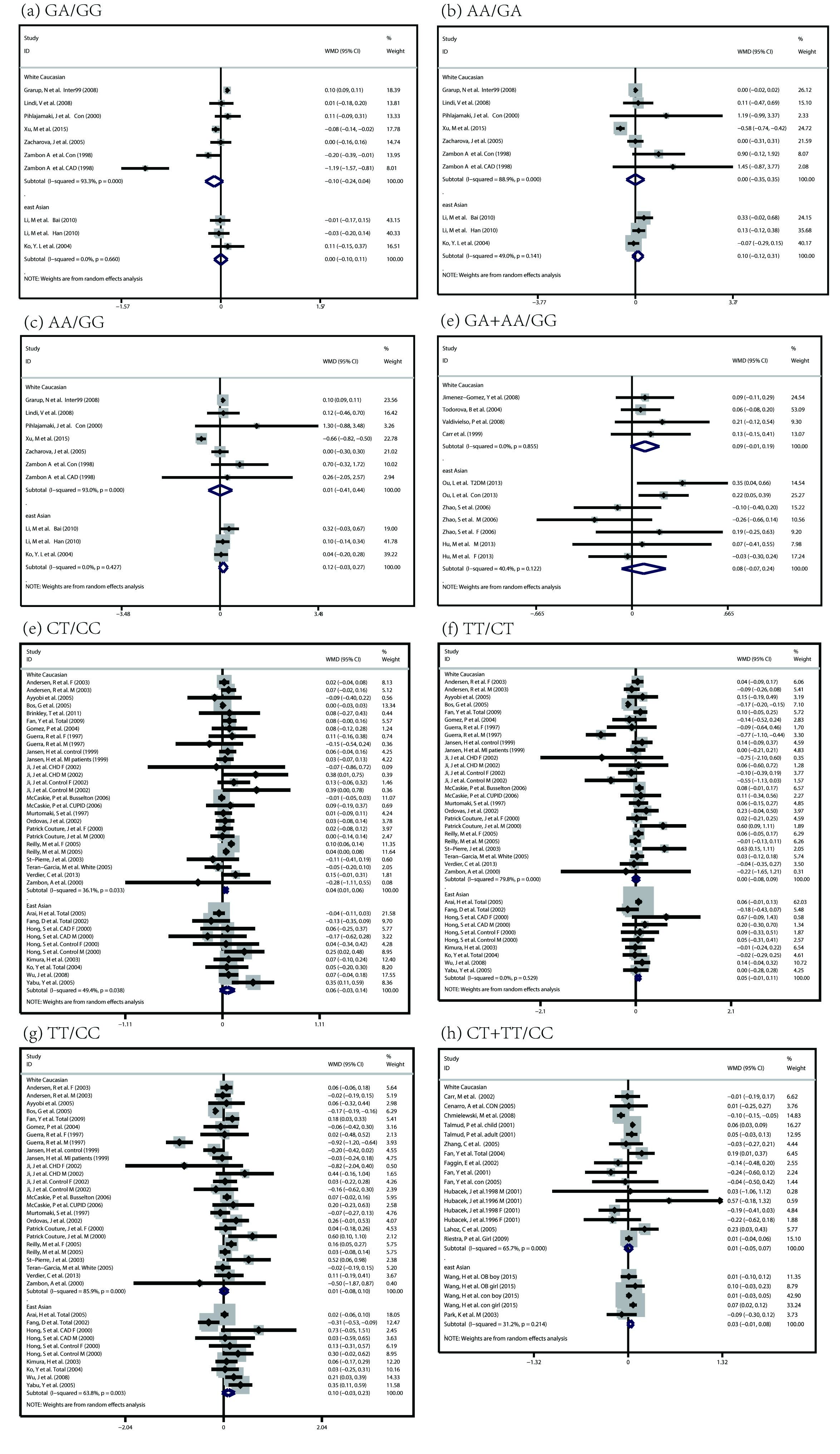


**CT/CC TT/CT**

**TT/CC CT + TT/CC**

-1.11 0 1.11 -2.1 0 2.1

**TT/CC CT + TT/CC**

-1.32 0 1.32

-2.04 0 2.04

**Figure S4E** Subgroup analysis of BMI by race in C-514T and G-250A


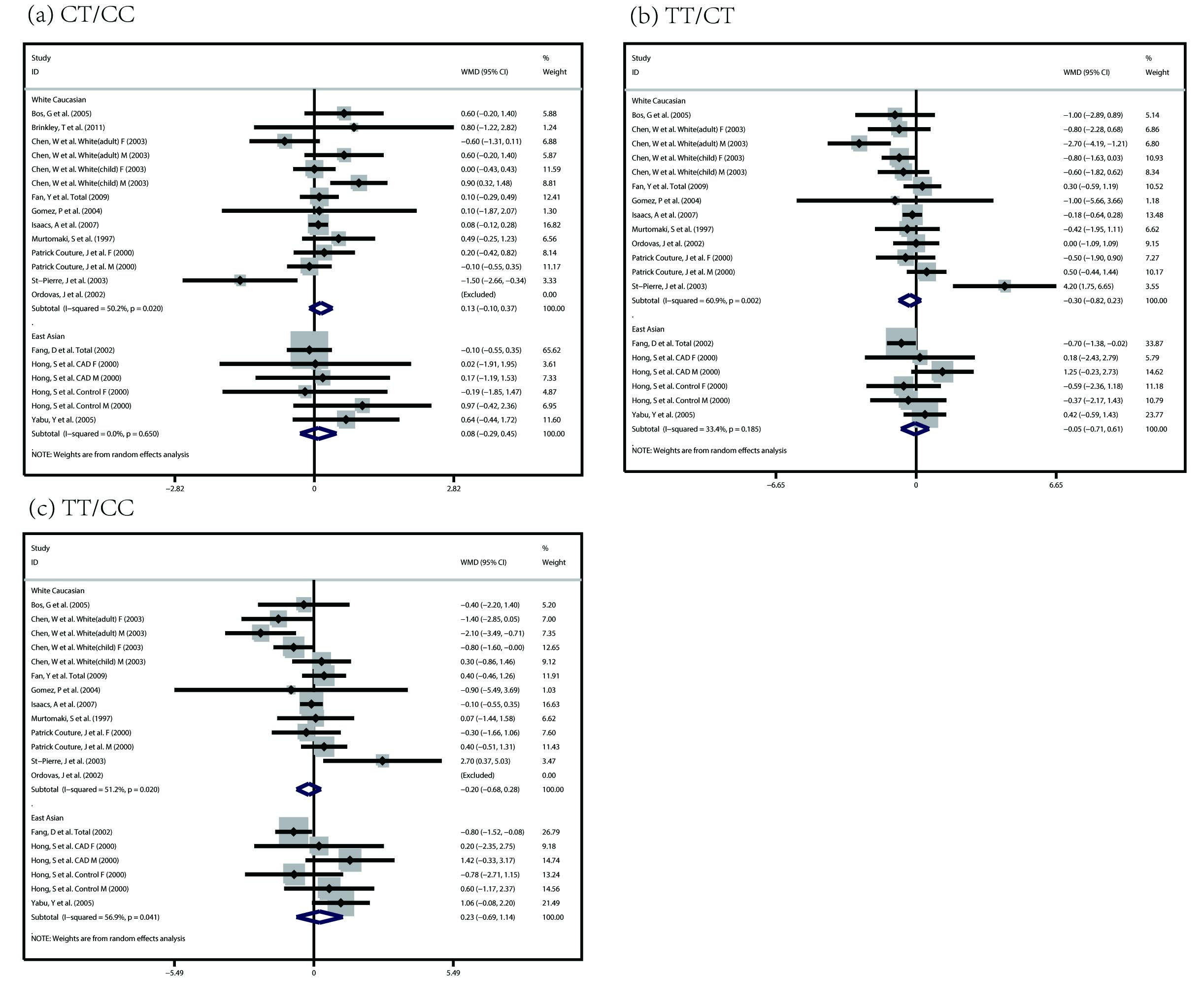

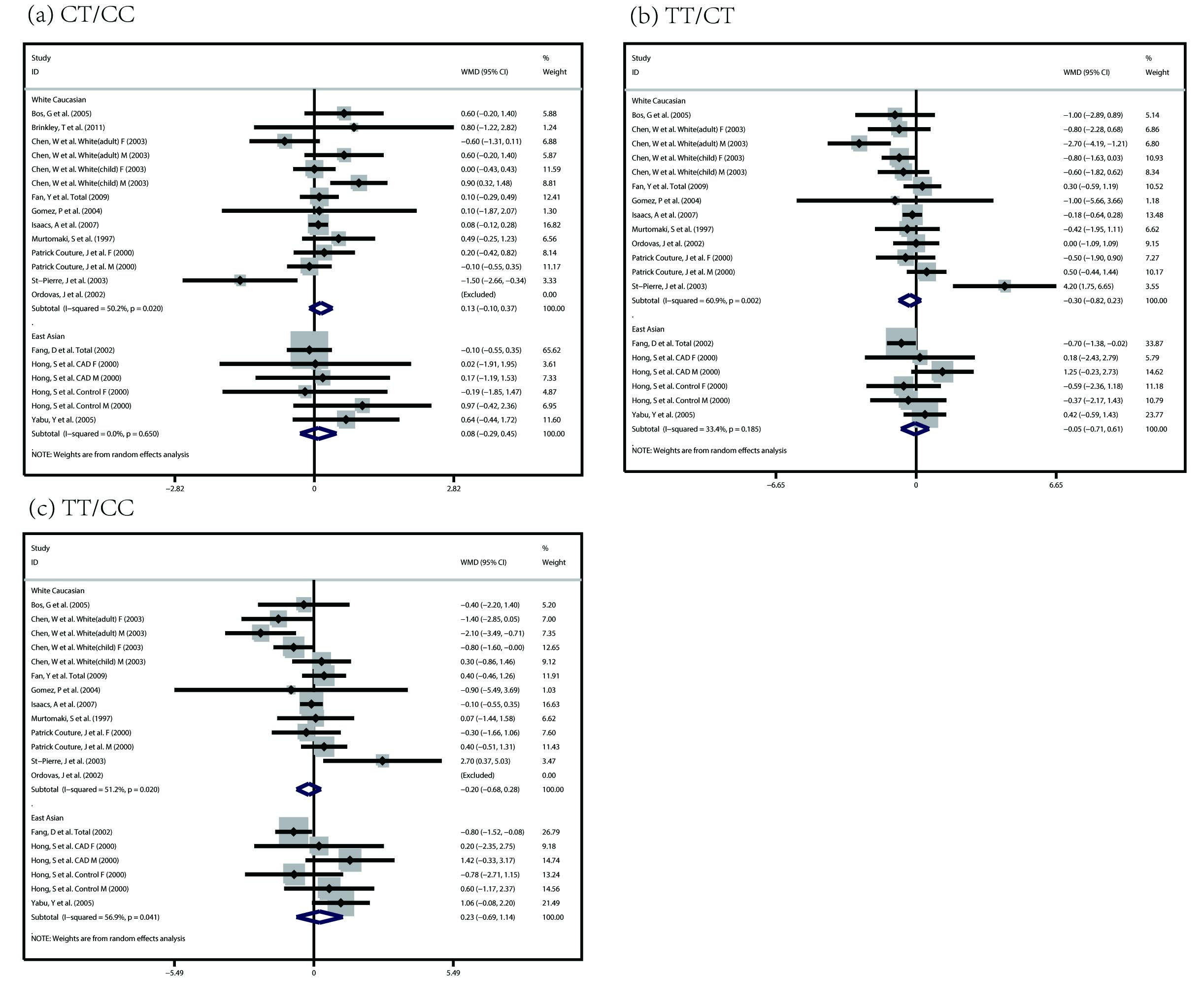

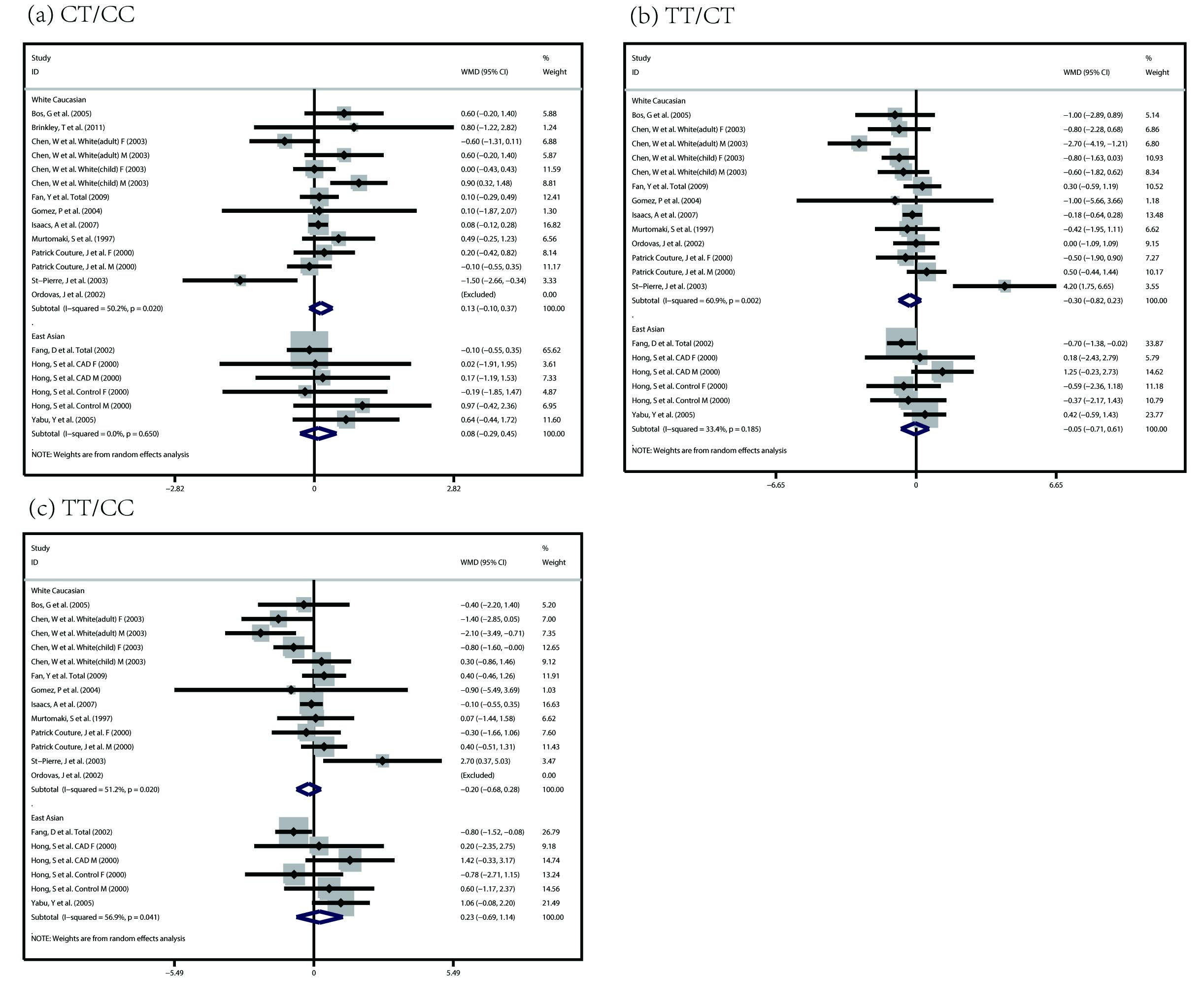


**CT/CC TT/CT TT/CC**

-2.82 0 2.82 -6.65 0 6.65 -5.49 0 5.49

**Figure S4F** Subgroup analysis of HDL-c by gender in C-514T

**CT/CC TT/CT**


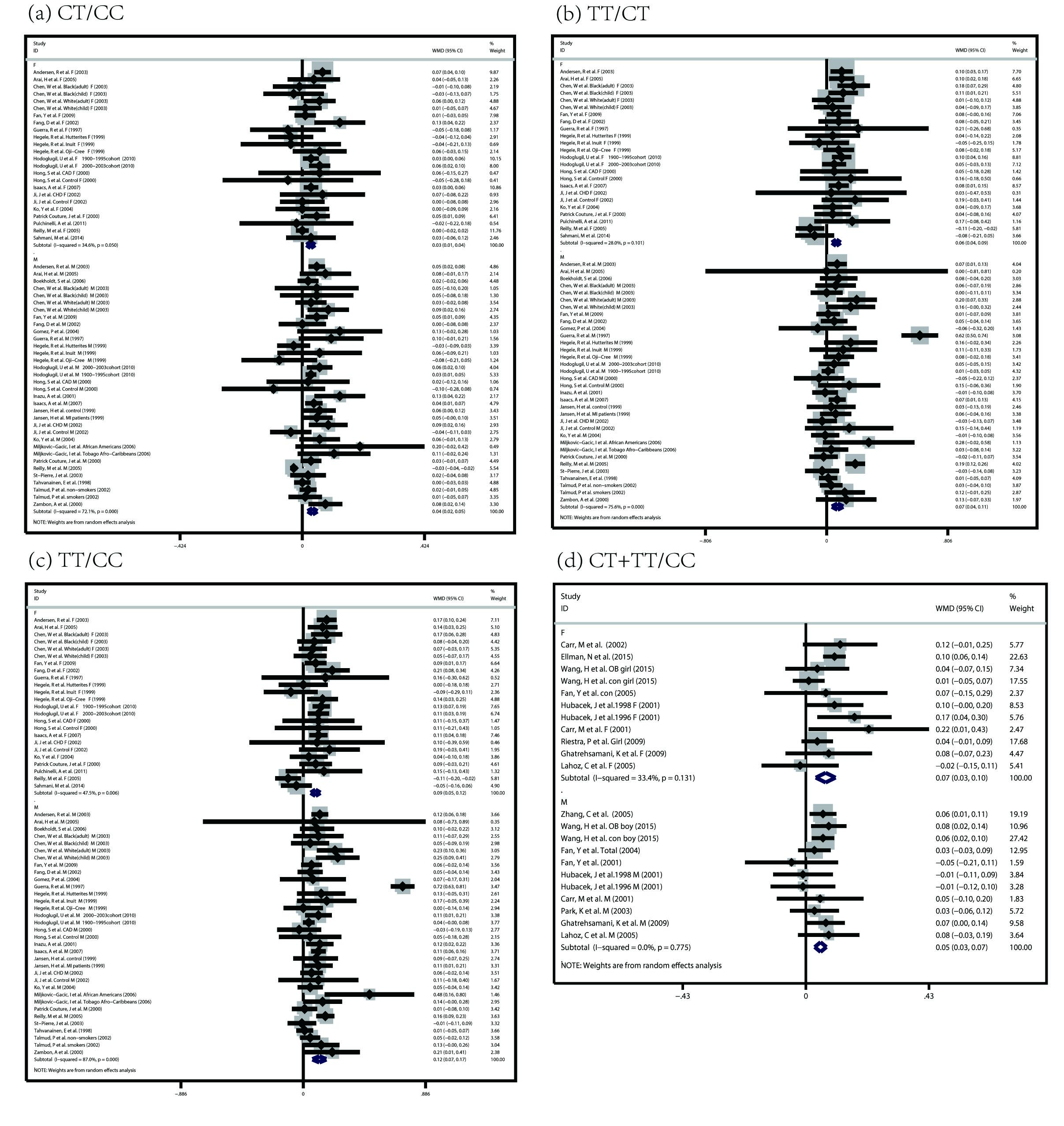

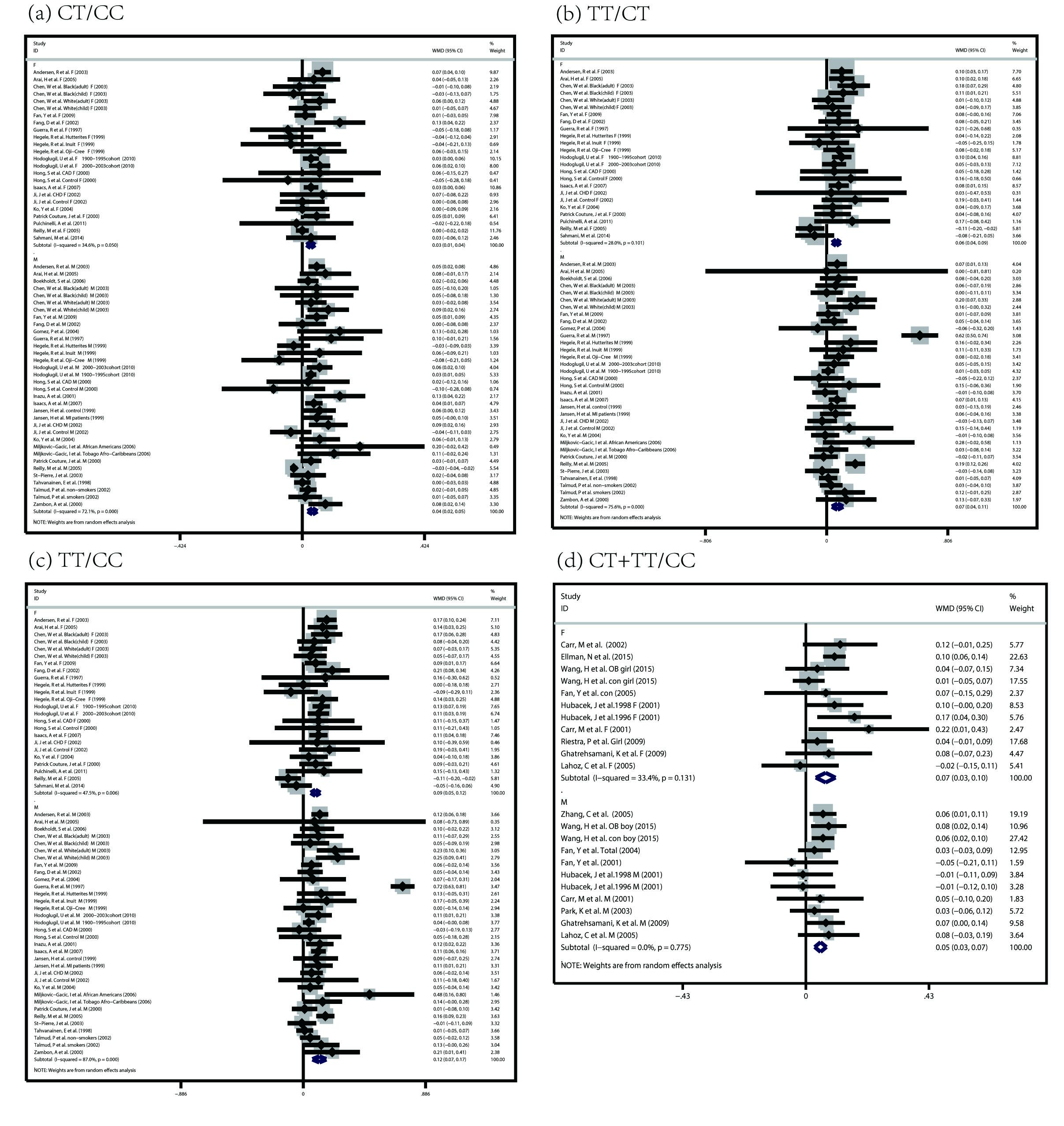


-.424 0 .424 -.806 0 .806


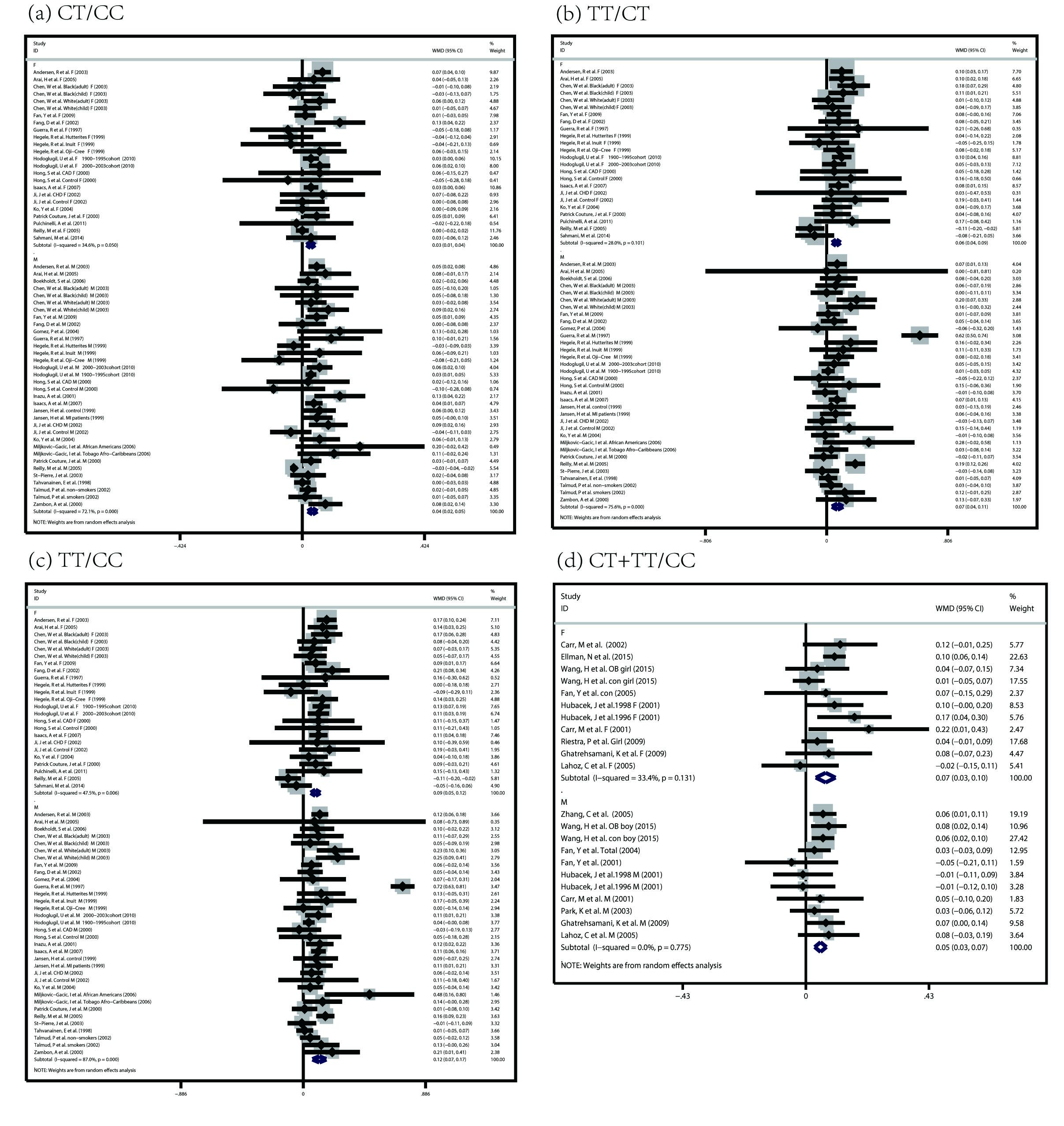

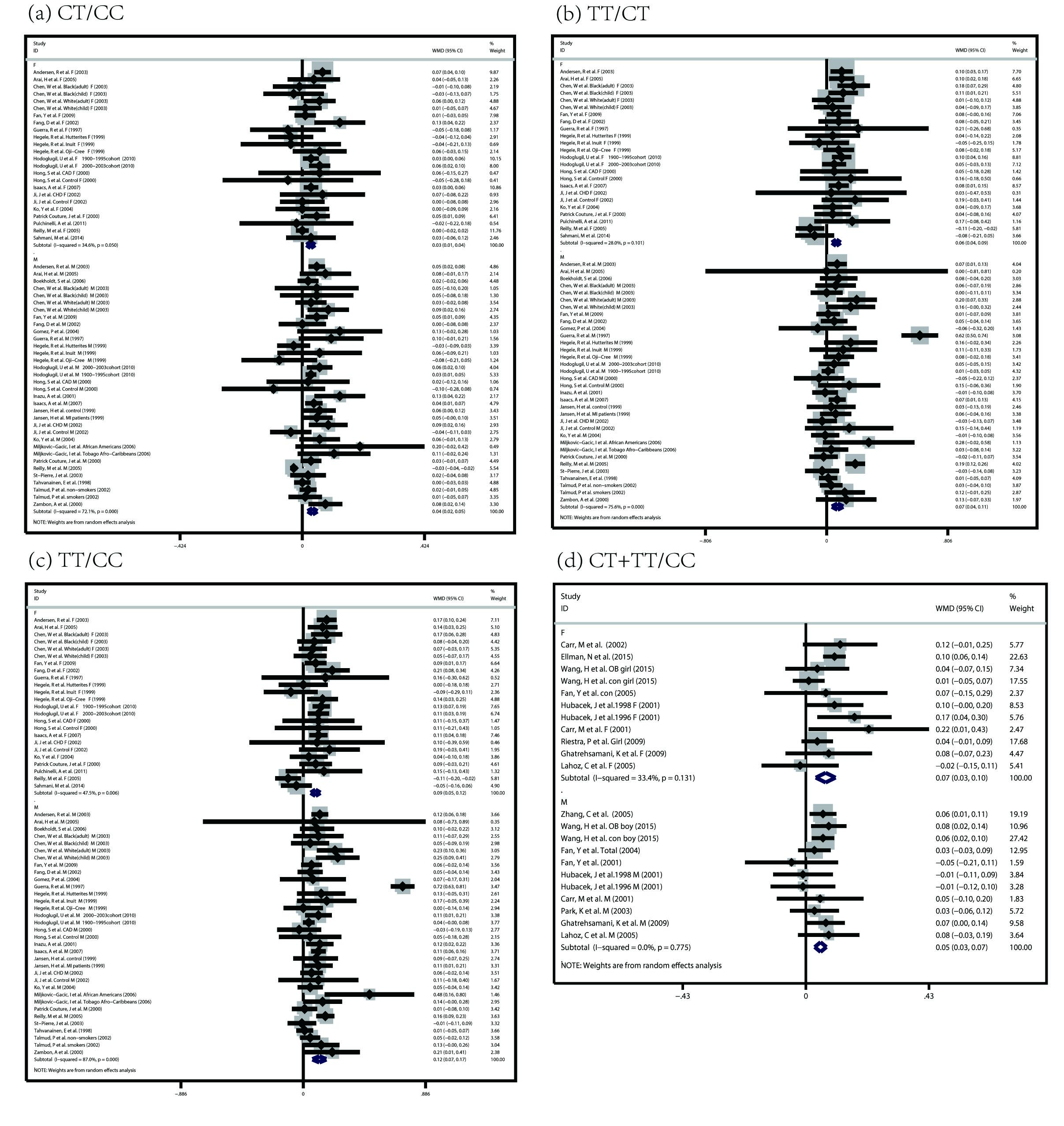


**TT/CC CT + TT/CC**

-.43 0 .43

-.886 0 .886

**Figure S4G** Subgroup analysis of LDL-c by gender in C-514T


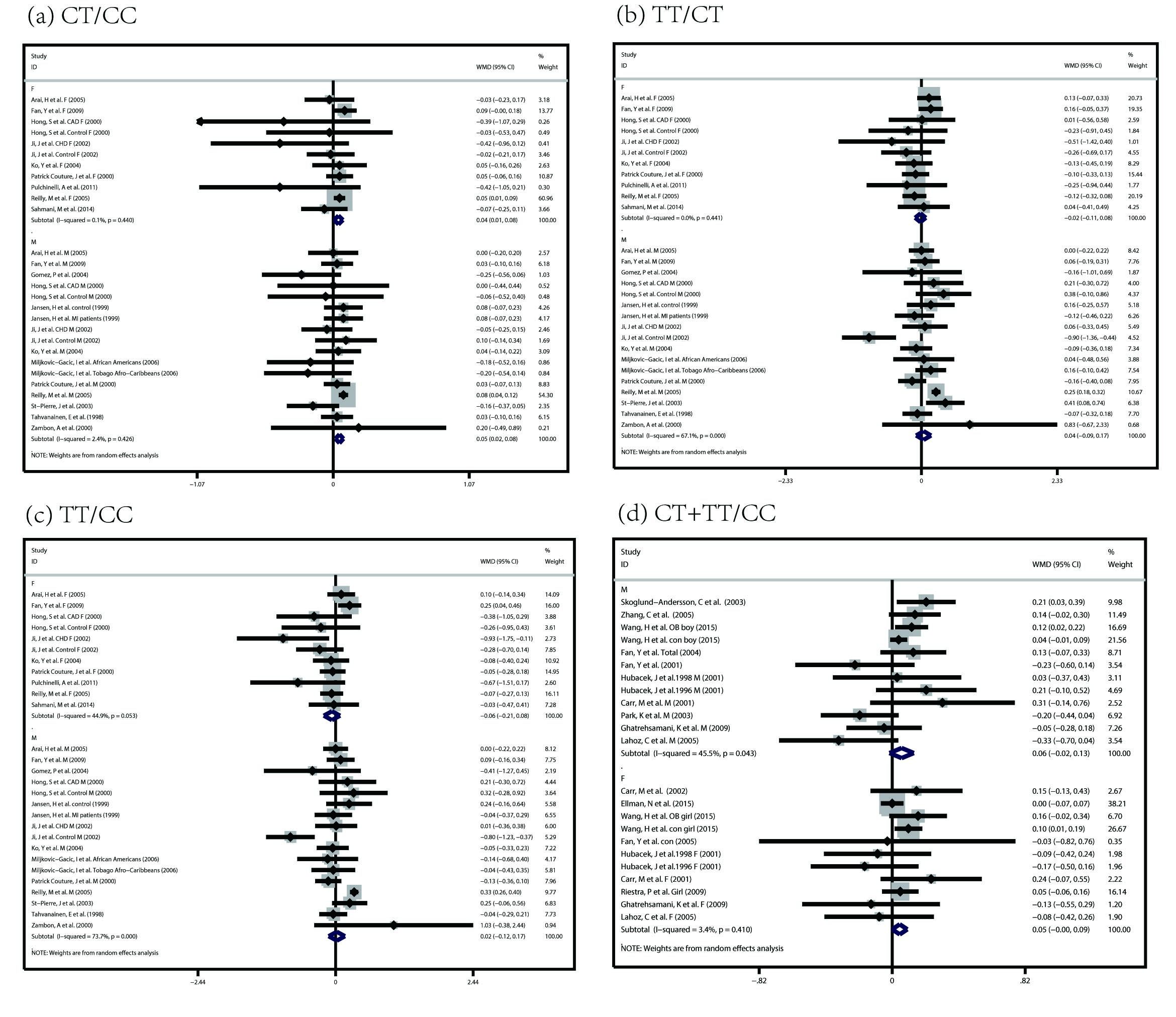


**CT/CC TT/CT**


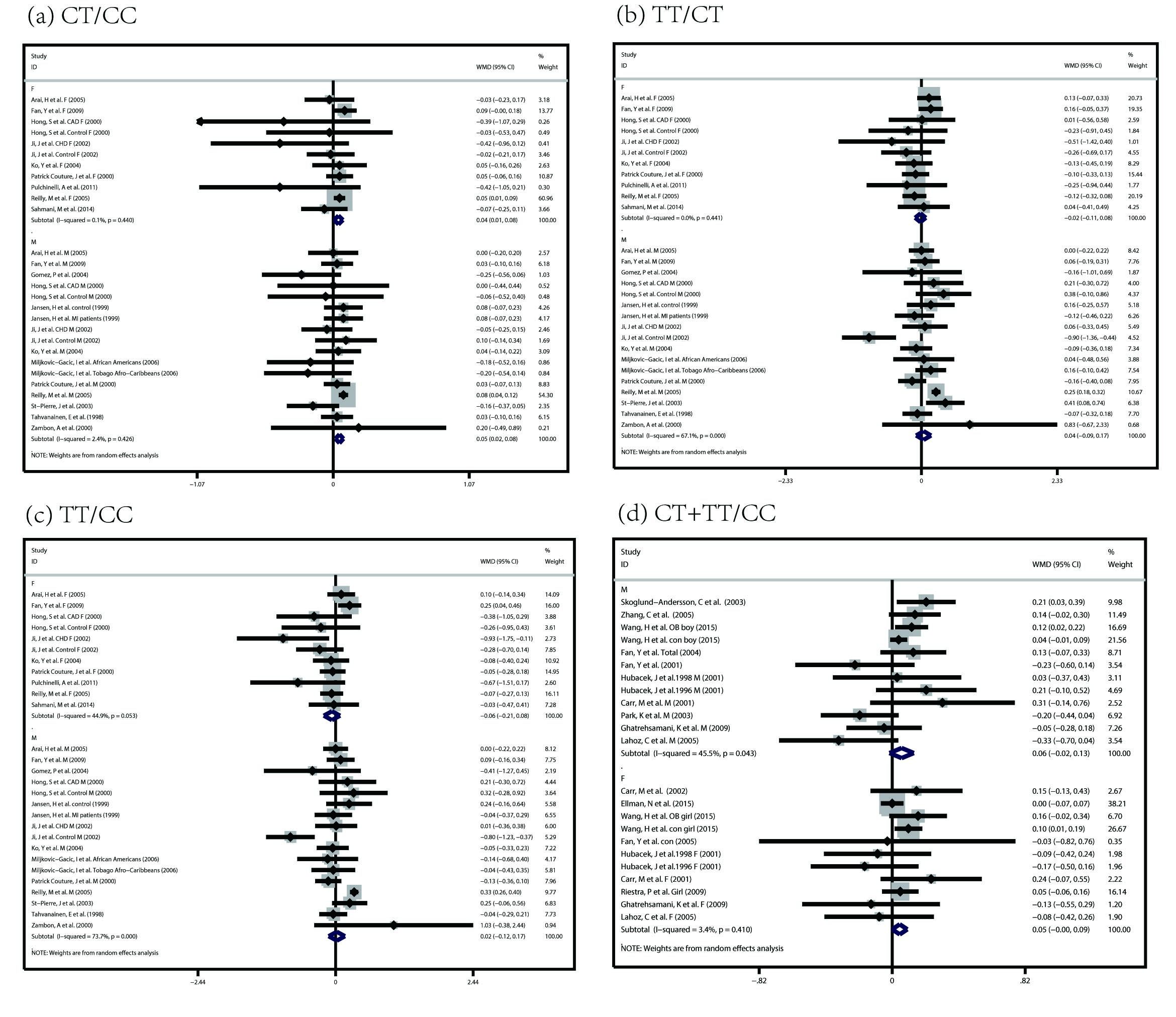


-1.07 0 1.07 -2.33 0 2.33

-2.44 0 2.44 -.82 0 .82

**TT/CC CT + TT/CC**

**Figure S4H** Subgroup analysis of TC by gender in C-514T


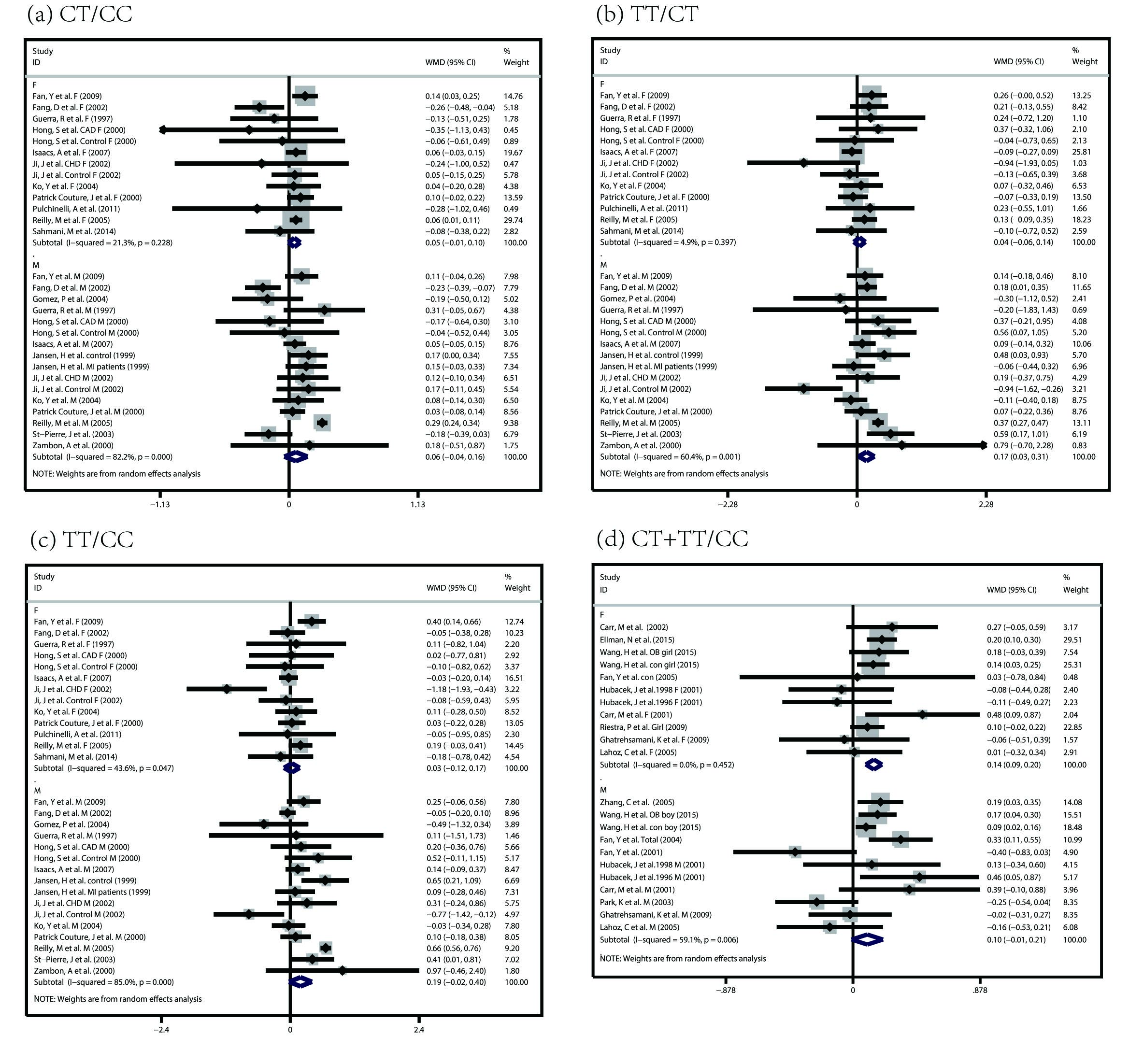


**CT/CC TT/CT**

-1.13 0 1.13 -2.28 0 2.28


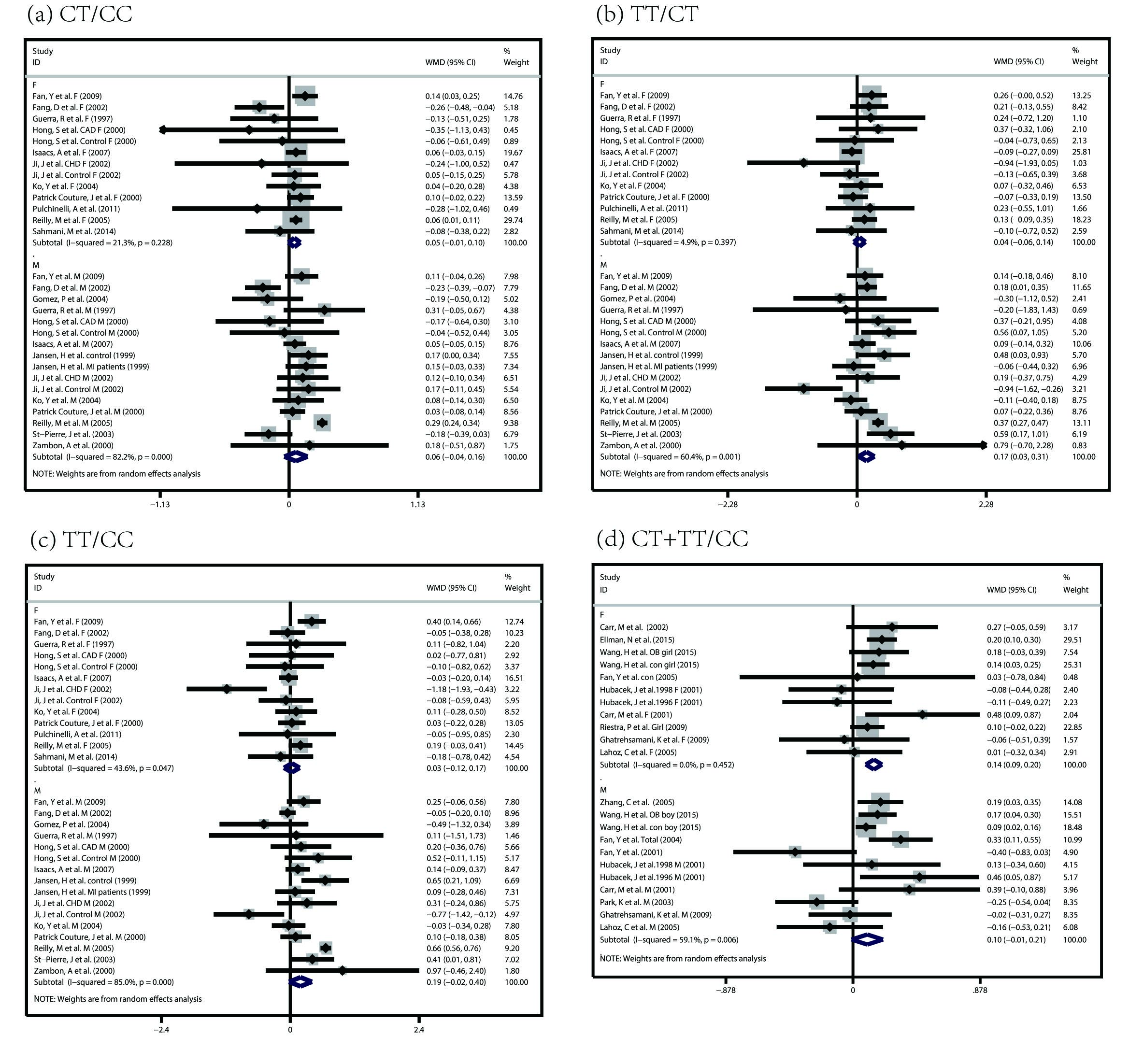

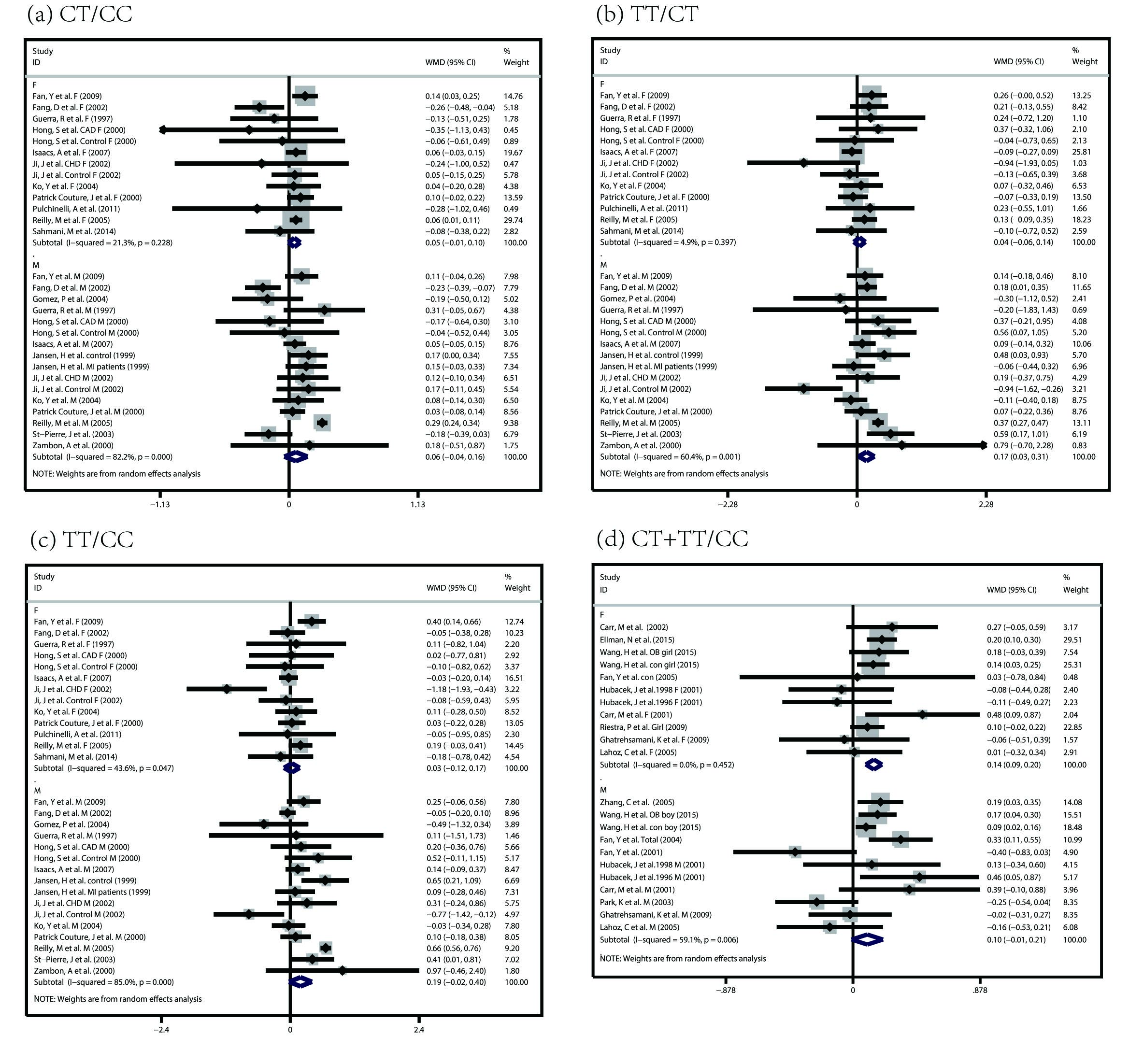


**TT/CC CT + TT/CC**

-.878 0 .878

-2.4 0 2.4


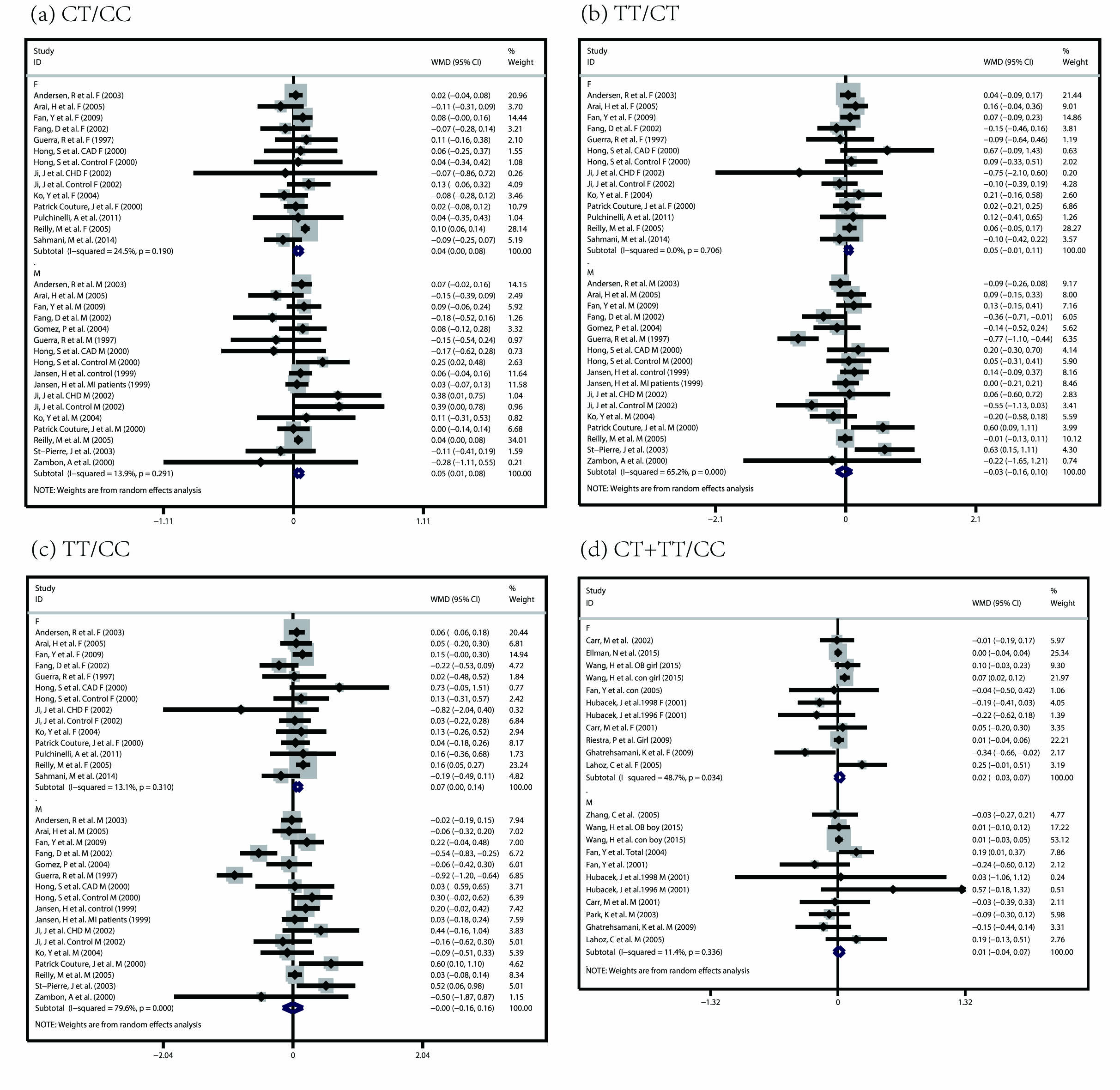
**Figure S4I** Subgroup analysis of TG by gender in C-514T

**CT/CC TT/CT**


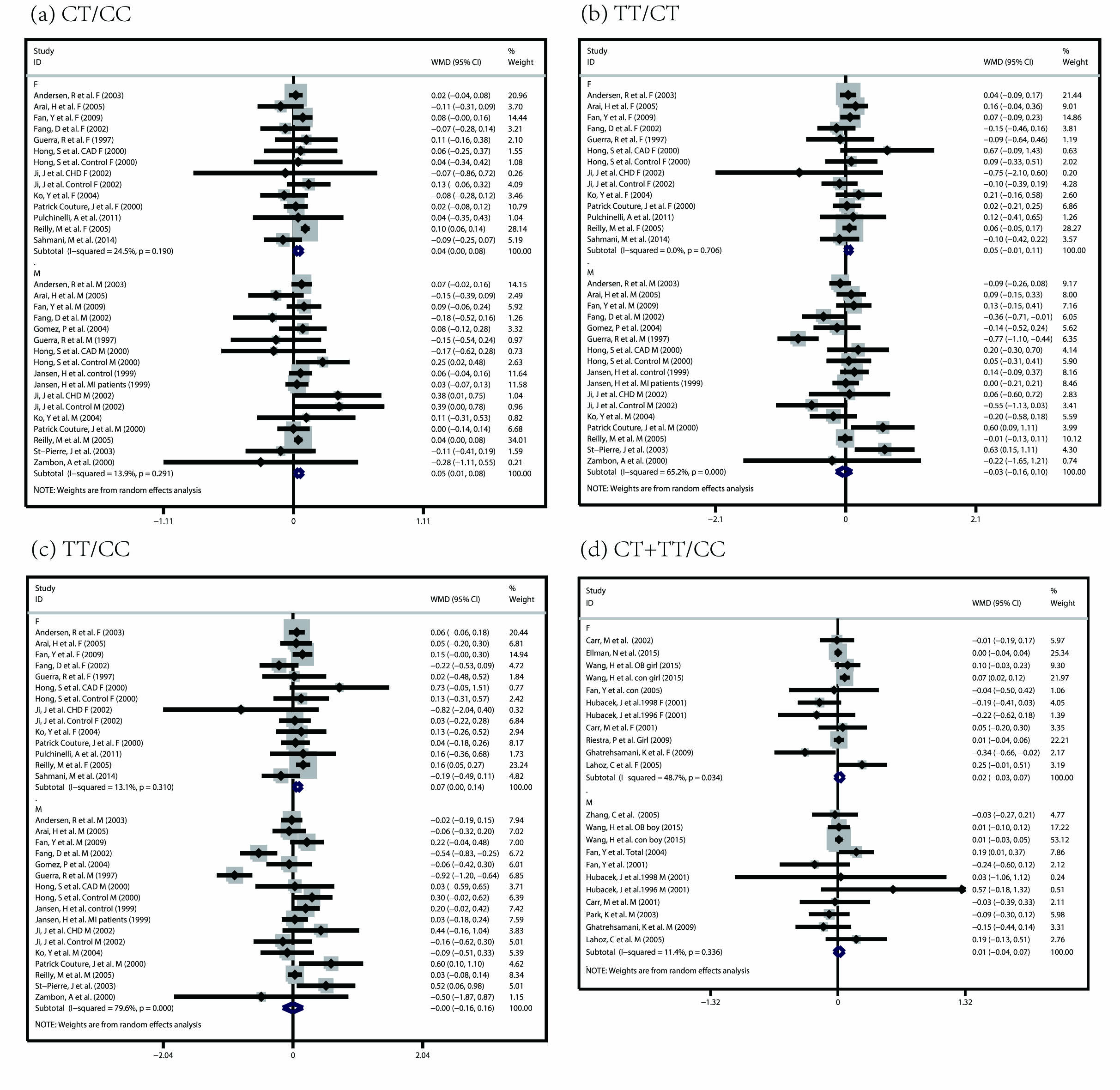

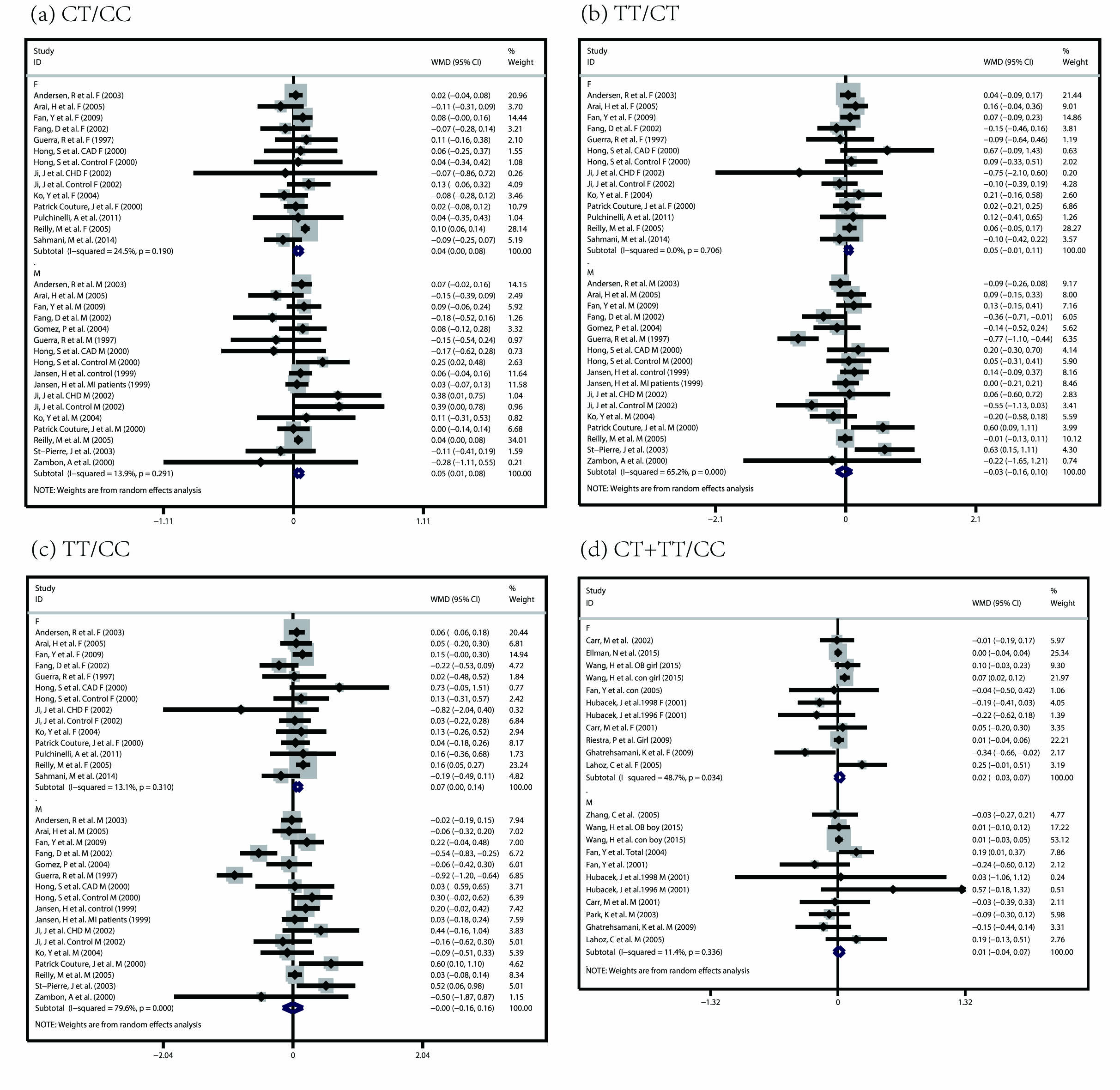


-1.11 0 1.11 -2.1 0 2.1

**TT/CC CT+ TT/CC**

-1.32 0 1.32

-2.04 0 2.04

**Figure S4J** Subgroup analysis of BMI by gender in C-514T


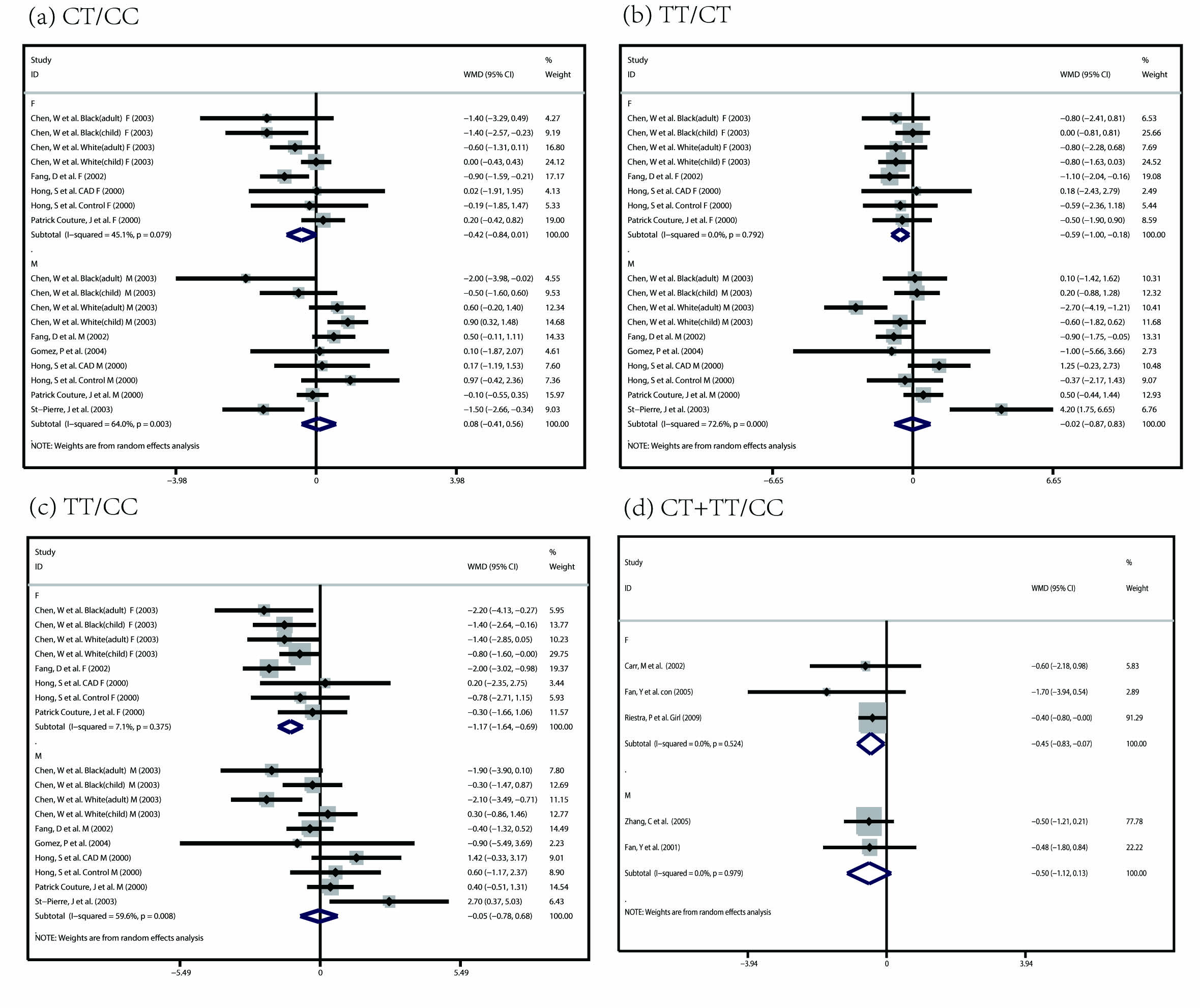


**CT/CC TT/CT**


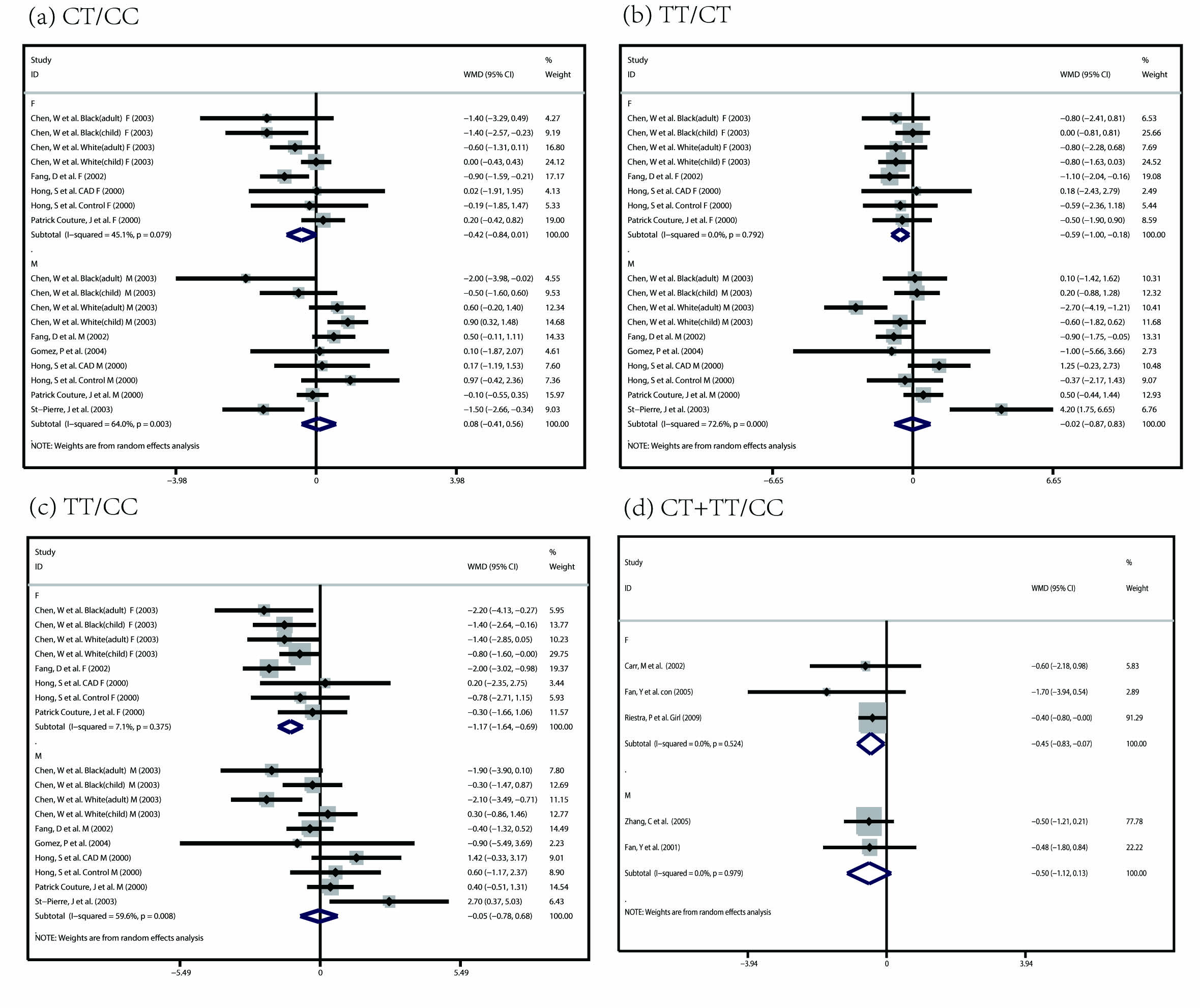

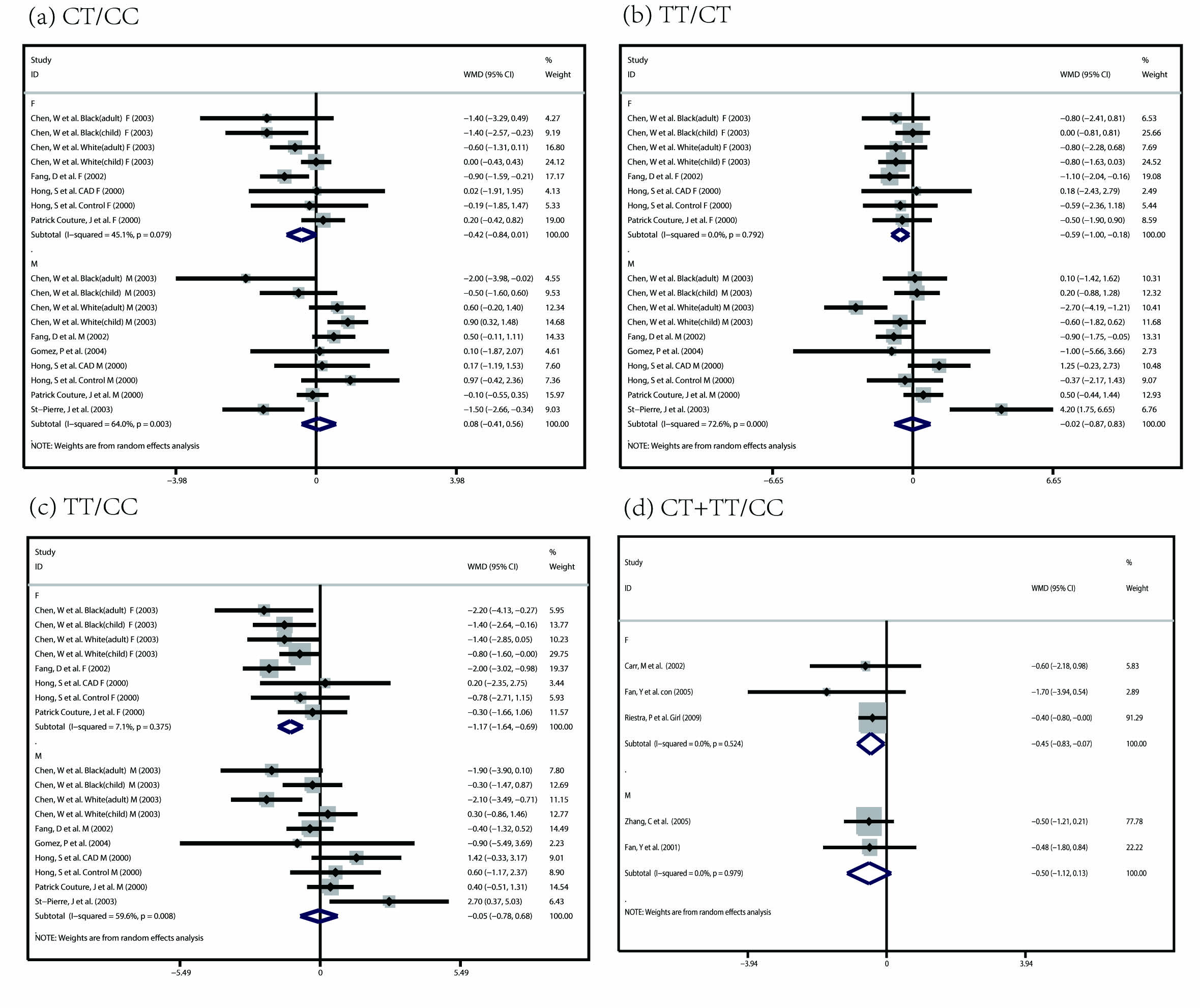


-3.94 0 3.94

-5.49 0 5.49

-3.98 0 3.98 -6.65 0 6.65

**TT/CC CT + TT/CC**

**Figure S4K**Subgroup analysis by gender in G-250A

**HDL-C LDL-C**


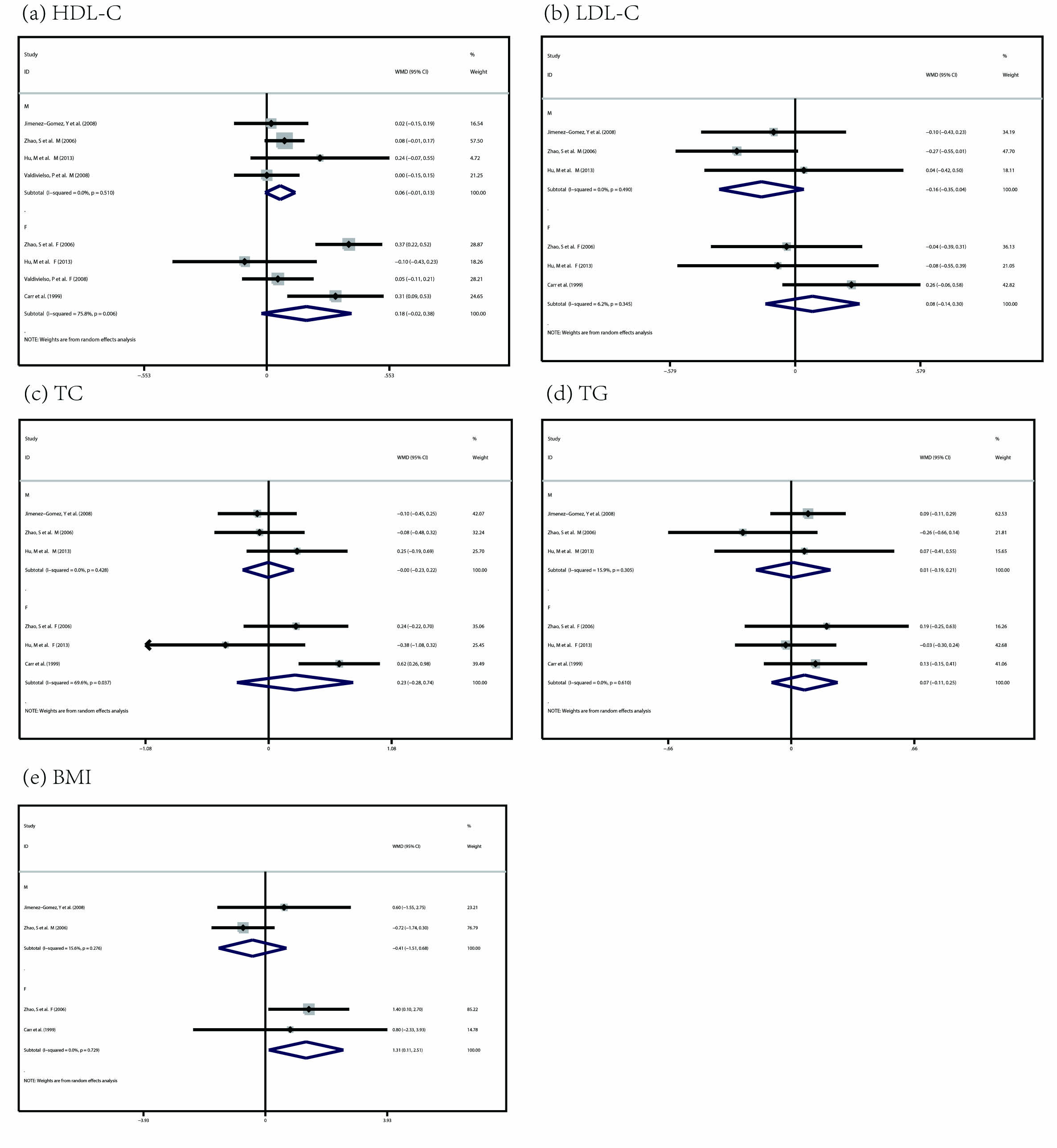

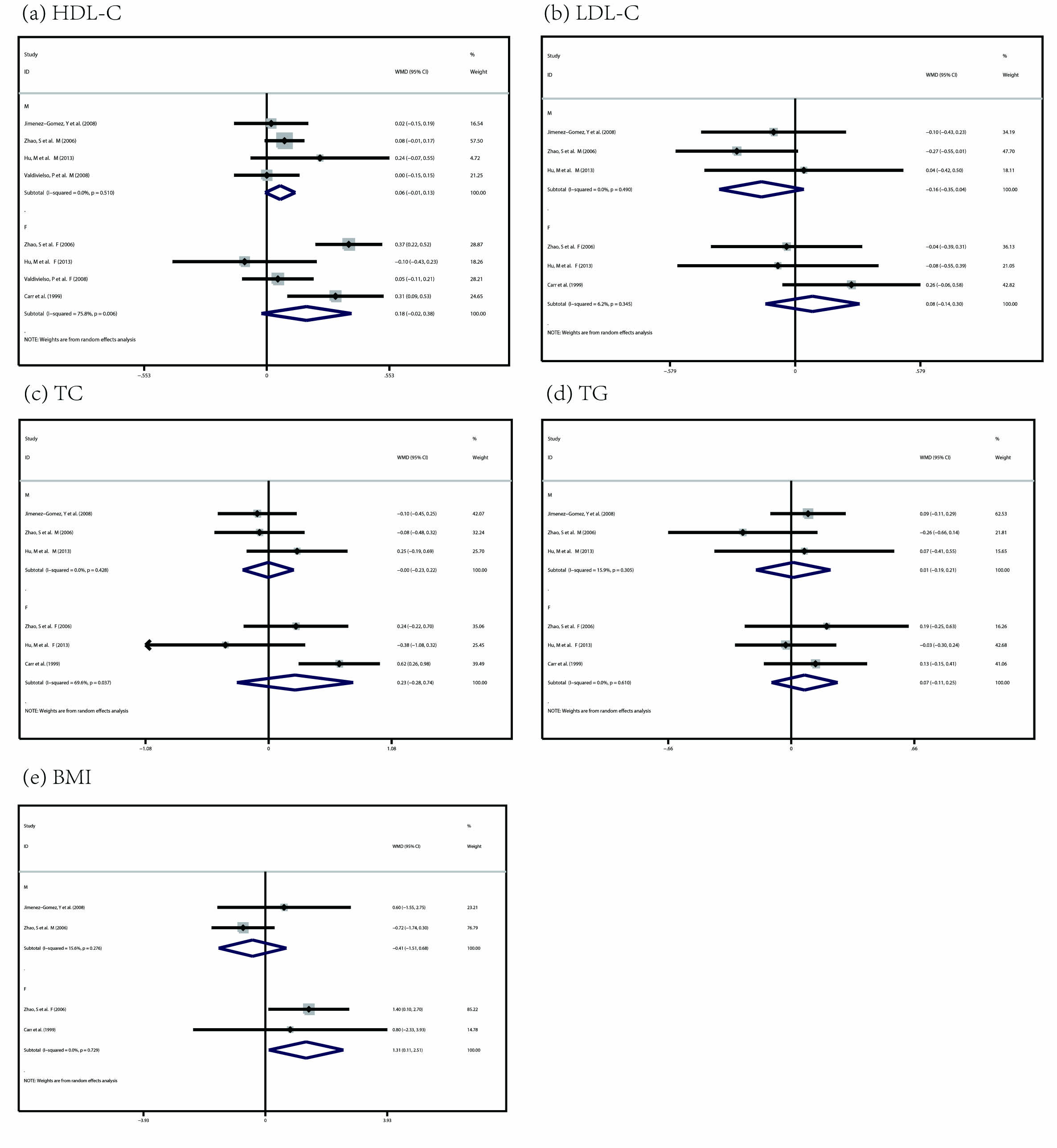


**TC TG**

-.553 0 .553 -.579 0 .579


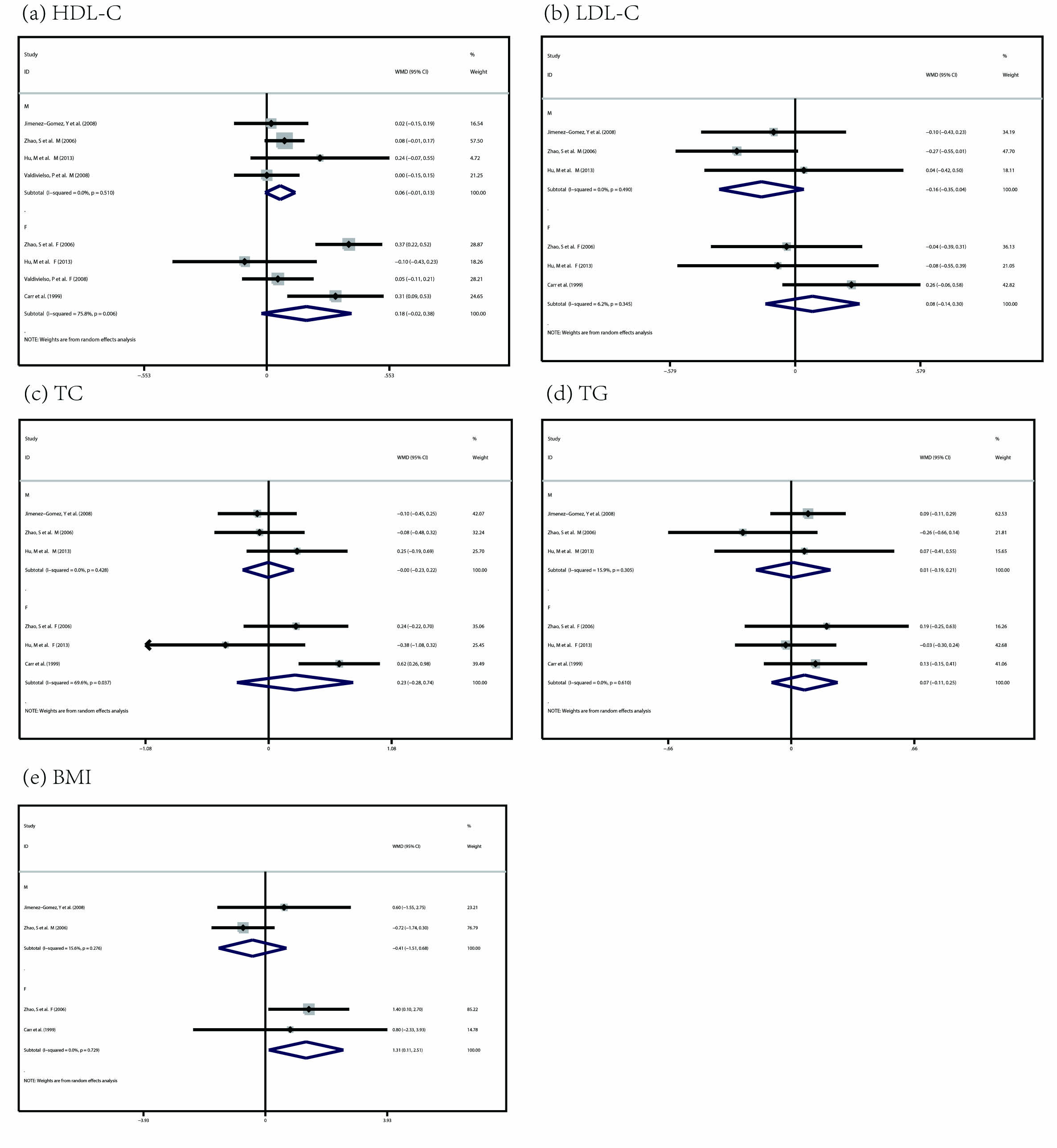


-1.08 0 1.08 -.66 0 .66


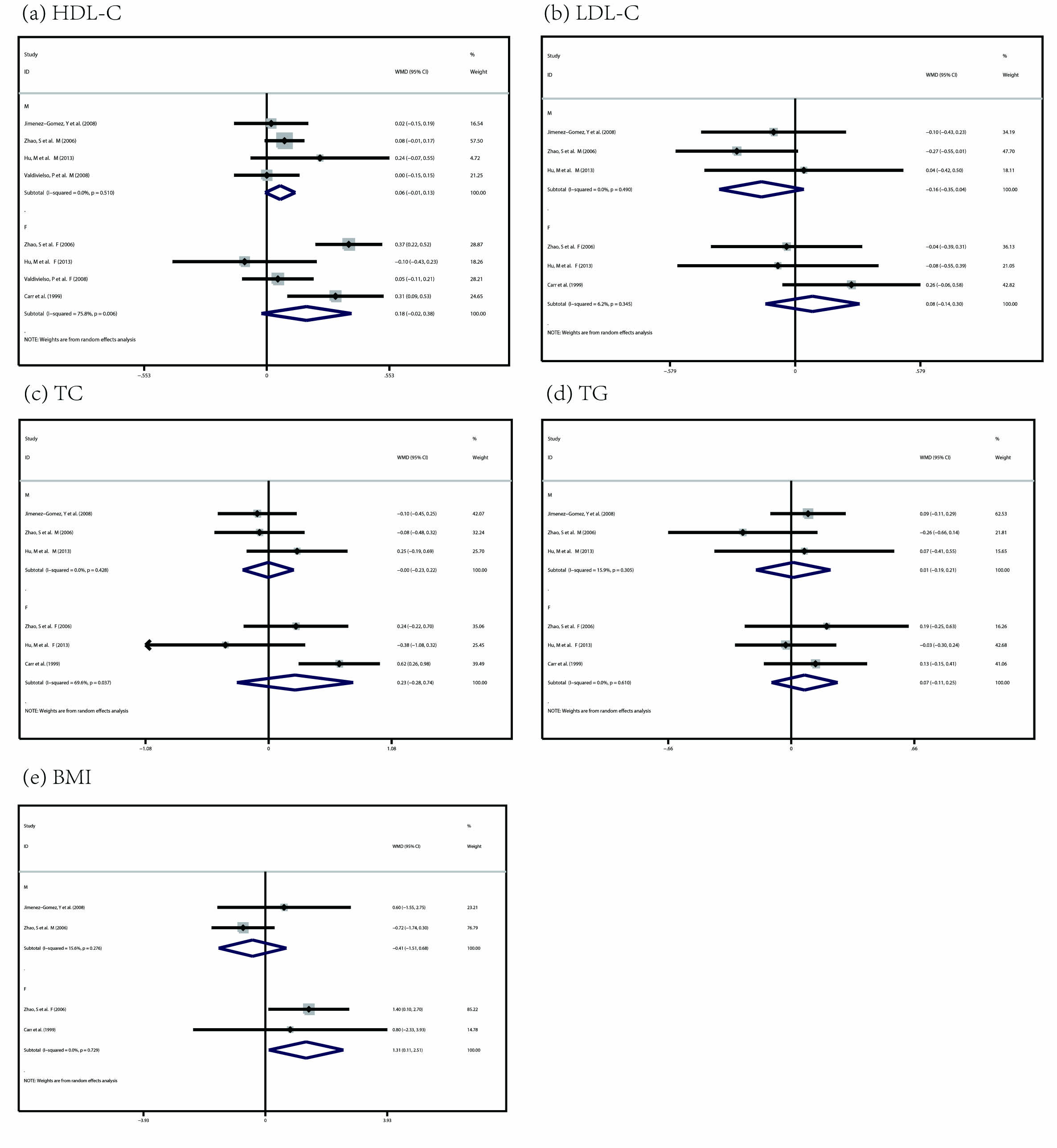


**BMI**

-3.93 0 3.93

**Figure S5** Sensitivity analysis


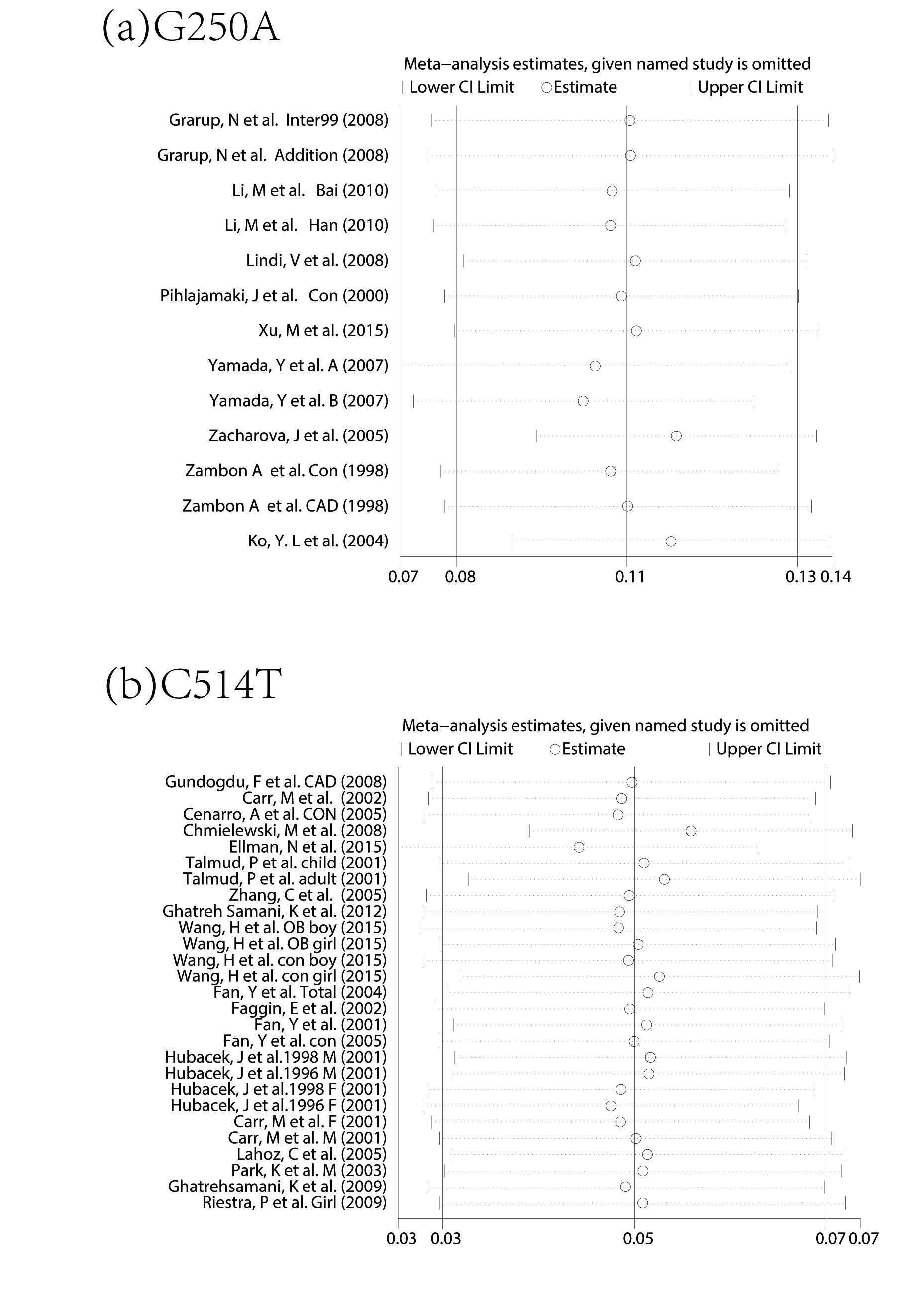


**(A) G250A**

**(B) C514T**

**
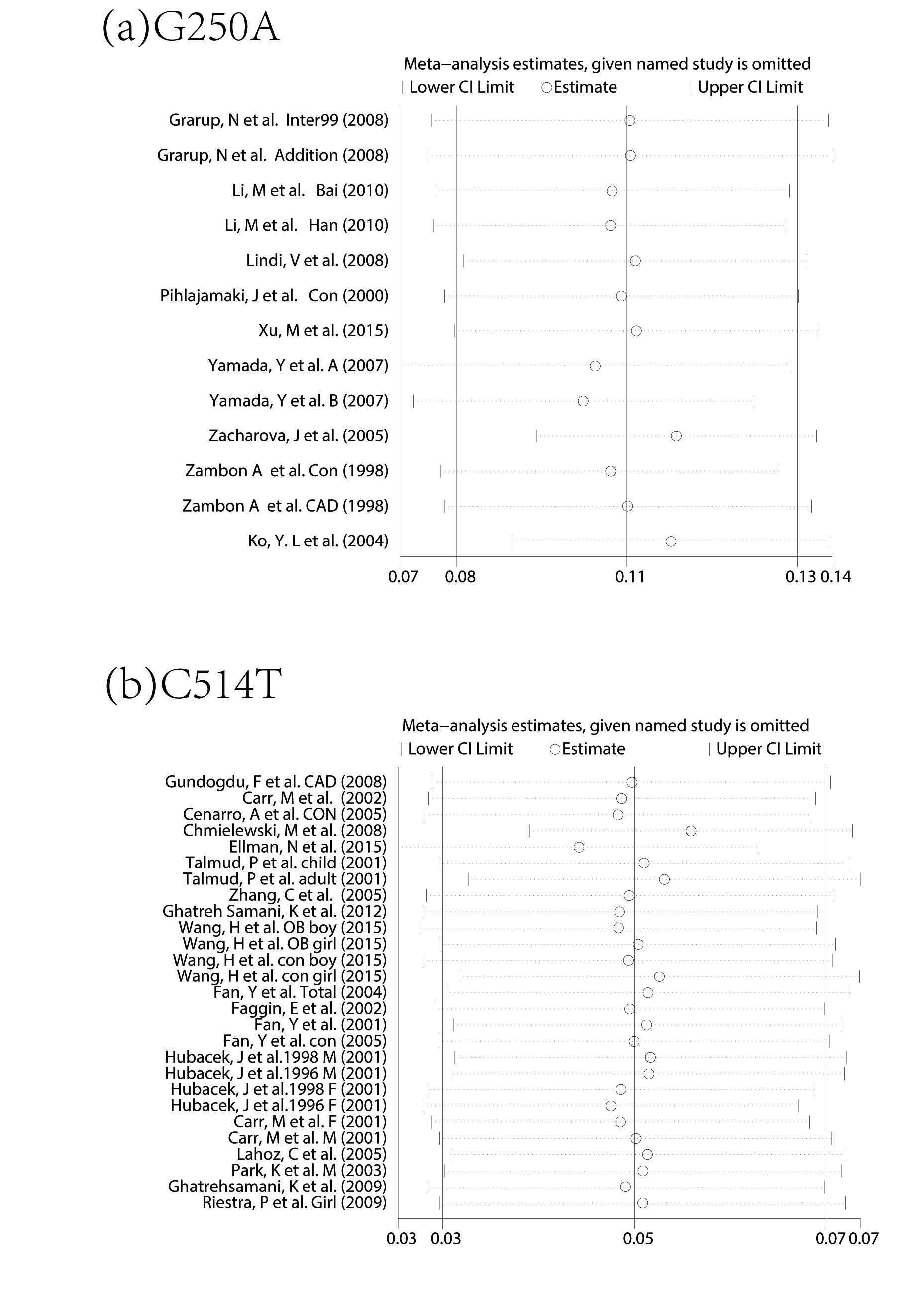
**
