# Supplementary material for: The cross‐sectional study of hepatic lipase SNPs and plasma lipid levels
Source: Food Sci Nutr. 2020 Jan 13;8(2):1162–72. doi: 10.1002/fsn3.1403 (PMC7180388; doi:10.1002/fsn3.1403)
Supplement: Supplementary file 3 [file FSN3-8-1162-s003.docx]

**Table S1** Main characteristics of eligible studies included in this Meta-analysis

| Author | Year | Gender | Race | Age (mean) | Sample  Size | Genotype | P(HWE) | Outcomes | SNP | Ref. |
| --- | --- | --- | --- | --- | --- | --- | --- | --- | --- | --- |
| Andersen et al. | 2003 | M/F | Danish |  | 5566*/3027*/407* | CC/CT/TT | 0.297, 0.373 | HDL-c,TG | C480Tα | 1 |
| Arai et al. | 2005 | M/F | Japanese | 47.6 | 564/1143/560 | CC/CT/TT | 0.689 | HDL-c,LDL-c,TG | C514T^β^ | 2 |
| Ayyappa et al. | 2013 | M/F | Indian | 45 | 430/267/48 | CC/CT/TT | 0.452 | HDL-c,LDL-c,TC,TG | C514T | 3 |
| Ayyobi et al. | 2005 | M/F | American | - | 98/146/60 | CC/CT/TT | 0.672 | HDL-c,LDL-c,TG, | C514T | 4 |
| Baylin et al. | 2010 | M/F | Costarican | 58 | 592/964/384 | CC/CT/TT | 0.813 | HDL-c,LDL-c,TG | C514T | 5 |
| Boekholdt et al. | 2006 | M | Caucasian | 56 | 337/184/25 | CC/CT/TT | 0.986 | HDL-c | C514T | 6 |
| Bos et al. | 2005 | M/F | Dutch | 70 | 275/142/12 | CC/CT/TT | 0.209 | HDL-c,LDL-c,TC,TG,HA | C480T | 7 |
| Brinkley et al. | 2011 | M/F | Caucasian | 58 | 51/25 | CC/CT | 0.086 | HDL-c,LDL-c, | C514T | 8 |
|  |  |  |  |  |  |  |  | TC,TG,,HA, HDL2-c,HDL3-c |  |  |
| Carr et al. | 1999 | F | American | 44.4 | 40/17 | GG/GA+AA | p>0.05 | HDL-c,LDL-c, TC,TG,HA, HDL2-c,HDL3-c | G250A^γ^ | 9 |
| Carr et al. | 2002 | F | American |  | 70/50 | CC/CT+TT | 0.785 | HDL-c, LDL-c,TC,TG,HA, HDL2-c,HDL3-c | C514T | 10 |
| Carr et al. | 2001 | M/F | American |  | 64*/43* | CC/CT+TT | 0.91 | HDL-c, LDL-c,TC,TG,,HA, HDL2-c,HDL3-c | C514T | 11 |
| Cenarro et al. | 2005 | M/F | Spanish | 39.9 | 72/42 | CC/CT+TT | 0.177 | HDL-c,LDL-c,TC,TG | C514T | 12 |
| Chen et al. | 2003 | M/F | American | 4-38 | 2863*/2317*/762* | CC/CT/TT | 0.58，0.967，0.61，0.238，0.962，0.103，0.232，0.25 | HDL-c, | C514T | 13 |
| Chmielewski et al. | 2008 | M/F | Swedish | 54.5 | 160/133 | CC/CT+TT | 0.504 | HDL-c,TG | C480T | 14 |
| Cilingiroglu et al. | 2003 | NA | American | - | 222/126/24 | CC/CT/TT | 0.29 | HDL-c | C514T | 15 |
| Dugi et al. | 2000 | M | Germans | 37.1 | 131/53/11 | CC/CT/TT | 0.054 | HA | C514T | 16 |
| Ellman et al. | 2015 | F | American | 30 | 119/65 | CC/CT+TT | 0.435 | HDL-c,LDL-c,TC,TG, | C514T | 17 |
| Faggin et al. | 2002 | M/F | American | 67.4 | 45/23 | CC/CT+TT | 0.351 | HDL-c,LDL-c,TC,TG, | C514T | 18 |
| Fan et al. | 2001 | M | Finlander | 35 | 26/23 | CC/CT+TT | 0.241 | HDL-c,LDL-c,TC,TG, | C480T | 19 |
| Fan et al. | 2005 | F | Finnish | 61.4 | 17/13 | CC/CT+TT | 0.418 | HDL-c, LDL-c,TC,TG, HDL2-c,HDL3-c | C480T | 20 |
| Fan et al. | 2009 | M/F | Finns | 31.5 | 1122/791/128 | CC/CT/TT | 0.467 | HDL-c,LDL-c,TC,TG, | C480T | 21 |
| Fan et al. | 2004 | M | Finns | - | 191/195 | CC/CT+TT | 0.308 | HDL-c,LDL-c,TC,TG | C480T | 22 |
| Fang et al. | 2002 | M/F | Chinese | 54.3 | 336/380/107 | CC/CT/TT | 0.978 | HDL-c,TC,TG, | C480T | 23 |
| Farid et al. | 2010 | M/F | Tehran's population | 55.8 | 338/106/11 | CC/CT/TT | 0.438 | HDL-c,LDL-c,TC,TG | C514T | 24 |
| Ghatreh Samani et al. | 2012 | M/F | Tabriz's population | 53.9 | 222/120 | CC/CT+TT | 0.268 | HDL-c,LDL-c,TC,TG, | C514T | 25 |
| Ghatreh Samani et al. | 2009 | M/F | Tabriz's population | 53.4 | 202/115 | CC/CT+TT | 0.17 | HDL-c,LDL-c,TC,TG | C514T | 26 |
| Gomez et al. | 2004 | M | Spanish |  | 26/22/3 | CC/CT/TT | 0.553 | HDL-c,LDL-c,TC,TG, | C514T | 27 |
| Grarup et al. | 2008 | M/F | Danish | 53 | 8486*/4809*/697* | GG/GA/AA | 0.075,0.374 | HDL-c,LDL-c,TC,TG, | G250A | 28 |
| Guerra et al. | 1997 | M/F | American Caucasian | - | 97/37/5 | CC/CT/TT | 0.535 | HDL-c,TC,TG | C514T | 29 |
| Gundogdu et al. | 2008 | M/F | Turks | 60 | 131/20 | CC/CT+TT | 0.383 | HDL-c,LDL-c,TC,TG, | C514T | 30 |
| Hegele et al. | 1999 | M/F | Canadian | 35.3 | 559*/484*/148* | CC/CT/TT | 0.369, 0.782,0.095 | HDL-c | C480T | 31 |
| Hodoglugil et al. | 2010 | M/F | Turkish population | 43.1 | 1617*/822*/112* | CC/CT/TT | 0.778,0.568, 0.773,0.961 | HDL-c,HA | C514T | 32 |
| Hong et al. | 2000 | M/F | Korean | 60.3 | 84*/128*/49* | CC/CT/TT | 0.927,0.95, 0.966,0.954 | HDL-c,LDL-c,TC,TG, | C514T | 33 |
| Hu et al. | 2012 | M/F | Chinese | 22.8 | 20/36 | CC/CT+TT | 0.965 | NA | C514T | 34 |
| Hu et al. | 2013 | M/F | Chinese | - | 15*/41* | GG/GA+AA | 0.38,0.19 | HDL-c,LDL-c,TC,TG | G250A | 35 |
| Hubacek et al. | 2001 | M/F | Czech Population | 55.4 | 174*/97* | CC/CT+TT | 0.804,0.069 | HDL-c,LDL-c,TC,TG | C514T | 36 |
| Inazu et al. | 2001 | M | Japanese | 46 | 72/150/74 | CC/CT/TT | 0.816 | HDL-c | C514T | 37 |
| Isaacs et al. | 2007 | M/F | Dutch | 69.3 | 3901/2039/299 | CC/CT/TT | 0.122 | HDL-c,TC, | C514T | 38 |
| Jansen et al. | 1999 | M | European | 22.8 | 468*/288*/34* | CC/CT/TT | 0.415,0.3 | HDL-c,LDL-c,TC,TG | C480T | 39 |
| Ji et al. | 2002 | M/F | Caucasian | 43.9 | 752#*/399#*/51#* | CC/CT/TT | 0.976,0.641, 0.888,0.928 | HDL-c,LDL-c,TC,TG | C514T | 40 |
| Jimenez-Gomez et al. | 2008 | M | Spainish | 21.7 | 30/21 | GG/GA+AA | 0.891 | HDL-c,LDL-c,TC,TG, | G250A | 41 |
| Juo et al. | 2001 | NA | African American | 24.2 | 137/283/158 | CC/CT/TT | 0.639 | HDL-c, LDL-c,TC,TG, HDL2-c,HDL3-c | C514T | 42 |
| Kimura et al. | 2003 | M/F | Japanese | 58.9 | 54/79/50 | CC/CT/TT | 0.065 | HDL-c,TC,TG | C514T | 43 |
| Ko et al. | 2004 | M/F | Taiwanese–Chinese | 55.5 | 295/331/90 | CC/CT/TT,  GG/GA/AA | 0.848 | HDL-c,LDL-c,TC,TG | G250A, C514T | 44 |
| Lahoz et al. | 2005 | M/F | Spainish | 55 | 142/94 | CC/CT+TT | 0.396 | HDL-c,LDL-c,TC,TG | C514T | 45 |
| Li et al. | 2010 | M/F | Han/Bai Ku Yao | 40.2 | 620*/665*/141* | GG/GA/AA | 0.064,0.106 | HDL-c,LDL-c,TC,TG | G250A | 46 |
| Lindi et al. | 2008 | M/F | european | 48.3 | 88/53/10 | GG/GA/AA | 0.601 | HDL-c, LDL-c,TC,TG,,HA | G250A | 47 |
| Lopez-Rios et al. | 2011 | M/F | Spainish | 55 | 230/227 | GG/GA+AA | 0.947 | HDL-c | G250A | 48 |
| Lopez-Simon et al. | 2009 | M/F | Spanish | 6.7 | 381/218/27 | CC/CT/TT | 0.549 | HDL-c | C514T | 49 |
| McCaskie et al. | 2006 | M/F | Busselton/  European-Australian | 50.5 | 3287*/1750*/266* | CC/CT/TT | 0.274,0.084 | HDL-c,LDL-c,TG | C480T | 50 |
| Miljkovic-Gacic  et al. | 2006 | M | Caucasians/African  Afro-Caribbeans | 73.1 | 406*/307*/114* | CC/CT/TT | 0.764,0.184 | HDL-c,LDL-c | C514T | 51 |
| Murtomaki et al. | 1997 | M/F | Finnish |  | 144/110/16 | CC/CT/TT | 0.401 | HDL-c,LDL-c,TC,TG, | C480T | 52 |
| Nettleton et al. | 2007 | M/F | African American |  | 637/1469/803 | CC/CT/TT | 0.474 | HDL-c,LDL-c,TC,TG | C514T | 53 |
| Nie et al. | 1998 | M | Caucasian/  African American | - | 80*/68*/21* | CC/CT/TT | 0.949,0.676 | HA | C514T | 54 |
| Ordovas et al. | 2002 | M/F | Caucasian | 54.3 | 1359/698/73 | CC/CT/TT | 0.147 | HDL-c, LDL-c,TG, HDL2-c,HDL3-c | C514T | 55 |
| Ou et al. | 2013 | M/F | Chinese | 56.5 | 109*/154* | GG/GA+AA | 0.182,0.472 | HDL-c,LDL-c,TC,TG | G250A | 56 |
| Park et al. | 2003 | M | Koreans | 60.2 | 47/77 | CC/CT+TT | 0.427 | HDL-c,LDL-c,TC,TG | C514T | 57 |
| Patrick Couture  et al. | 2000 | M/F | American | 51.8 | 1690*/872*/105* | CC/CT/TT | 0.388,0.958 | HDL-c, LDL-c,TC,TG, HDL2-c,HDL3-c | C514T | 58 |
| Pihlajamaki et al. | 2000 | M/F | Finnish | 50.7 | 62/40/8 | GG/GA/AA | 0.661 | HDL-c,LDL-c,TC,TG | G250A | 59 |
| Pulchinelli et al. | 2011 | F | Brazilian | 54 | 15/29/14 | CC/CT/TT | 0.998 | HDL-c,LDL-c,TC,TG | C514T | 60 |
| Ramakrishnan et al. | 2011 | M/F | Indian | 23 | 756/434/76 | CC/CT/TT | 0.196 | HDL-c, LDL-c,TC,TG,HDL2-c | C514T | 61 |
| Reilly et al. | 2005 | M/F | Caucasian | 48.5 | 456*/251*/31* | CC/CT/TT | 0.997,0.467 | HDL-c,LDL-c,TC,TG | C514T | 62 |
| Riestra et al. | 2009 | M/F | Spainish | 6.7 | 381/245 | CC/CT+TT | 0.549 | HDL-c,LDL-c,TC,TG, | C514T | 63 |
| Sahmani et al. | 2014 | F | Irani | 29.8 | 130/63/9 | CC/CT/TT | 0.379 | HDL-c,LDL-c,TC,TG | C514T | 64 |
| Skoglund et al. | 2003 | M | north european | 50 | 237/140 | CC/CT+TT | 0.838 | LDL-c | C480T | 65 |
| Smith et al. | 2017 | M/F | Caribbean Hispanics |  | 16/17/8 | CC/CT/TT | 0.377 | HDL-c, LDL-c,TC,TG,BMI | C514T | 66 |
| St-Pierre et al. | 2003 | M | French-Canadian | 42.8 | 146/75/10 | CC/CT/TT | 0.925 | HDL-c, LDL-c,TC,TG, HDL2-c,HDL3-c | C514T | 67 |
| Tahvanainen et al. | 1998 | M | Finland |  | 212/138/26 | CC/CT/TT | 0.585 | HDL-c,LDL-c | C514T | 68 |
| Tai et al. | 2003 | M/F | Indians | 38.9 | 195/155/25 | CC/CT/TT | 0.433 | HDL-c,LDL-c,TC,TG, | C514T | 69 |
| Talmud et al. | 2001 | M/F | Hispanics  /non-Hispanic Caucasian | 11.2,  41.2 | 337*/389* | CC/CT+TT | 0.965,0.987,  0.944,0.848 | HDL-c,LDL-c,TG | C480T | 70 |
| Talmud et al. | 2002 | M | Caucasian |  | 1017*/500*/80* | CC/CT/TT | 0.165,0.246 | HDL-c | C514T | 71 |
| Teran-Garcia  et al. | 2005 | M/F | African America/  Caucasian | - | 338*/256*/68* | CC/CT/TT | 0.724,0.933 | HDL-c,LDL-c,TG | C514T | 72 |
| Todorova et al. | 2004 | M/F | Finnish | 55 | 274/216 | GG/GA+AA | 0.395 | HDL-c,TC,TG, | G250A | 73 |
| Todur et al. | 2013 | M/F | Indian | 53.4 | 132*/80*/24* | CC/CT/TT | 0.186,0.078 | HDL-c,LDL-c, HDL2-c,HDL3-c | C514T | 74 |
| Valdivielso et al. | 2008 | M/F | Spainish | 58.7 | 64/44 | GG/GA+AA | 0.194 | HDL-c,LDL-c,TG, | G250A | 75 |
| Vega et al. | 1998 | M/F | Turkish | 27 | 51*/33*/5* | CC/CT/TT | 0.051,0.219 | HDL-c,LDL-c, TC,TG,HA | C514T | 76 |
| Vega et al. | 1998 | M | Americans African  / Caucasian |  | 38*/32*/13* | CC/CT/TT | 0.768,0.829 | HA | C514T | 77 |
| Verdier et al. | 2013 | NA | France | 60.3 | 322/196/39 | CC/CT/TT | 0.226 | HDL-c,LDL-c,TC,TG | C514T | 78 |
| Verma et al. | 2016 | M/F | Indian |  | 220/22/2 | GG/GA/AA | 0.097 | HDL-c,LDL-c, TC,TG,BMI | G250A | 79 |
| Wang et al. | 2015 | M/F | Chinese |  | 1138/1401/1831 | CC/CT+TT | 0.974,0.969, 0.809,0.595 | HDL-c,LDL-c,TC,TG | C514T | 80 |
| Wu et al. | 2008 | M/F | Chinese | 45.6 | 355/398/114 | CC/CT/TT | 0.883 | HDL-c,LDL-c,TC,TG | C514T | 81 |
| Xu et al. | 2015 | M/F | American | 51 | 413/268/62 | GG/GA/AA | 0.052 | HDL-c,LDL-c,TC,TG, | G250A | 82 |
| Yabu et al. | 2005 | M/F | Japanese | 53.5 | 35/98/59 | CC/CT/TT | 0.608 | HDL-c,TG, | C514T | 83 |
| Yamada et al. | 2007 | M/F | Japanese | 68.6 | 1261*/2630*/1375* | GG/GA/AA,  CC/CT/TT | 0.088,0.192 | HDL-c | G250A, C514T | 84 |
| Zacharova et al. | 2005 | M/F | Caucasian | 54.4 | 438/285/47 | GG/GA/AA | 0.944 | HDL-c,LDL-c,TC,TG, | G250A | 85 |
| Zambon et al. | 2000 | M | American |  | 25/20/4 | CC/CT/TT | 1 | HDL-c,LDL-c,TC, TG,HA | C514T | 86 |
| Zambon et al. | 1998 | M/F | Caucasian | 33 | 72*/44*/8* | GG/GA/AA | 0.492,0.86 | HDL-c,LDL-c,TC,HA | G250A | 87 |
| Zhang et al. | 2005 | M | US male | 62.1 | 475/277 | CC/CT+TT | P>0.10 | HDL-c,LDL-c,TC,TG, | C514T | 88 |
| Zhao et al. | 2006 | M/F | Chinese | 53 | 125/86/19 | GG/GA+AA | 0.444 | HDL-c,LDL-c,TC,TG, | G250A | 89 |

**Notes:** *The sum of subjects under the same genotype, # Discrepancy exists in numbers of subjects in different outcomes, the largest number was recorded in the table. C480Ta and C514Tb represented the same SNP rs 1800588, G250Ar represented SNP rs2070895. HDL-c (high density lipoprotein cholesterol), LDL-c (low density lipoprotein cholesterol), TG (triglyceride), TC (total cholesterol), BMI (body mass index), HA (hepatic lipase activity).

**References**

1. Andersen, R.V., et al., Hepatic lipase promoter SNPs associated with increased HDL cholesterol and paradoxically an increased risk of ischemic heart disease; the Copenhagen City Heart Study. Atherosclerosis Supplements, 2003. 2(2): p. 44.

2. Arai, H., et al., Polymorphisms in four genes related to triglyceride and HDL-cholesterol levels in the general Japanese population in 2000. J Atheroscler Thromb, 2005. 12(5): p. 240-50.

3. Ayyappa, K.A., et al., Association of hepatic lipase gene polymorphisms with hypertriglyceridemia and low high-density lipoprotein-cholesterol levels among South Indian subjects without diabetes. Diabetes technology & therapeutics, 2013. 15(6): p. 503-12.

4. Ayyobi, A.F., et al., Cholesterol ester transfer protein (CETP) Taq1B polymorphism influences the effect of a standardized cardiac rehabilitation program on lipid risk markers. Atherosclerosis, 2005. 181(2): p. 363-9.

5. Baylin, A., et al., Association between hepatic lipase-514 C/T promoter polymorphism and myocardial infarction is modified by history of hypercholesterolemia and waist circumference. Nutrition Metabolism and Cardiovascular Diseases, 2010. 20(7): p. 498-504.

6. Boekholdt, S.M., et al., Common variants of multiple genes that control reverse cholesterol transport together explain only a minor part of the variation of HDL cholesterol levels. Clin Genet, 2006. 69(3): p. 263-70.

7. Bos, G., et al., Interactions of dietary fat intake and the hepatic lipase-480C -> T polymorphism in determining hepatic lipase activity: the Hoorn Study. American Journal of Clinical Nutrition, 2005. 81(4): p. 911-915.

8. Brinkley, T.E., et al., Hepatic lipase gene -514C>T variant is associated with exercise training-induced changes in VLDL and HDL by lipoprotein lipase. J Appl Physiol (1985), 2011. 111(6): p. 1871-6.

9. Carr, M., et al. Contribution of Hepatic Lipase, Lipoprotein Lipase, and Cholesteryl Ester Transfer Protein to LDL and HDL Heterogeneity in Healthy Women. American Heart Association, 2002. 4:667-673.

10. Carr, M.C., et al., A hepatic lipase gene promoter polymorphism attenuates the increase in hepatic lipase activity with increasing intra-abdominal fat in women. Arterioscler Thromb Vasc Biol, 1999. 19(11): p. 2701-7.

11. Carr, M.C., et al., The contribution of intraabdominal fat to gender differences in hepatic lipase activity and low/high density lipoprotein heterogeneity. The Journal of clinical endocrinology and metabolism, 2001. 86(6): p. 2831-7.

12. Cenarro, A., et al., Genetic variation in the hepatic lipase gene is associated with combined hyperlipidemia, plasma lipid concentrations, and lipid-lowering drug response. Am Heart J, 2005. 150(6): p. 1154-62.

13. Chen, W., et al., Hepatic lipase promoter C-514T polymorphism influences serial changes in HDL cholesterol levels since childhood: the Bogalusa Heart Study. Atherosclerosis, 2003. 169(1): p. 175-82.

14. Chmielewski, M., et al., Lipoprotein Lipase 1595 C/G and Hepatic Lipase-480 C/T Polymorphisms - Impact on Lipid Profile in Incident Dialysis Patients. Blood Purification, 2008. 26(6): p. 555-560.

15. Cilingiroglu, M., et al., Endothelial and hepatic lipase gene polymorphisms are associated with plasma levels of high-density lipoprotein cholesterol in lipoprotein coronary atherosclerosis study. Journal of the American College of Cardiology, 2003. 41(6): p. 266A-266A.

16. Dugi, K.A., et al., Low hepatic lipase activity is a risk factor for coronary artery disease. Atherosclerosis, 2000. 151(1): p. 126.

17. Ellman, N., et al., Ethnic differences in the association between lipid metabolism genes and lipid levels in black and white South African women. Atherosclerosis, 2015. 240(2): p. 311-317.

18. Faggin, E., et al., Association between the-514 C -> T polymorphism of the hepatic lipase gene promoter and unstable carotid plaque in patients with severe carotid artery stenosis. Journal of the American College of Cardiology, 2002. 40(6): p. 1059-1066.

19. Fan, Y., et al., Hepatic lipase gene variation is related to coronary reactivity in healthy young men. European journal of clinical investigation, 2001. 31(7): p. 574-80.

20. Fan, Y.M., et al., Hepatic lipase C-480T genotype-dependent benefit from long-term hormone replacement therapy for atherosclerosis progression in postmenopausal women. J Clin Endocrinol Metab, 2005. 90(6): p. 3786-92.

21. Fan, Y.M., et al. Hepatic lipase promoter C-480T polymorphism is associated with serum lipids levels, but not subclinical atherosclerosis: The Cardiovascular Risk in Young Finns Study. Blackwell Publishing Ltd. 2009. 1:[46-53].

22. Fan, Y.M., et al., Hepatic lipase C-480T polymorphism modifies the effect of HDL cholesterol on the risk of acute myocardial infarction in men: a prospective population based study. Journal of Medical Genetics, 2004. 41(3).

23. Fang, D.Z. and B.W. Liu, Polymorphism of HL +1075C, but not -480T, is associated with plasma high density lipoprotein cholesterol and apolipoprotein AI in men of a Chinese population. Atherosclerosis, 2002. 161(2): p. 417-24.

24. Farid, M.A.K., et al., Association between CETP Taq1B and LIPC-514C/T polymorphisms with the serum lipid levels in a group of Tehran's population: a cross sectional study. Lipids in Health and Disease, 2010. 9: p. 96 .

25. Ghatreh Samani, K., et al., The -514C/T Polymorphism of Hepatic Lipase Gene among Iranian Patients with Coronary Heart Disease. Iranian journal of public health, 2012. 41(1): p. 59-65.

26. Ghatrehsamani, K., et al., Combined Hepatic Lipase-514C/T and Cholesteryl Ester Transfer Protein I405V Polymorphisms Are Associated with the Risk of Coronary Artery Disease. Genetic Testing and Molecular Biomarkers, 2009. 13(6): p. 809-815.

27. Gomez, P., et al., Influence of the-514C/T polymorphism in the promoter of the hepatic lipase gene on postprandial lipoprotein metabolism. Atherosclerosis, 2004. 174(1): p. 73-79.

28. Grarup, N., et al., The -250G>A promoter variant in hepatic lipase associates with elevated fasting serum high-density lipoprotein cholesterol modulated by interaction with physical activity in a study of 16,156 Danish subjects. J Clin Endocrinol Metab, 2008. 93(6): p. 2294-9.

29. Guerra, R., et al., A hepatic lipase (LIPC) allele associated with high plasma concentrations of high density lipoprotein cholesterol. Proceedings of the National Academy of Sciences of the United States of America, 1997. 94(9): p. 4532-7.

30. Gundogdu, F., et al., Association between -514C-->T polymorphism of the hepatic lipase gene and coronary artery disease in a Turkish population. Acta Cardiol, 2008. 63(2): p. 197-202.

31. Hegele, R.A., et al., Absence of association between genetic variation in the LIPC gene promoter and plasma lipoproteins in three Canadian populations. Atherosclerosis, 1999. 146(1): p. 153-160.

32. Hodoglugil, U., D.W. Williamson, and R.W. Mahley, Polymorphisms in the hepatic lipase gene affect plasma HDL-cholesterol levels in a Turkish population. Journal of Lipid Research, 2010. 51(2): p. 422-430.

33. Hong, S.H., J. Song, and J.Q. Kim, Genetic variations of the hepatic lipase gene in Korean patients with coronary artery disease. Clinical Biochemistry, 2000. 33(4): p. 291-296.

34. Hu, M.S., Z.K. Li, and D.Z. Fang, A high carbohydrate diet induces the beneficial effect of the CC genotype of hepatic lipase C-514T polymorphism on the apoB100/apoAI ratio only in young Chinese males. Scandinavian Journal of Clinical & Laboratory Investigation, 2012. 72(7): p. 563-569.

35. Hu, M.-S., Z.-K. Li, and D.-Z. Fang, The association study of the LIPC -250g/A polymorphism and high-carbohydrate/low-fat diet induced serum lipid and apolipoprotein concentration changes in healthy youth. Sichuan da xue xue bao. Yi xue ban = Journal of Sichuan University. Medical science edition, 2013. 44(5): p. 727-735.

36. Hubacek, J.A., et al., Polymorphisms in the lipoprotein lipase and hepatic lipase genes and plasma lipid values in the Czech population. Physiological research / Academia Scientiarum Bohemoslovaca, 2001. 50(4): p. 345-51.

37. Inazu, A., et al., Effects of hepatic lipase gene promoter nucleotide variations on serum HDL cholesterol concentration in the general Japanese population. Journal of human genetics, 2001. 46(4): p. 172-7.

38. Isaacs, A., et al., Epistatic effect of cholesteryl ester transfer protein and hepatic lipase on serum high-density lipoprotein cholesterol levels. J Clin Endocrinol Metab, 2007. 92(7): p. 2680-7.

39. Jansen, H., et al., The T allele of the hepatic lipase promoter variant C-480T is associated with increased easting lipids and HDL and increased preprandial and postprandial LpCIII : B - European Atherosclerosis Research Study (EARS) II. Arteriosclerosis Thrombosis and Vascular Biology, 1999. 19(2): p. 303-308.

40. Ji, J., et al., Hepatic lipase gene -514 C/T polymorphism and premature coronary heart disease. J Cardiovasc Risk, 2002. 9(2): p. 105-13.

41. Jimenez-Gomez, Y., et al., The -250G/A polymorphism in the hepatic lipase gene promoter influences the postprandial lipemic response in healthy men. Nutrition, metabolism, and cardiovascular diseases : NMCD, 2008. 18(3): p. 173-81.

42. Juo, S.H.H., et al., Promoter polymorphisms of hepatic lipase gene influence HDL2 but not HDL3 in African American men: CARDIA study. Journal of Lipid Research, 2001. 42(2): p. 258-264.

43. Kimura, H., et al., Hepatic lipase mutation may reduce vascular disease prevalence in hemodialysis patients with high CETP levels. Kidney Int, 2003. 64(5): p. 1829-37.

44. Ko, Y.L., et al., The interactive effects of hepatic lipase gene promoter polymorphisms with sex and obesity on high-density-lipoprotein cholesterol levels in Taiwanese-Chinese. Atherosclerosis, 2004. 172(1): p. 135-142.

45. Lahoz, C., et al., The -514C/T polymorphism of the hepatic lipase gene significantly modulates the HDL-cholesterol response to statin treatment. Atherosclerosis, 2005. 182(1): p. 129-34.

46. Li, M., et al., Association of LIPC-250G > A polymorphism and several environmental factors with serum lipid levels in the Guangxi Bai Ku Yao and Han populations. Lipids in Health and Disease, 2010. 9: p. 28 .

47. Lindi, V., et al., The G-250A polymorphism in the hepatic lipase gene promoter is associated with changes in hepatic lipase activity and LDL cholesterol: The KANWU Study. Nutr Metab Cardiovasc Dis, 2008. 18(2): p. 88-95.

48. Lopez-Rios, L., et al., Interaction between cholesteryl ester transfer protein and hepatic lipase encoding genes and the risk of type 2 diabetes: results from the Telde study. PloS one, 2011. 6(11): p. e27208.

49. Lopez-Simon, L., et al., Genetic determinants of plasma HDL-cholesterol levels in prepubertal children. Clinica chimica acta; international journal of clinical chemistry, 2009. 403(1-2): p. 203-6.

50. McCaskie, P., et al. The C-480T hepatic lipase polymorphism is associated with HDL-C but not with risk of coronary heart disease. Blackwell Publishing Ltd. 2006. 2:[114-121].

51. Miljkovic-Gacic, I., et al., Lipoprotein subclass and particle size differences in Afro-Caribbeans, African Americans, and white Americans: associations with hepatic lipase gene variation. Metabolism, 2006. 55(1): p. 96-102.

52. Murtomaki, S., et al., Hepatic lipase gene polymorphisms influence plasma HDL levels. Results from Finnish EARS participants. European Atherosclerosis Research Study. Arteriosclerosis, thrombosis, and vascular biology, 1997. 17(10): p. 1879-84.

53. Nettleton, J.A., et al., Associations between HDL-cholesterol and polymorphisms in hepatic lipase and lipoprotein lipase genes are modified by dietary fat intake in African American and White adults. Atherosclerosis, 2007. 194(2): p. e131-e140.

54. Nie, L.C., et al., Body mass index and hepatic lipase gene (LIPC) polymorphism jointly influence postheparin plasma hepatic lipase activity. Journal of Lipid Research, 1998. 39(5): p. 1127-1130.

55. Ordovas, J.M., et al., Dietary fat intake determines the effect of a common polymorphism in the hepatic lipase gene promoter on high-density lipoprotein metabolism - Evidence of a strong dose effect in this gene-nutrient interaction in the Framingham Study. Circulation, 2002. 106(18): p. 2315-2321.

56. Ou, L., et al., Association of the G-250A promoter polymorphism in the hepatic lipase gene with the risk of type 2 diabetes mellitus. Annales d'Endocrinologie, 2013. 74(1): p. 45-48.

57. Park, K.W., et al., Hepatic lipase C514T polymorphism and its relationship with plasma HDL-C levels and coronary artery disease in Koreans. Journal of Biochemistry and Molecular Biology, 2003. 36(2): p. 237-242.

58. Patrick Couture, J.D.O., L. Adrienne Cupples, Carlos Lahoz, Peter W.F. Wilson, and J.M.O. Ernst J. Schaefer, Association of the-514C -> T polymorphism in the hepatic lipase gene (LIPC) promoter with elevated fasting insulin concentrations, but not insulin resistance, in non-diabetic Germans. Hormone and Metabolic Research, 2000. 36(5): p. 303-306.

59. Pihlajamaki, J., et al., G-250A substitution in promoter of hepatic lipase gene is associated with dyslipidemia and insulin resistance in healthy control subjects and in members of families with familial combined hyperlipidemia. Arteriosclerosis, thrombosis, and vascular biology, 2000. 20(7): p. 1789-95.

60. Pulchinelli, A., Jr., et al., Positive association of the hepatic lipase gene polymorphism c.514C > T with estrogen replacement therapy response. Lipids Health Dis, 2011. 10: p. 197.

61. Ramakrishnan, L., et al., Relationship of APOA5, PPARgamma and HL gene variants with serial changes in childhood body mass index and coronary artery disease risk factors in young adulthood. Lipids Health Dis, 2011. 10: p. 68.

62. Reilly, M.P., et al., Higher order lipase gene association with plasma triglycerides. J Lipid Res, 2005. 46(9): p. 1914-22.

63. Riestra, P., et al., Fat intake influences the effect of the hepatic lipase C-514T polymorphism on HDL-cholesterol levels in children. Experimental biology and medicine (Maywood, N.J.), 2009. 234(7): p. 744-9.

64. Sahmani, M., et al., Lack of association between LIPC-514 C/T polymorphism of hepatic lipase and endometriosis in Iranian women. Journal of Obstetrics and Gynaecology Research, 2014. 40(2): p. 479-484.

65. Skoglund-Andersson, C., et al., Influence of common variants in the CETP, LPL, HL and APO E genes on LDL heterogeneity in healthy, middle-aged men. Atherosclerosis, 2003. 167(2): p. 311-317.

66. Smith, C.E., et al., Dietary fat modulation of hepatic lipase variant -514 C/T for lipids: a crossover randomized dietary intervention trial in Caribbean Hispanics. Physiological genomics, 2017. 49(10): p. 592-600.

67. St-Pierre, J., et al., Visceral obesity attenuates the effect of the hepatic lipase-514C > T polymorphism on plasma HDL-cholesterol levels in French-Canadian men. Molecular Genetics and Metabolism, 2003. 78(1): p. 31-36.

68. Tahvanainen, E., et al., Association of variation in hepatic lipase activity with promoter variation in the hepatic lipase gene. The LOCAT Study Invsestigators. The Journal of clinical investigation, 1998. 101(5): p. 956-60.

69. Tai, E.S., et al., Dietary fat interacts with the-514C > T polymorphism in the hepatic lipase gene promoter on plasma lipid profiles in a multiethnic Asian population: The 1998 Singapore National Health Survey. Journal of Nutrition, 2003. 133(11): p. 3399-3408.

70. Talmud, P.J., et al., Age-related effects of genetic variation on lipid levels: The Columbia University BioMarkers Study. Pediatrics, 2001. 108(3): p. E50.

71. Talmud, P.J., et al., Genetic and environmental determinants of plasma high density lipoprotein cholesterol and apolipoprotein AI concentrations in healthy middle-aged men. Ann Hum Genet, 2002. 66(Pt 2): p. 111-24.

72. Teran-Garcia, M., et al., Hepatic lipase gene variant -514C>T is associated with lipoprotein and insulin sensitivity response to regular exercise: the HERITAGE Family Study. Diabetes, 2005. 54(7): p. 2251-5.

73. Todorova, B., et al., The G-250A promoter polymorphism of the hepatic lipase gene predicts the conversion from impaired glucose tolerance to type 2 diabetes mellitus: The Finnish Diabetes Prevention Study. Journal of Clinical Endocrinology & Metabolism, 2004. 89(5): p. 2019-2023.

74. Todur, S.P. and T.F. Ashavaid, Association of CETP and LIPC Gene Polymorphisms with HDL and LDL Sub-fraction Levels in a Group of Indian Subjects: A Cross-Sectional Study. Indian journal of clinical biochemistry : IJCB, 2013. 28(2): p. 116-23.

75. Valdivielso, P., et al., Association of the-250G/A promoter polymorphism of the hepatic lipase gene with the risk of peripheral arterial disease in type 2 diabetic patients. Journal of Diabetes and Its Complications, 2008. 22(4): p. 273-277

76. Vega, G.L., et al., Hepatic lipase activity is lower in African American men than in white American men: effects of 5 ' flanking polymorphism in the hepatic lipase gene (LIPC). Journal of Lipid Research, 1998. 39(1): p. 228-232.

77. Vega, G.L., et al., The -514 polymorphism in the hepatic lipase gene (LIPC) does not influence androgen-mediated stimulation of hepatic lipase activity. Journal of lipid research, 1998. 39(7): p. 1520-4.

78. Verdier, C., et al., Association of Hepatic Lipase-514T Allele with Coronary Artery Disease and Ankle-Brachial Index, Dependence on the Lipoprotein Phenotype: The GENES Study. Plos One, 2013. 8(7): p. 64.

79. Verma, P., et al., The rs2070895 (-250G/A) single nucleotide polymorphism in hepatic lipase (HL) gene and the risk of coronary artery disease in North Indian population: A case-control study. Journal of Clinical and Diagnostic Research, 2016. 10(8): p. GC01-GC06.

80. Wang, H., et al. Gender specific effect of LIPC C-514T polymorphism on obesity and relationship with plasma lipid levels in Chinese children. John Wiley & Sons, Ltd. 2015. 9:2296-2306.

81. Wu, J., et al., Hepatic lipase gene -514C/T polymorphism in the Guangxi Hei Yi Zhuang and Han populations. Lipids, 2008. 43(8): p. 733-40.

82. Xu, M., et al., Dietary Fat Intake Modifies the Effect of a Common Variant in the LIPC Gene on Changes in Serum Lipid Concentrations during a Long-Term Weight-Loss Intervention Trial. Journal of Nutrition, 2015. 145(6): p. 1289-1294.

83. Yabu, Y., et al., C-514T polymorphism in hepatic lipase gene promoter is associated with elevated triglyceride levels and decreasing insulin sensitivity in nondiabetic Japanese subjects. Int J Mol Med, 2005. 16(3): p. 421-5.

84. Yamada, Y., et al., Prediction of genetic risk for dyslipidemia. Genomics, 2007. 90(5): p. 551-558.

85. Zacharova, J., et al., The G-250A substitution in the promoter region of the hepatic lipase gene is associated with the conversion from impaired glucose tolerance to type 2 diabetes: the STOP-NIDDM trial. J Intern Med, 2005. 257(2): p. 185-93.

86. Zambon, A., et al., A common hepatic lipase gene promoter variant determines clinical response to intensive lipid lowering treatment. Atherosclerosis, 2000. 151(1): p. 266.

87. Zambon, A., et al., Common variants in the promoter of the hepatic lipase gene are associated with lower levels of hepatic lipase activity, buoyant LDL, and higher HDL2 cholesterol. Arterioscler Thromb Vasc Biol, 1998. 18(11): p. 1723-9.

88. Zhang, C., et al., Interactions between the -514C->T polymorphism of the hepatic lipase gene and lifestyle factors in relation to HDL concentrations among US diabetic men. Am J Clin Nutr, 2005. 81(6): p. 1429-35.

89. Zhao, S., X. Xie, and S. Nie, The -250G-->A polymorphism in the human hepatic lipase gene promoter affects blood lipids in Chinese. Clinica chimica acta; international journal of clinical chemistry, 2006. 365(1-2): p. 149-52.
